# Diagnosing scaling bottlenecks in 10 community conservation initiatives in southern and eastern Africa

**DOI:** 10.1111/cobi.70149

**Published:** 2025-09-12

**Authors:** Thomas Pienkowski, Matt Clark, Arundhati Jagadish, Aklei Albert, Mohanjeet Brar, Tarn Breedveld, Linda Chinangwa, Deepali Gohil, Deziderius Irumba, Ramzy Kanaan, Rose Peter Kicheleri, Phillip Kihumuro, Wilhelm Andrew Kiwango, Mathew Bukhi Mabele, Paul Matiku, Gimbage Mbeyale, Musingo Tito E. Mbuvi, Arthur Mugisha, Stanley Mwango, Iddi Mwanyoka, Robson Nyirenda, Geoffrey Oula, Jón Geir Pétursson, Taddeo Rusoke, Nelson Turyahabwe, Moses Kazungu, Lessah Mandoloma, Charles Meshack, Kaala B. Moombe, Francis Moyo, Victor K. Muposhi, Amos Ochieng, Edwin Sabuhoro, Anna Spenceley, Emmanuel Sulle, David Mwesigye Tumusiime, Paulo Wilfred, Peadar Brehony, Elias Damtew Assef, Morena Mills

**Affiliations:** ^1^ Centre for Environmental Policy Imperial College London London UK; ^2^ Durrell Institute of Conservation and Ecology University of Kent Canterbury UK; ^3^ Nature Conservation Foundation Mysore India; ^4^ Independent Dodoma Tanzania; ^5^ Gamewatchers & Porini Camps Nairobi Kenya; ^6^ Independent Maasai Mara Kenya; ^7^ WeForest Malawi Mulanje Malawi; ^8^ Converge Nairobi Kenya; ^9^ DIFONA Vision for Nature Limited Kampala Uganda; ^10^ Tetra Tech Lilongwe Malawi; ^11^ Sokoine University of Agriculture Morogoro Tanzania; ^12^ WWF Uganda Kampala Uganda; ^13^ The University of Dodoma Dodoma Tanzania; ^14^ Nature Kenya Nairobi Kenya; ^15^ Kenya Forestry Research Institute (KEFRI) Nairobi Kenya; ^16^ Nkoba Foundation Kampala Uganda; ^17^ BioCarbon Partners Lusaka Zambia; ^18^ Africa Community Project Ltd Lusaka Zambia; ^19^ African Wildlife Foundation Kampala Uganda; ^20^ Environment and Natural Resources University of Iceland Reykjavik Iceland; ^21^ Mountains of the Moon University Fort Portal Tourism City Uganda; ^22^ Makerere University Kampala Uganda; ^23^ Social Sciences in Landscape Research Group, Research Unit Economics and Social Sciences, Swiss Federal Institute for Forest Snow and Landscape Research WSL Birmensdorf Switzerland; ^24^ Department of Environmental Science and Management Lilongwe University of Agriculture and Natural Resources (LUANAR) Lilongwe Malawi; ^25^ Department of Biology, Interdisciplinary Centre for Conservation Science (ICCS) University of Oxford Oxford UK; ^26^ Tanzania Forest Conservation Group Dar es Salaam Tanzania; ^27^ Center for International Forestry Research (CIFOR) Lusaka Zambia; ^28^ Nelson Mandela African Institution of Science and Technology Arusha Tanzania; ^29^ Botswana University of Agriculture and Natural Resources Gaborone Botswana; ^30^ School of Wildlife Conservation African Leadership University Kigali Rwanda; ^31^ Penn State University University Park Pennsylvania USA; ^32^ University of Johannesburg Johannesburg South Africa; ^33^ Arusha Climate and Environmental Research Centre Aga Khan University Dar es Salaam Tanzania; ^34^ The Open University of Tanzania Dar es Salaam Tanzania; ^35^ Sustain East Africa Arusha Tanzania; ^36^ Betty and Gordon Moore Center for Science Conservation International Washington DC USA

**Keywords:** 30 by 30, adoption, community‐based conservation, community‐based natural resource management, engagement, other effective area‐based conservation measures, scaling bottlenecks, scaling up, scaling out, and scaling deep, 30 para el 30, adopción, conservación comunitaria, escala vertical, escalada de cuellos de botella, horizontal y profunda, gestión comunitaria de los recursos naturales, OECMs, otras medidas efectivas de conservación basada en el área, participación, 30x30, 采用, 基于社区的保护, 基于社区的自然资源管理, 参与, 其他有效区域保护措施(OECMs), 扩大范围的瓶颈, 扩大、缩小和加深

## Abstract

Scaling area‐based conservation, including initiatives led or comanaged by Indigenous Peoples and local communities, is a flagship goal of the Kunming‐Montreal Global Biodiversity Framework. Conservationists often aspire to scale initiatives, but this is rarely achieved in practice. Identifying and addressing factors that limit initiative adoption (i.e., bottlenecks) could improve scaling strategies. We used insightsfrom 84 expert surveys to identify potential risk factors and bottlenecks to scaling 10 community, area‐based initiatives in southern and eastern Africa. The number of reported potential risk factors and bottlenecks varied among initiatives. However, unfair benefit sharing, unequal decision‐making, inflexible rules, and top‐down leadership were frequently identified as bottlenecks. Although adopting initiatives had costs (e.g., increased local conflicts, reduced local access to natural resources and cropland), most experts believed these costs were offset by other benefits and thus did not constitute bottlenecks. Our results did not capture local perspectives, but they suggest scaling strategies that strengthen environmental governance may support more socially just and durable approaches to meeting area‐based conservation goals.

## Abstract (es)

Diagnóstico de la escalada de cuellos de botella en diez iniciativas de conservación comunitaria en África occidental

**Resumen:**

La ampliación de la conservación basada en áreas, incluidas las iniciativas dirigidas o cogestionadas por pueblos indígenas y comunidades locales, es uno de los objetivos emblemáticos del Marco Mundial de Kunming‐Montreal para la Diversidad Biológica. Los conservacionistas suelen aspirar a ampliar las iniciativas, pero esto rara vez se consigue en la práctica. Identificar y abordar los factores que limitan la adopción de iniciativas (es decir, los obstáculos) podría mejorar las estrategias de ampliación. Utilizamos los conocimientos obtenidos de encuestas a 84 expertos para identificar los posibles factores de riesgo y obstáculos para ampliar 10 iniciativas comunitarias basadas en áreas en el sur y el este de África. El número de posibles factores de riesgo y obstáculos notificados varió entre las iniciativas. Sin embargo, con frecuencia identificamos como obstáculos la distribución injusta de los beneficios, la toma desigual de decisiones, las normas inflexibles y el liderazgo vertical. Aunque la adopción de iniciativas tenía costos (p. ej., aumento de los conflictos locales, reducción del acceso local a los recursos naturales y las tierras de cultivo), la mayoría de los expertos consideraban que estos costos se veían compensados por otros beneficios y, por lo tanto, no eran un obstáculo. Nuestros resultados no reflejaban las perspectivas locales, pero sugieren que las estrategias de ampliación que refuerzan la gobernanza ambiental pueden apoyar enfoques más justos y duraderos desde el punto de vista social para alcanzar los objetivos de conservación basados en el área.

## 确定非洲南部和东部十项社区保护倡议的规模化瓶颈

**【摘要】** 扩大基于区域的保护范围, 包括由土著人民和当地社区领导或共同管理的保护倡议, 是《昆明‐蒙特利尔全球生物多样性框架》的旗舰目标之一。保护实践者通常期望扩大保护倡议的范围, 但在实践中却很少能实现这一点。识别并解决限制保护倡议得到采用的因素(即瓶颈), 有助于扩大推广这些战略。我们利用从84项专家调查中收集到的见解, 确定了在非洲南部和东部扩大10项基于社区和区域的保护倡议的潜在风险因素和瓶颈。不同倡议的潜在风险因素和瓶颈的数量存在差异。然而, 常被指出的瓶颈包括不公平的利益分享、不平等的决策、不灵活的规则, 以及自上而下的领导方式。尽管采取这些保护倡议需要付出代价(如增加地方冲突、减少地方对自然资源和耕地的使用), 但大多数专家认为这些代价已被其他收益所补偿, 因此并不构成瓶颈限制。我们的研究结果没有收集到当地社区的观点, 但这些结果表明, 加强环境治理的扩展战略可能有助于采纳更具社会公正性和持续性的方法, 以实现基于区域的保护目标。**【翻译:胡怡思;审校:聂永刚】**

## INTRODUCTION

Globally, governments have committed to massively scale out (i.e., expanding an initiative to reach additional people or locations) area‐based conservation to 30% of the world's surface by 2030 (e.g., Gurney et al., 2023; UN, 2022). Multiple forms of area‐based conservation could help achieve this 30×30 goal, which is expected to affect the lives of hundreds of millions of people (Allan et al., 2022; Schleicher et al., 2019). In parallel, Indigenous Peoples’ and local communities’ roles and rights are increasingly acknowledged in conservation policy and discourse (Artelle et al., 2019). Scaling out community area‐based conservation, rather than strict protections, is promoted as an inclusive, socially just, and durable approach to meeting 30×30 targets, though this outcome is not guaranteed (Adams & Hulme, 2001; Dawson et al., 2024; Dressler et al., 2010; Magessa et al., 2020; Oldekop et al., 2019; Ribot et al., 2010).

Scaling out means adapting and reproducing an initiative in new locations and communities (Moore et al., 2015) (see “Conceptual Framework”). Results shaped by research from other sectors (e.g., Hartmann & Linn, 2008; Rogers, 2003; Wigboldus & Brouwers, 2016; Wigboldus et al., 2016; Woltering et al., 2019) provide valuable insights for conservation practice (e.g., Jagadish et al., 2021; Salafsky & Margoluis, 2021). For instance, Mills et al. (2025) explored the drivers of adoption of 5 community‐based conservation initiatives through expert interviews. Initiatives offering economic and social benefits that aligned with local needs and were supported by adequate external facilitation were considered most likely to be widely adopted.

Recent advancements in other fields emphasize identifying and managing barriers to adoption as a pragmatic approach for accelerating scaling (McLean & Gargani, 2019). For example, Sartas et al.’s (2020b) Scaling Readiness decision‐support process for agricultural innovations includes diagnosing context‐specific scaling bottlenecks (factors limiting the adoption of initiatives). Identifying limiting factors to scaling can inform strategies to overcome them (Sartas et al., 2020a).

Although scarce funding is a barrier to scaling in conservation (e.g., Senior et al., 2024; Waldron et al., 2013), few researchers have examined other potential bottlenecks. Lewis–Brown et al. (2021) is an exception; they used a best‐to‐worst scaling experiment to identify community conflict and resource scarcity as the main barriers to locally managed marine areas adoption. Others identified the absence of suitable business models as a barrier to scaling up nature‐based solutions (Favero & Hinkel, 2024; Sánchez‐Arcilla et al., 2022). Nevertheless, to our knowledge, no one has evaluated scaling bottlenecks across community‐based conservation initiatives at national scales.

We aimed to integrate expert insights with existing theory and evidence to identify potential bottlenecks for scaling community area‐based conservation. We examined 10 initiatives in southern and eastern Africa (Table 1). Ultimately, we sought to identify bottlenecks that could be addressed to help scale socially just and durable conservation approaches.

**TABLE 1:** Case study initiatives included in the study of scaling bottlenecks in community conservation in southern and eastern Africa.

Country	Initiative name	Key legislation	Summary	Description of adoption^a^
Kenya	Community Conservancy	Kenyan Wildlife Conservation and Management Act (2013) & Community Land Act (2016)	“Land designated by a community […] for purposes of wildlife conservation and other compatible land uses” (KWCA, 2016)	Participating residents have registered as a legal entity (Community‐Based Organization, Trust, or Company) to operate a Community Conservancy. This can include legal entities that have not yet registered with the Kenyan Wildlife Service or applied for Wildlife Users Rights.
Kenya	Participatory Forest Management	Forests Act (2005) & Forest Conservation and Management Act 2016	forest management approach that deliberately involves forest‐adjacent communities and other stakeholders in the management of forests within a framework that contributes to the community's livelihoods (Government of Kenya, 2015)	Participating residents have formed a community forest association and been granted permission by the Chief Conservator of Forest Service to participate in the management of a state or local authority forest.
Malawi	Village Forest Area	Forestry Act (1997), National Forest Policy (2016) & Forestry Amendment (2017, published in 2020)	arrangement that permits community management of customary forest areas for the benefit of communities	Participating residents have established a Village Natural Resource Management Committee and signed a Forest Management Agreement with the Director of Forestry.^b^
Malawi	Comanagement of Forest Reserves	Forestry Act (1997), National Forest Policy (2016) & Forestry Amendment (2017, published in 2020)	arrangement between the government and a community allowing managed access to forests	Local groups have entered into agreements with the Director of Forestry to implement a mutually agreed management plan.
Tanzania	Community‐based Forest Management	National Forestry Policy (1998), Forest Act No 14 (2002) & Forest Regulations of 2019, Government Notice Number 417 (2019)	participatory forest management “approach that takes place on village land, on forests that are owned or managed by the Village Council on behalf of the Village Assembly and leads to the establishment of Village Land Forest Reserves (VLFR), Community Forest Reserves (CFR) or Private Forest Reserves (PFR)” (Ministry of Natural Resources & Tourism Forestry & Beekeeping Division, 2007; URT, 2022).	Village Land Forest Reserve has been declared by the District Council (following an application by an elected Village Natural Resource Committee). This includes Village Land Forest Reserves that have not yet been gazetted.
Tanzania	Wildlife Management Areas	Wildlife Policy of Tanzania (1998, 2007), Wildlife Conservation Act No. 5 (2009), Wildlife Conservation Act [CAP. 283 R.E. 2022] (2022) & Wildlife Conservation (Wildlife Management Areas) Regulation (2018)	arrangement allowing “rural communities […] to manage wildlife on their land for their own benefit” and “devolving management responsibility of the settled and areas outside unsettled PAs (protected areas) to rural people” (URT, 1998, 2007; USAID, 2013)	Participating residents have registered as a community‐based organization and the director of wildlife has formally gazetted the [wildlife management areas].
Uganda	Collaborative Forest Management	National Forestry and Tree Planting Act (2003)	arrangement to establish “a mutually agreed upon and beneficial relationship between an eligible local community group and the governing authority of either a Central Forest Reserve […] or a Local Forest Reserve […]” (Kazoora et al., 2020)	Representatives of local groups have formally signed Collaborative Forest Management agreements with the National Forestry Authority or local authority (responsible bodies). This includes local groups who previously signed agreements that have now expired. It does not include the number who have lodged applications but have yet to sign agreements.
Uganda	Community Wildlife Management Areas	Uganda Wildlife Act (2019)	arrangement establishing an “area in which individuals who have property rights in land may carry out activities for the sustainable management and utilisation of wildlife if the activities do not adversely affect wildlife” (Government of Uganda, 2019)	Participating residents have developed and submitted a management plan to the Uganda Wildlife Authority, which has been approved.
Zambia	Game Management Areas	Wildlife Act No. 14 (2015) & Zambia Wildlife Act No. 12 (1998)	“Semi‐protected game management areas (GMAs) which are multiple‐use zones which typically buffer the national parks” (Lyons, 2000); Community Resource Boards comanage wildlife in Game Management Areas with the Zambia Wildlife Authority (Davis et al., 2020).	Residents have elected Village Action Groups (who have then elected Community Resource Boards) and have actively participated in wildlife management.
Zambia	Community Forest Management Areas	Forests Act (2015)	arrangement between the government and a community group “to transfer authority to control access, use and management of a designated forest area” (Government of Zambia, 2018)	Participating residents have elected a Community Forest Management Group, and the Director of Forestry has approved the Community Forestry agreement.

^a^Definitions of *adoption* provided to experts during the first survey (see “Methods”). Adoption is the act of the adopter engaging in a new initiative (Jagadish et al., 2021; Rogers, 2003), although this does not imply that adopters have complete control in deciding whether to engage or not, that adoption on paper translates to action on the ground, or that scaling an initiative invariably leads to positive outcomes (Pienkowski et al., 2024).

^b^ This definition is contestable because many local groups establish de facto Village Forest Areas but do not sign formal Forest Management Agreements; forestry rules mean that the formal gazettement of Village Forest Areas allows the state to limit use rights and potentially appropriate forests.

## CONCEPTUAL FRAMEWORK

We drew on adoption, diffusion, and scaling scholarship. Adoption is the process through which individuals or groups engage with an initiative by, for example, signing an agreement, implementing an action, or changing behavior (Jagadish et al., 2021; Rogers, 2003). Adoption is often an ambiguous, dynamic process in which the intensity of engagement can fluctuate over time (Leeuwis & Aarts, 2021; Montes de Oca Munguia et al., 2021). Diffusion is the process by which an initiative is communicated through different channels over time among the members of a social system (Rogers, 2003).

Moore et al. (2015) distinguish between scaling out, scaling up, and scaling deep. Scaling out, our primary focus, involves expanding an initiative to reach additional people or locations, typically by adapting initiatives to new contexts. Scaling up refers to influencing institutional structures to alter rules and incentives, thereby facilitating adoption (Hartmann & Linn, 2008; Lam et al., 2020; Lambin et al., 2020). Scaling deep involves shifting underlying values, social norms, and knowledge to foster adoption (Moore et al., 2015). Scaling out is an emergent outcome of individuals and groups deciding whether to adopt an initiative that is shaped by complex interactions among biophysical, social, economic, and institutional factors (Geels, 2011; Leeuwis & Aarts, 2021; Wigboldus et al., 2016). Barriers to adoption at the individual or group level may represent major scaling bottlenecks, especially if experiences with these barriers are communicated (i.e., diffused) or cut off subsequent diffusion to other potential adopters.

Many public health, agricultural, development, and environmental interventions focus on groups, requiring collective decision‐making and buy‐in (McLean & Gargani, 2019). Therefore, we situated adoption in a wider framework of community conservation. Abernethy et al. (2014) identified 5 steps in the adoption of community‐based marine resource management: agenda setting (recognition of problems that need fixing); matching (deciding whether community management aligns with the problem); redefining and restructuring (determining management rules and governance structures); clarifying (putting rules and governance structures into use); and “routinizing” (normalizing rules and governance). However, this framework does not include the role of external support, which can be instrumental in adoption (e.g., Jagadish et al., 2024; Jørgensen et al., 2024; Mascia & Mills, 2018; Pienkowski et al., 2025; Romero‐de‐Diego et al., 2021; Battista et al., 2017). We identified 3 broad stages of adoption to loosely structure our conceptual framework: awareness and understanding, motivation, and support. Local groups gain awareness and understanding of initiatives via word of mouth, social media, or awareness‐raising by conservation organizations. Once aware, local groups are motivated to adopt initiatives. This includes decision‐makers evaluating the relative costs and benefits of adoption, how they are distributed, and whether they are aligned with local needs. Aware and motivated local groups receive the necessary support to adopt initiatives, including technical and financial assistance.

We expected there to be multiple potential bottlenecks in each of these 3 broad stages. Therefore, we drew on diffusion of innovations theory (study of how, why, and at what rate innovations spread among people, groups, organizations, or countries) (Rogers, 2003). Building on this literature, Jagadish et al. (2021) offer a framework for identifying attributes related to the conservation initiatives, potential adopters, and the socioecological contexts that may influence adoption. Barriers to adoption related to these attributes at the group level may represent major scaling bottlenecks. Therefore, we clustered these themes into our 3 stages through iterative discussions among us (Table 2).

**TABLE 2:** Potential bottlenecks to adoption of 10 community conservation initiatives in southern and eastern Africa grouped by stages of awareness, motivation, and support to adopt initiatives.

Potential bottleneck	Definition	Rationale	Survey question description
**Awareness and** **understanding**			
Awareness	“degree to which [a population of potential adopters is] familiar with the initiative and its consequences through existing knowledge and skill set or acquisition of new skills and knowledge” (Jagadish et al., 2021)	Communities that are unaware of initiatives are unlikely to adopt (Jagadish et al., 2021; Wejnert, 2002).	One question about the proportion of rural populations expected to be familiar with the initiative
Complexity	“degree to which the initiative is perceived as relatively difficult to understand and use” (Jagadish et al., 2021)	Adoption of initiatives is less likely when they are difficult to understand because of their complexity (Jagadish et al., 2021; Rogers, 2003).	One question about how easy it is to understand the initiative
Observability	“degree to which the initiative and the results of that practice are visible (observable or communicated) to others” (Jagadish et al., 2021)	Adoption of initiatives is less likely when the benefits of adoption are not clearly observable to potential adopters (Jagadish et al., 2021; Rogers, 2003).	One question about the visibility of benefits to other local groups
**Motivation**			
Relative advantage	“degree to which an initiative is perceived as being better than the idea, practice, or object that precedes it” (Jagadish et al., 2021)	Adoption of initiatives is less likely when the net costs of adoption outweigh the net benefits (Jagadish et al., 2021; Rogers, 2003). Aspects of relative advantage should be considered in the context of the other motivation‐related themes.	set of 8 questions on the effects of adoption on: adopter incomes, adopter access to natural resources, adopter access to crop farmland, adopter access to livestock farmland for consumption, adopter access to infrastructure, conflicts between local people, effects on local traditions and cultures, control of natural resources plus 1 question on overall impacts of local lives (Brooks et al., 2013; Mahajan et al., 2021; McKinnon et al., 2016; Roe et al., 2009)
Fair distribution of costs and benefits	degree to which the benefits and costs of adopting an initiative are shared fairly; fairness can include distributing benefits to those most in need, who invest the most resources into the initiative, or who face the greatest costs from the initiative (Gurney et al., 2021)	Communities may be less likely to act collectively or more actively oppose initiatives that result in unfair cost and benefit distributions (Mahajan et al., 2021).	Two questions on how fairly the costs and benefits of adoption were distributed within local adopter groups (intragroup benefit sharing) and with nonadopters (intergroup benefit sharing) Gurney et al. (2021)
Equal decision‐making	“Decision‐making arrangements specify the rights of individuals or groups to make choices regarding various aspects of conservation initiative design and management” (Jagadish et al., 2021)	Equal decision‐making may indicate the extent to which all members of local groups can influence the decision to adopt. Highly unequal decision‐making may increase the risks of elite capture (e.g., Persha & Andersson, 2014), making the adoption of initiatives less attractive.	One question about the balance of decision‐making power within local groups
Compatibility with local needs	“degree to which the initiative is perceived as consistent with existing values, existing actions, past experiences, and needs of potential adopters” (Jagadish et al., 2021)	Adoption of initiatives is less likely when they do not align with local needs (Cox et al., 2010; Jagadish et al., 2021; Mahajan et al., 2021; Rogers, 2003).	One question about the initiative's compatibility with the most important needs of local groups
Flexibility	“ability to transform the initiative to something that aligns with the adopter desires and constraints” (Jagadish et al., 2021)	Adoption of initiatives is less likely when they cannot be tailored to local interests and constraints (Greenhalgh et al., 2004).	One question about how easy it is to adapt the initiative to meet local needs
Geographical suitability	“Settings that affect adoption by influencing how well the initiative fits the adopter's socioecological context (e.g., initiatives that can only be established in certain habitats)” (Jagadish et al., 2021)	Initiatives with limited geographical suitability (e.g., requiring extremely specific socioecological conditions) are less likely to be widely adopted (Jagadish et al., 2021).	Two questions on the types of areas where the initiative can be established (e.g., in or bordering protected areas, in specific habitats) and the percentage of each country that these areas encompass
Land and resource rights recognition	formal recognition and support of Indigenous peoples and local communities’ rights to govern and conserve their collective lands, waters, and territories (ICCA Consortium, 2021)	Local communities with weak land and natural resource rights are less likely to act collectively or invest effort and resources (Baynes et al., 2015; Cox et al., 2010; Mahajan et al., 2021).	Two questions about the strength of recognition and respect of communal land and resource rights
**Support**			
Supportive national policies	“degree to which government policies, political structure, and political character change the relative advantage of the initiative or the ability to implement it” (Jagadish et al., 2021)	Adoption of initiatives is less likely when national policies present bureaucratic or other barriers to adoption (Jagadish et al., 2021).	One question on the extent to which national policies support local groups to adopt initiatives
Extension support	Extension support includes “public and private sector activities relating to technology transfer, education, attitude change, human resource development, and sharing of information which influences adoption and implementation of the initiative” (Agrawal & Gibson, 1999; Jagadish et al., 2021).	Adoption of initiatives is less likely when inadequate technical or financial assistance (Jagadish et al., 2021).	Two questions about the amount of technical assistance from external organizations^*^ and the availability of financial support to help local groups adopt

* Including government agencies, national and international nongovernmental organizations, businesses, and funding bodies.

Scaling out an initiative does not necessarily ensure positive social and ecological outcomes (McLean & Gargani, 2019; Pienkowski et al., 2024). Community area‐based conservation initiatives have sometimes been implemented because of top‐down pressures rather than local groups’ active choice to adopt (e.g., Benjaminsen et al., 2013; Bluwstein & Lund, 2018; Green & Adams, 2015; Lund & Bluwstein, 2018; Lund et al., 2017). Moreover, pressures to scale an initiative can incentivize unethical and coercive practices that undermine long‐term outcomes for people and nature (Pienkowski et al., 2024). These practices may represent scaling bottlenecks, especially when negative experiences with initiatives and their implementation diffuse among potential future adopters. Moreover, they can indicate socially inequitable or unsustainable approaches to achieving scaling that should be avoided. We asked experts to characterize decision‐making dynamics between external organizations and local groups (Table 3).

**TABLE 3:** Key attributes characterizing decision‐making dynamics between external organizations and local groups in 10 community conservation initiatives in southern and eastern Africa.

Attribute	Definition	Rationale	Survey question description
Control of adoption	degree to which local groups or external organizations^*^ have control over whether local groups adopt.	There may be an increased risk of detrimental impacts on local groups where they have limited control over adoption.	One question on whether local groups or external organizations had the greatest control over when local groups adopted initiatives.
Leadership of the adoption process	degree to which the adoption process is driven from the bottom‐up (e.g., local groups approach external organizations for support) or top‐down (e.g., external organizations^*^ identify and approach local groups).	“Organic” adoption (i.e., adoption that occurs without the need for external assistance) is less likely when external organizations play leading roles in the adoption process (Salafsky et al., 2021).	One question on whether local groups or external organizations led the process of local groups engaging with initiatives

^*^ Including government agencies, national and international nongovernmental organizations, businesses, and funding bodies.

## METHODS

### Overview

Our study protocol had 4 main phases: identification and recruitment of suitable experts and development of the survey instruments based on prior evidence and theory (see “Conceptual Framework”); deployment of an internet survey to 10 expert groups (1 group per initiative) to identify potential scaling risk factors; expert completion of a second survey that presented all potential risk factors and asked whether these factors represented scaling bottlenecks and why; and experts’ discussion of findings and finding implications via email and video conferencing. We distinguished between potential risk factors and scaling bottlenecks based on the results of the 2 surveys. Potential risk factors were defined as characteristics of an initiative or its socioecological context that, based on prior theory and evidence (Table 2), may act as barriers to scaling initiatives in general. In contrast, scaling bottlenecks were potential risk factors that experts confirmed to be barriers to scaling specific initiatives. The 2‐step elicitation phase separated the task of characterizing the initiatives (first survey) and then evaluating whether those characteristics represented bottlenecks (second survey) to reduce the cognitive burden.

The Imperial College Research Ethics Committee approved the study protocol (reference number 6406620). The ethical protocol included obtaining free, prior, and informed consent from all experts and protecting their personal information and the anonymity of their responses. Experts who completed the first elicitation phase were invited to help interpret results, identify implications and limitations, and conceptualize, write, and review the manuscript. Code and anonymized data are available at https://doi.org/10.5281/zenodo.16793566.

### Case study initiatives

Our case study initiatives included community area‐based conservation initiatives formally recognized in Uganda, Malawi, Tanzania, Kenya, and Zambia (Table 1). The lead research team (T.P., A.J., and M.M.) purposefully sampled these study countries to capture a range of terrestrial ecoregions (Olson et al., 2001). The lead research team then reviewed gray literature (e.g., Roe et al., 2009) and peer‐reviewed publications (Baynes et al., 2015; Blaikie, 2006) to identify candidate initiatives and determine which to include based on criteria in Appendix S1.

### Expert elicitation protocol

Expert judgments are often used to guide decision‐making related to complex problems in data‐limited settings (Dias et al., 2018). A wide range of factors can shape experts’ judgments, including cognitive availability (i.e., “judgments…influenced more heavily by… experiences or evidence that most easily come to mind”), confirmation (i.e., “people search for or interpret information […] in a way that accords with their prior beliefs”), and framing (i.e., “different conclusions from the same information, depending on how that information is presented”) biases (McBride et al., 2012; Martin et al., 2012). Expert judgments can also be shaped by motivational factors, including personal values, beliefs, and conflicts of interest. For example, experts directly involved in implementing initiatives might have financial or career‐related incentives to provide more favorable accounts. Similarly, experts in technocratic positions who are distanced from on‐the‐ground realities might be less aware of issues associated with initiatives. However, expert perceptions—often informed by experiences across multiple sites and contexts—can provide valuable insights into the social and ecological outcomes, legitimacy, and local acceptance of conservation measures (Bennett, 2016). In our study, areas with high expert consensus likely represent potential bottlenecks worthy of further attention and response, but may not be comprehensive.

In the pre‐elicitation phase, we identified and recruited suitable expert participants. The lead research team identified 30 conservation researchers and practitioners with demonstrable conservation knowledge and extensive professional networks in each country (termed panel members) (Table 4 & Appendix S4). The lead research team evaluated these characteristics based on online profiles and gray and peer‐reviewed literature and invited 16 to collaborate via email. Collaborating panel members were asked to review the list of case study initiatives to ensure it was comprehensive.

**TABLE 4:** Selection criteria for panel members and experts on scaling bottlenecks in 10 community conservation initiatives in southern and eastern Africa.

Panel members	Experts
English speaking Demonstrable knowledge of conservation projects in a target country (could be demonstrated through participation in high‐level conservation initiatives and activities or having written in gray and peer‐reviewed literature) Expected to have extensive professional networks in conservation in the target country; networks should span field‐based to high‐level decision‐making roles that could be demonstrated by being in positions requiring extensive networks or having substantial experience working in conservation in the target country Preference given to African professionals, who are more likely to have relevant experience and networks	English speaking Demonstrable knowledge of the target initiative (could include being involved in the delivery, evaluation, or research related to the target initiative) Extent to which they add diversity (in terms of career stage, gender, and job role) to the expert group Extent to which they add diversity in terms of expert group types (academia or research, community umbrella organizations, government, local or national NGOs, international NGOs, and business and the private sector) Preference given to African professionals, who are expected to have more relevant expertise

The lead research team then asked the panel members to identify at least 6 experts on case study initiatives in their country. Panel members completed an Excel document in which they assessed potential experts relative to the selection criteria (Table 4) and provided their names and contact details (Appendix S5). Panel members were asked to select at least 1 potential expert from 6 expert group types: academia or research, community umbrella organizations, government, local or national nongovernmental organizations (NGOs), international NGOs, and business and the private sector.

Panel members identified 216 potential experts. The lead research team emailed them with information about the study and an invitation to collaborate. We aimed to engage with at least 6 experts for each initiative (Hemming et al., 2018). Initially, we did not meet this minimum number for 2 initiatives. Therefore, we asked participating experts to suggest additional experts; thus, we identified 26 more experts.

In the first elicitation phase, experts were provided with guidance for developing high‐quality judgments, including that experts must not speak to each other when responding. Experts provided judgments via an internet Qualtrics survey (Appendix S6), which included questions adapted from Jagadish et al. (2021) grouped into the stages of awareness, motivation, and support (Table 2, see “Conceptual Framework”). Most of these questions were Likert‐scaled. This survey sought to identify characteristics of initiatives that represented potential risk factors and was conducted from June 2023 to March 2024.

Following data collection, we selected factors for which the majority (≥50%) of responses in an expert group indicated it was a potential scaling risk factor (typically, the negative response options in Likert‐scaled questions focused on attributes deemed to potentially influence scaling [Jagadish et al., 2021]). For example, 1 question asked how the initiative affected participating local groups’ incomes. If more than ≥50% of experts said it led to either a small reduction in income or a large reduction in income (i.e., negative responses), then this was considered a potential risk factor based on prior theory (Table 2).

In a second internet Qualtrics survey, experts were asked to evaluate whether identified potential risk factors represented scaling bottlenecks and, if they did, why (Appendix S7). We expected that not all potential risk factors would be considered scaling bottlenecks. For example, increased benefits in other areas might offset reduced incomes, so reduced incomes alone may not represent a bottleneck. Therefore, in the second survey, experts were shown the identified potential risk factors and asked whether they represented scaling bottlenecks and why or why not. We report on factors for which the majority (≥50%) of experts believed it was a scaling bottleneck (see “Study Limitations”). The second elicitation phase was conducted from August 2024 to October 2024.

We were also interested in whether there was variation in responses between expert group types. We fitted a series of cumulative‐logit mixed‐effects models with expert group type as the predictor and Likert‐scale responses as ordinal outcomes (Appendix S8). Additionally, given the narrowly defined scope of the open‐ended questions and the limited number of responses, extensive thematic analysis of latent meanings would have yielded minimal additional insight. Thus, we adopted a direct semantic interpretation, reporting the number of experts we considered to have provided a given explanation, supported by illustrative quotes.

## RESULTS

A total of 242 experts were contacted, of which 84 (34.7%) completed the first survey. The experts were affiliated with academia or research institutions (26, 31%), international NGOs (20, 23.8%), government agencies (14, 16.7%), local or national NGOs (12, 14.3%), community umbrella organizations (4, 4.8%), businesses or private companies (2, 2.4%), or other roles, primarily independent consultants (6, 7.1%). Sixty‐eight experts (81%) had over 10 years of experience in community‐based conservation, 14 (16.7%) had 5−10 years of experience, and 2 (2.4%) had <5 years of experience. Twenty‐five experts responded to the second survey to identify scaling bottlenecks (see “Study Limitations”).

There was a large variation in the number of potential risk factors and scaling bottlenecks identified for each initiative (Table 5). For instance, over 10 potential risk factors were identified among Village Forest Areas in Malawi, Community Wildlife Management Areas in Uganda, and Game Management Areas in Zambia. In contrast, only 3 potential risk factors were identified among Community Conservancies in Kenya and Community Forest Management Areas in Zambia.

**TABLE 5:** Characteristics assigned by experts to 10 community area‐based conservation initiatives (first survey) that could be bottlenecks to scaling^a^ (+) and scaling bottlenecks confirmed by experts in a second survey^b^ (++).

	Kenya		Malawi		Tanzania		Uganda		Zambia			
Characteristic	CC (10, 6)^c^	PFM (8, 1)	CMF (6, 1)^c^	VFA (6, 1)	CBFM (15, 6)	WMA (7, 1)^c^	CFM (11, 3)^c^	CWMA (6, 4)	CFMA (9, 1)^c^	GMA (6, 1);^c^	Risk factor count	Bottleneck count
Awareness and understanding												
Complexity Δ						+		++		++	3	2
Observability				++		+		++			3	2
Awareness^d^							++				1	1
Motivation: relative advantage												
Adopter access to crop farmland	++			+	++	+		++		+	6	3
Adopter access to natural resources	++			+	++	+				+	5	2
Conflicts between local people			+	+		+			+	++	5	1
Adopter access to livestock farmland					++	++		++		+	4	3
Adopter access to infrastructure										+	1	0
Control of natural resources										+	1	0
Adopter incomes											0	0
Effects on local traditions and cultures											0	0
Overall impacts on local lives											0	0
Motivation: other factors												
Equal decision‐making Δ	++	+	++	++		+	+	++		++	8	5
Intergroup benefit sharing Δ		+		++	++		+	++	++	+	7	4
Flexibility Δ			+			+	++	++		++	5	3
Communal land rights recognition			+	++			+		++		4	2
Intragroup benefit sharing Δ		+					++			+	3	1
Compatibility with local needs			++	++							2	2
Geographical suitability^e^												
Communal resource rights recognition											0	0
Support												
Financial assistance		++	++	+				++		++	5	4
Technical assistance		++		++							2	2
Supportive national policies											0	0
Decision‐making between external organizations and local groups												
Leadership of the adoption process Δ				++			++	++		++	4	4
Control of adoption Δ							++	++			2	2

^a^ Greater than or equal to 50% of experts gave responses that we considered indicative of potential risk factors in the first survey.

^b^ Greater than or equal to 50% of experts believed a given risk factor, which we identified from the first survey, was confirmed as a scaling bottleneck in the second survey.

^c^ All factors included in the first survey are shown, and the first number in parentheses is the count of expert responses in the first survey, and the second number is the count in the second survey. Abbreviations: CC, Community Conservancy; PFM, Participatory Forest Management; CMF, Comanagement of Forest Reserves; VFA, Village Forest Area; CBFM, Community‐based Forest Management; WMA, Wildlife Management Areas; CFM, Collaborative Forest Management; CWMA, Community Wildlife Management Areas; CFMA, Community Forest Management Areas; GMA, Game Management Areas; Δ, variability between expert groups (at 95% credibility interval). Rows are in descending order of the number of potential risk factors across all initiatives in each stage of adoption and the decision‐making dynamics theme.

^d^ Awareness was considered a potential risk factor when ≤ 30% of the rural public were estimated (on average across experts) to be aware of the initiative.

^e^ Geographical suitability was considered a potential risk factor when ≥50% of experts believed that < 20% of a country's geographical area was suitable for establishing a given initiative.

### Awareness and understanding

Awareness, complexity, and observability were not identified as major bottlenecks for most initiatives (Table 5 & Appendix S9). Public awareness, initiative complexity, and observability were identified as potential risk factors for less than one‐third of initiatives (although where this occurred, they were considered potential bottlenecks in most instances). There was credible variability among expert group types in their assessments of initiative complexity. Specifically, government representatives (predicted probability [PP] = 0.1, 95% credibility interval [CI] 0.02−0.24) (Appendix S10) were less likely to report that an initiative was difficult or very difficult for local groups to understand than those in academia or research (PP = 0.54, 95% CI 0.34−0.74), international NGOs (PP = 0.47, 95% CI 0.26−0.69), or local or national NGOs (PP = 0.33, 95% CI 0.12−0.58).

### Motivation

Experts were asked 8 questions to evaluate the relative advantages of engaging with initiatives (Table 5). Lost access to natural resources and crop farmland, and increased local conflict were identified as potential risk factors for at least half of the initiatives (Appendix S9), though only lost access to crop farmland was confirmed as a bottleneck in most of these cases. Eight experts who considered lost access to crop farmland a bottleneck indicated incompatibility between farming and conservation as a reason. Illustrating this, in the case of community conservancies in Kenya, 1 expert said:

Conservation and crop farmland are not compatible. Conservation and livestock farmland are compatible. Land use planning can help to plan for land that is possible to put under farmland.

In general, potential risk factors related to the relative advantage were identified for most initiatives (Appendix S9), but many of these were not considered scaling bottlenecks. The qualitative feedback from the second survey provided a general explanation for this. Three experts noted that individual costs or benefits should not be considered in isolation; instead, the trade‐off between them influences motivations to adopt. In the case of community conservancies in Kenya, 1 expert said: “It's too broad a question as it is entirely dependent on what other benefits are accrued through the association with the conservancy.”

This interpretation is supported by the result that most experts believed the initiatives improved local people's lives overall (Appendix S9). There was no statistically credible variability between expert group types in their assessments of the relative advantages or overall impacts of the initiatives on local people's lives (Appendix S10).

Experts were asked to evaluate how fairly the costs and benefits of each initiative were shared within participating local groups and with residents not directly engaged (Table 5). Unfair sharing of benefits and costs between participating and nonparticipating residents (i.e., intergroup benefit sharing) appeared to be a potential risk factor for 7 initiatives (Appendix S9) and was identified as a bottleneck among 4. Eight experts reported how experiences of unfair benefit sharing might undermine collective actions required for adoption. One expert on community‐based forest management in Tanzania said:

Because [non‐participating residents] are most likely bearing more cost than gaining benefits out of the initiative. Fairness could be improved by ensuring that even the non‐participants groups/local people benefit from [community‐based forest management]. That could be a better way to encourage them to participate and become allies as opposed to being adversaries of [community‐based forest management].

Two experts highlighted issues of elite capture in this feedback. For instance, 1 expert on community‐based forest management in Tanzania said: “In some cases, in the village there is power relation issues that lead into the elite capture, where the elite tend to benefit overall, leaving other member worse off in some cases.”

Relatedly, unequal decision‐making within local groups appeared to be a potential risk factor in most initiatives (Appendix S9) and was identified as a scaling bottleneck in the majority of these cases. An expert on the comanagement of forest reserves in Malawi provided 1 explanation for this:

If decisions are made by few and then imposed on the group, then it becomes a barrier as it limits a sense of ownership. Therefore, it is important to enhance leadership skills among the local groups, to ensure that major decisions are agreed on or validated by the larger community engaging in comanagement. For example, dates for participation of management activities such as fire break construction, if communities are not thoroughly consulted, they may not participate as this may conflict with other domestic or productive duties.

However, much of the feedback regarding local decision‐making was related to social equity. Commonly mentioned themes related to land tenure, gender, age, and elite capture (reported by 5 experts). For example, 1 expert on community conservancies in Kenya said:

Traditionally, there is a “big man” approach to leadership which can result in elite capture. More work needs to be done to promote diversity and inclusion while also being sensitive to cultural practices and not imposing outsider value systems.

Government representatives (PP = 0.17, 95% CI 0.04−0.4) (Appendix S10) were less likely to report that the benefits and costs were mostly or very unfairly shared within participating local groups than those in academia or research (PP = 0.47, 95% CI 0.25−0.71) or local or national NGOs (PP = 0.52, 95% CI 0.22−0.81). Government representatives (PP = 0.45, 95% CI 0.16−0.75) (Appendix S10) were also less likely to report mostly or very unfair benefit and cost distribution with nonparticipants compared with academics and researchers (PP = 0.77, 95% CI 0.55−0.92). Finally, government representatives (PP = 0.31, 95% CI 0.1−0.61) (Appendix S10) were also less likely to report fairly or very unequal decision‐making between members of local groups than those in academia or research (PP = 0.69, 95% CI 0.47−0.87) or local or national NGOs (PP = 0.69, 95% CI 0.41−0.9).

Among the other factors expected to influence motivations to adopt, lack of flexibility in rules and management activities was a potential risk factor among half the interventions (Appendix S9) and was confirmed as a bottleneck in most of these cases. Feedback from 5 experts contextualized this result, with 1 expert on community wildlife management areas in Uganda saying:

Enhancing the flexibility of rules and management in CWMAs is essential for aligning conservation goals with community needs. By adopting more inclusive, adaptive, and community‐centred approaches, these areas can achieve better conservation outcomes and foster greater community engagement and support.

However, 3 experts also highlighted the potential trade‐offs between local flexibility and ensuring that initiatives align with national conservation frameworks, such as 1 example from an expert on community‐based forest management in Tanzania:

Communities [should be] allowed to devise their own rules, especially in community‐based village land forest management. Often times the rules have to adhere to higher‐level rules of forest management which are centrally formulated with little consultation with the local governments. I would say again full participation is the answer, especially during central level rules making. In addition to that, customisation/localisation of the rules would be helpful.

Government representatives were less likely to say that initiatives were somewhat or very inflexible (PP = 0.19, 95% CI 0.06−0.41) (Appendix S10) than those in academia or research (PP = 0.42, 95% CI 0.21−0.64), local or national NGOs (PP = 0.43, 95% CI 0.19−0.72), and international NGOs (PP = 0.43, 95% CI 0.23−0.66).

### Support

Experts were asked about the wider conditions that might support local groups to adopt (Table 5). Inadequate financial assistance to support engagement was a potential risk factor for half of the initiatives (Appendix S9) and was identified as a bottleneck in most of these cases. Twelve experts provided written feedback supporting this. One of them, an expert on community wildlife management areas in Uganda, said:

Increasing the amount of financial assistance from external organisations is critical for the sustainability and success of Community Wildlife Management Areas (CWMAs). Financial resources are necessary to support the establishment, management, and development of these areas, covering costs such as infrastructure, conservation activities, capacity building, and community development projects. Securing external financial assistance can help communities manage CWMAs more effectively and engage more deeply in conservation efforts.

Five experts pointed to a shift from donor funding to more sustainable public and private sector sources. One expert on Community‐based Forest Management in Tanzania said:

The government should set a budget for that. The government should also engage other stakeholders including banks, and other financial institutions and NGO to also contribute towards [Community‐based Forest Management] as part of their [corporate] social responsibility in maintaining the forest cover around the country.

There was no statistically significant variability among expert group types in their assessment of the different dimensions of support (Appendix S10).

### Decision‐making between external organizations and local groups

Experts were asked a series of questions about decision‐making power between external organizations and local groups (Table 5 & Appendix S9). For 2 initiatives, most experts believed external organizations largely controlled whether local groups adopted initiatives or not. Similarly, for 4 initiatives, most experts believed the adoption process was top‐down (i.e., external organizations selected and approached local groups and led the adoption process). Among all these cases, these factors were considered scaling bottlenecks. One expert (echoing similar views of 8 others) on community‐based forest management in Tanzania said:

Because the approach is more top‐down than bottom‐up denying local community/groups an upper hand in making critical decisions that may affect their wellbeing. Local groups need to be adequately involved in the decision‐making process. Their views and ideas should always be taken into account.

Similarly, another expert on community wildlife management in Uganda said:

Increasing the leadership role of local groups in Community Wildlife Management (CWM) is crucial for fostering a sense of ownership, ensuring the sustainability of conservation efforts, and enhancing the effectiveness of wildlife management initiatives. When local groups lead, they are more likely to be invested in the outcomes, and the management practices are more likely to be culturally appropriate, contextually relevant, and sustainable over the long term.

Government representatives (PP = 0.15, 95% CI 0.04−0.35) (Appendix S10) were less likely to report that external organizations have most or almost complete control over adoption decisions than those in academia and research (PP = 0.42, 95% CI 0.2−0.66) and local or national NGOs (PP = 0.57, 95% CI 0.27−0.85). Similarly, government representatives (PP = 0.12, 95% CI 0.02−0.31) (Appendix S10) were also less likely to say that adoption is driven by external organizations than those in academia or research (PP = 0.48, 95% CI 0.24−0.72), local or national NGOs (PP = 0.64, 95% CI 0.32−0.9), and international NGOs (PP = 0.55, 95% CI 0.27−0.8).

## DISCUSSION

We identified consistent potential risk factors and scaling bottlenecks across 10 initiatives that may inform the scaling of socially just and durable conservation approaches to mitigate global biodiversity decline. These initiatives were community conservancies and participatory forest management in Kenya, village forest areas and comanagement of forest reserves in Malawi, community‐based forest management and wildlife management areas in Tanzania, collaborative forest management and community wildlife management areas in Uganda, and game management areas and community forest management areas in Zambia. Although most initiatives presented costs—such as lost access to natural resources and cropland and increased conflict—experts generally did not consider these costs major bottlenecks. Many experts believed adoption improved residents’ lives overall, emphasizing the need to assess complex trade‐offs rather than isolated costs and benefits. However, perceptions of unfair intragroup distribution of costs and benefits and unequal decision‐making were common risk factors and confirmed bottlenecks. A lack of flexibility in rules and management activities may also constrain scaling by limiting adaptation to local needs. Inadequate financial assistance and top‐down leadership were frequent bottlenecks, with the latter potentially undermining local ownership and organic adoption. Government representatives tended to have more favorable views of initiative complexity, fairness in sharing costs and benefits, decision‐making power, and control and leadership of the adoption process. Finally, the number of identified risk factors and bottlenecks varied widely across initiatives, suggesting unequal barriers to scaling.

### Scaling bottlenecks and the evidence base

Caution is needed when comparing our results with those in other studies. Many studies have evaluated the rules, implementation, and impacts of the 10 initiatives featured in our study, but they did not explicitly investigate whether these represent barriers to adoption. Moreover, many of these are case studies that provided nuanced contextual insights but did not provide nationally representative accounts with which our results can be directly compared. Nevertheless, comparison with prior evidence can help triangulate our results.

#### Relative advantages of adoption

Findings in several other studies align with our results on the relative costs and benefits of adopting initiatives. For example, previous studies highlight the reduction of local natural resource access among community conservancies in Kenya (e.g., Lesorogol & Lesorogol, 2024), community‐based forest management (e.g., Schreckenberg & Luttrell, 2009), and wildlife management areas (e.g., Igoe & Croucher, 2007) in Tanzania, and game management areas in Zambia (e.g., Milupi et al., 2020). Other studies highlight the trade‐offs between some community conservation areas and farming, aligning with our results. For example, Tyrrell et al. (2024) found that the Enonkishu Conservancy in the Maasai Mara in Kenya prevents the encroachment of farmland, partially consistent with our finding that community conservancies reduced access to crop farmland. Similarly, some studies illustrate how community area‐based measures can increase local conflicts (e.g., Larson et al., 2016), although carefully designed measures help mediate local tensions (Fariss et al., 2023). For example, aligned with our results, multiple studies describe how wildlife management areas have increased local conflict, such as over access to grazing and cropland and the impacts of wildlife, in parts of Tanzania (Bluwstein et al., 2016; Moyo, 2018; Moyo et al., 2016).

Results of other studies are not consistent with our findings. For example, evidence suggests that community conservancies in Kenya may have exacerbated local conflicts, such as over water resources (Greiner, 2012; Stephen et al., 2019). Yet, we did not identify conflict as a potential risk factor among community conservancies. Similarly, a national‐level study indicated that wildlife management areas in Tanzania have not delivered widespread poverty reductions (Keane et al., 2020). However, most experts in our study believed that wildlife management areas led to small income increases for local groups (Appendix S9). Similarly, 1 case study showed that comanagement of forest reserves in Malawi was associated with reduced access to natural resources (Chinangwa et al., 2016), but this was also not flagged as a potential risk factor in our study. One potential explanation is that most of these studies, except Keane et al. (2020), focused on localized case‐specific outcomes, contrasting with the nationally aggregated perspective reported by experts (but see “Study Limitations”).

More broadly, several experts stressed that initiatives often involve trade‐offs between costs and benefits, and the balance of these—not each in isolation—influences engagement. This result echoes that of Lewis–Brown et al. (2021), who found that decisions to engage with locally managed marine areas in north Madagascar were influenced by the balance of both perceived benefits (e.g., enhancing the well‐being of future generations) and costs (e.g., increased local conflict). Furthermore, across initiatives, most experts believed that initiatives generally improved the lives of local people. Therefore, our results might suggest that, overall, the relative advantages offered by initiatives may not be a major bottleneck to scaling (but see “Policy and Practice Implications”). Equally, individual elements of the relative advantage were seen as bottlenecks for some initiatives, so addressing these might support greater adoption.

#### Benefit sharing and local group decision‐making

Our finding that inequitable intragroup benefit sharing and unequal decision‐making are common potential scaling bottlenecks aligns with the characterization of many community conservation initiatives (e.g., Bluwstein et al., 2016; Bwalya Umar & Kapembwa, 2020; Kamoto et al., 2023; Moyo et al., 2016; Sulle & Banka, 2017). For example, Schreckenberg and Luttrell (2009) found that community‐based forest management in Tanzania and participatory forest management in Kenya increased incomes for committee members and elites, whereas poorer households experienced losses, like reduced access to natural resources. In their review of community forest management in Uganda, Kazoora et al. (2020) highlighted that women, youth, and other vulnerable groups tend to benefit less—partly because their interests and needs are not adequately considered in decision‐making—and that poor group governance can lead to intragroup disputes over benefits. It is widely recognized that communities comprise individuals and subgroups with differing interests and capabilities (Agrawal & Gibson, 1999) that shape participation and benefit distributions (Agrawal & Gupta, 2005). More broadly, negative experiences of adoption in 1 context might diffuse to, and subsequently inform the decision‐making of, potential adopters in other contexts, ultimately hindering scaling. In this context, it is perhaps unsurprising that inequitable intragroup benefit sharing and unequal decision‐making were common themes.

Yet, whether these factors actually hinder scaling is debatable. Some external organizations may seek to scale initiatives by engaging with local subgroups who stand to benefit (so support adoption) while overlooking those who may lose out (and thus might oppose) (Pienkowski et al., 2024). Such strategies might appear to accelerate the scaling of initiatives, even when there is general awareness of negative effects, but result in poor implementation or subsequent abandonment. Alternatively, local groups may be less likely to act collectively to adopt if they perceive unfair benefit sharing and unequal decision‐making elsewhere (Mahajan et al., 2021), thus limiting scaling. Although both dynamics might exist simultaneously, our results suggest unfair benefit sharing and unequal decision‐making may be scaling bottlenecks. Moreover, governments and NGOs should consider the ethical implications of promoting initiatives linked to these outcomes and how doing so might undermine the long‐term durability of conservation efforts (Agrawal, 2001; Pienkowski et al., 2024).

#### Flexibility and leadership of the adoption process

Inflexible rules and management activities were identified as a scaling bottleneck among 3 initiatives (community forest management and community wildlife management areas in Uganda and game management areas in Zambia), consistent with results from other studies (e.g., Bwalya Umar & Kapembwa, 2020). For instance, Zambia's game management area model has been criticized for its top‐down approach despite being classed as a collaborative governance arrangement (UNEP‐WCMC & IUCN, 2024). In communities near 2 game management areas, Milupi et al. (2020) found that two‐thirds of residents felt excluded from wildlife management consultations, and most were not involved in decision‐making. They concluded, “There is need for definitive legislation to be passed that delegate decision‐making about wildlife resources to the local communities in [game management areas].” Relatedly, many local groups in Malawi establish de facto village forest areas without formalizing Forest Management Agreements because doing so risks ceding rights and control of forests to the state. Ribot et al. (2010) argue that across Africa, decentralizing forest management has not yielded promised benefits due to insufficient devolution of power and institutional failure to represent local interests. Despite this finding, national policies were not identified as scaling bottlenecks for any initiative. One potential explanation is that there may be ambiguity in policies in how much control is devolved to local communities. Thus, the way initiatives are implemented—via interactions among local groups, government agencies, and other stakeholders—may have a greater impact on actual outcomes than how laws themselves are written (Agrawal & Gibson, 1999). Regardless of the reasons for inflexibility, our results support previous theories that suggest adoption of initiatives is less likely if they cannot be tailored to local interests and constraints (Greenhalgh et al., 2004; Jagadish et al., 2021). As above, the diffusion of information on this inflexibility between prior and potential adopters might hinder scaling.

Countless examples demonstrate how local groups can self‐organize to manage common natural resources under the right conditions (Ostrom, 1990). Nevertheless, external organizations can play essential roles in facilitating formalized community conservation initiatives (Abernethy et al., 2014; Agrawal & Gibson, 1999; Jagadish et al., 2024; Jørgensen et al., 2024; Mascia & Mills, 2018; Romero‐de‐Diego et al., 2021). However, top‐down approaches—where external organizations play an excessive role in leading the adoption process—may be concerning for 3 reasons. First, 1 scaling pathway in conservation is “organic” adoption—independent adoption by individuals and communities without external support (Salafsky & Margoluis, 2021). However, initiatives requiring significant technical or financial capacity are unlikely to be adopted organically, so scaling might be contingent on the availability of external support. Second, top‐down leadership might undermine the local group's ownership and control, thus eroding the buy‐in needed to deliver successful outcomes. A review of 170 studies showed that conservation initiatives with more equitable governance—characterized by greater local control and equal partnerships with external organizations—were associated with more positive social and ecological outcomes (Dawson et al., 2024). Hence, while in reality, most initiatives will scale via a mix of organic adoption and external facilitation (Clark et al., 2024), top‐down leadership might undermine social and ecological outcomes. Third, top‐down leadership may increase the risk of unethical practices (Pienkowski et al., 2024). For example, case studies demonstrate that the implementation of several wildlife management areas in Tanzania may have been driven by top‐down interests while being opposed by local actors (Benjaminsen et al., 2013; Bluwstein & Lund, 2018; Green & Adams, 2015), although experts in our study did not believe that external organizations played leading roles in driving the adoption of wildlife management areas in Tanzania. Therefore, initiatives characterized by top‐down leadership in adoption might raise red flags regarding equitable and durable scaling.

#### Financial assistance

Although conservation underfunding is widely recognized (e.g., Senior et al., 2024; Waldron et al., 2013), insufficient financial assistance was identified as a scaling bottleneck in only 4 initiatives. This result is perhaps surprising, given the widespread calls to increase conservation investment. One possible explanation for why inadequate financial assistance was not a more ubiquitous bottleneck is that experts believe that local groups receive sufficient support to engage (e.g., adequate financial support to convene community meetings or cover administrative costs) or that additional finance by itself will not drive more adoption. This leaves open the possibility that external organizations—often responsible for facilitating engagement—themselves remain underresourced (e.g., have insufficient funding to engage with more communities). This observation raises a broader point about our study's focus on local groups and the barriers they face. In reality, multiple actors operating at different societal levels play roles in scaling initiatives, and each may face distinct barriers. Future research might apply similar methods to identify scaling bottlenecks within this broader network of actors.

### Study limitations

Our study has several important limitations. First, our method did not involve speaking with residents directly involved in adopting initiatives. Furthermore, experts from community umbrella organizations and the private sector were underrepresented in our final sample. Therefore, our method did not present local actors’ perspectives (see “Policy and Practice Implications”). Second, only 30% of experts who participated in the first survey completed the second, and 6 initiatives had only 1 respondent. The reasons for this attrition were unclear; however, it may be attributed to time constraints and competing professional commitments faced by experts. Nevertheless, this attrition means that the scaling bottlenecks we identified reflect only the views of a subset of experts, so they should be treated cautiously. Third, experts were asked to provide nationally representative judgments (and some noted difficulties in doing this). However, there is likely a large amount of heterogeneity in the reasons local groups adopt an initiative in a given country, which was not captured by our method (see Policy and Practice Implications). Fourth, some experts might have cognitive or motivational biases, leading them to provide favorable accounts of initiatives (McBride et al., 2012). Our use of snowball sampling of experts for 2 initiatives might have increased this risk. Thus, the identified bottlenecks are likely not comprehensive, underscoring the need for broader research across case studies and methods to develop a holistic understanding of context‐dependent scaling barriers (see Appendix S11). Finally, we identified potential risk factors and bottlenecks based on the majority judgments among experts. However, many efforts to strengthen governance and equity focus on elevating the perspectives of marginalized or minority groups. Consequently, future attempts to identify scaling bottlenecks might not rely on majority perspectives, particularly for topics with substantial social justice implications.

### Policy and practice implications

Our results suggest that scaling bottlenecks may be closely tied to local governance and distributional issues, so holistic scaling strategies could involve enhancing environmental governance. Governance‐related bottlenecks in our study included unfair benefit sharing, unequal decision‐making, inflexibility, and top‐down leadership in the adoption process. In response, numerous tools and resources could be used to help strengthen local governance. For example, the Transformative Pathways project has produced a range of resources for supporting locally led, rights‐based environmental governance (e.g., Brittain et al., 2024a; Brittain et al., 2024b; Newing et al., 2024a). Similarly, the Site‐level Assessment of Governance and Equity tool helps identify governance challenges and plan actions to address them (Franks, 2023), and the Elinor tool supports monitoring area‐based conservation governance and management (Mahajan et al., 2024). Widespread scaling out of such tools and approaches could help address governance challenges, thereby potentially unblocking bottlenecks to scaling community area‐based conservation. Moreover, decentralized and effective environmental governance might better deliver conservation benefits (Bennett & Satterfield, 2018; Dawson et al., 2024). Therefore, scaling up (i.e., engaging with higher institutional levels) good governance might simultaneously support the scaling and effectiveness of community conservation.

Some of our results raise critical concerns about whether initiatives should be scaled further, redesigned, or even rolled back. Recognizing that poorly designed or executed conservation interventions can harm people or nature, Cavanagh and Brehony (2024) call for the use of “dark logic models” to anticipate potential failures based on prior experience. Such dark logic approaches could help forecast and mitigate social and ecological risks in scaling strategies. They should also consider the risk that scaling existing initiatives might stifle the development and implementation of alternative approaches, such as better recognition of Indigenous and traditional territories (Gurney et al., 2023).

Meaningfully consulting a diverse range of actors across contexts might lead to more effective nested scaling strategies. Actors more removed from on‐the‐ground realities and challenges may present more favorable assessments, potentially explaining the more positive responses given by government representatives. Consequently, a key caveat of our study is the absence of perspectives from local groups directly involved or affected by the initiatives, which might differ from our experts. Moreover, these local perspectives are likely highly heterogeneous between contexts. For example, adoption might help certain local groups legitimize land rights in 1 landscape while enabling others to secure resources in different landscapes (Berkes, 2004). In general, top‐down approaches of poor local consultations are likely to create conservation adversaries as opposed to conservation allies.

Thus, our approach could be adapted in several ways (see Appendix S12). The need for more participatory research and decision‐making is increasingly widely accepted, although underutilized, in conservation (Carrick et al., 2023; Newing et al., 2024b). These approaches are especially salient for community‐based conservation, where externally conceived interventions risk being imposed on local communities for conservation purposes (Khanyari et al., 2023; Rai et al., 2021). We believe that conservation ideas, practices, and models that deliver benefits in 1 context can prove useful in others, so the diffusion of these between suitable contexts can be valuable. Yet, imposing conservation models that are unwelcome or unsuitable, without adapting them to local priorities and constraints, can be harmful (Pienkowski et al., 2024). Therefore, better embedding participatory processes could help avoid this inappropriate imposition. However, these processes often require substantial investment of time and resources and can be counterproductive if not done well (Carrick et al., 2023; Newing et al., 2024b). This challenge may be mitigated through the development of nested scaling strategies that address nationwide constraints (e.g., through consultation with technical experts) alongside those unique to specific subregions and actor groups (e.g., through more participatory processes). Employing multiactor participatory processes to identify and address scaling bottlenecks aligns with contemporary scaling concepts. There has been a recent shift from viewing scaling merely as increasing adopter numbers toward fostering decentralized processes, emphasizing strategic partnerships, alliances, and knowledge sharing (Wigboldus & Brouwers, 2016).

We sought to integrate expert insights with existing theory and evidence to identify potential scaling bottlenecks among 10 community area conservation in southern and eastern Africa. Despite our study's limitations (e.g., not capturing local perspectives and expert attrition), governance and distributional issues were consistently highlighted as bottlenecks. Therefore, scaling strategies that strengthen environmental governance, potentially developed through inclusive consultation, may support more effective and equitable scaling of area‐based conservation toward global goals.

## Supporting information



Supporting Information
