# Supplementary material for: Diagnosing scaling bottlenecks in 10 community conservation initiatives in southern and eastern Africa
Source: Conserv Biol. 2025 Sep 12;40(2):e70149. doi: 10.1111/cobi.70149 (PMC13036310; doi:10.1111/cobi.70149)
Supplement: Supplementary file 1 — Supporting Information [file COBI-40-e70149-s001.docx]

Diagnosing scaling bottlenecks in ten community conservation initiatives in southern and eastern Africa

Appendix

Contents

[Appendix 1: Initiative inclusion criteria 2](#_Toc205808715)

[Appendix 2: Definition of community conservation 3](#_Toc205808716)

[Appendix 3: Original candidate list of initiatives 4](#_Toc205808717)

[Appendix 4: Panel member selection template 14](#_Toc205808718)

[Appendix 5: Expert selection template 16](#_Toc205808719)

[Appendix 6: Survey one template 18](#_Toc205808720)

[Appendix 7: Survey two template 32](#_Toc205808721)

[Appendix 8: Statistical analyses 60](#_Toc205808722)

[Appendix 9: Likert-scaled plots of results 61](#_Toc205808723)

[Appendix 10: Variation between experts 65](#_Toc205808724)

[Appendix 11: Techniques for more accurate expert elicitation 81](#_Toc205808725)

[Appendix 12: Multi-actor scaling bottlenecks 82](#_Toc205808726)

[References 83](#_Toc205808727)

## Appendix 1: Initiative inclusion criteria

1. Initiatives should meet the definition of community-based natural resource management or co-management initiatives (see *Appendix 2: Definition of community conservation*).
2. Initiatives should exist in geographically definable areas (CBD 2018).
3. Initiatives should be legally formalised nationally (i.e., established in government regulation or policy). Focusing on officially recognised initiatives helped us identify cases in a standardised way.
4. Initiatives must be terrestrial (i.e., excluding marine initiatives).

We initially identified five more initiatives than listed in Table 1. However, these were removed because a) one had little documented evidence that it existed in practice and b) four had near complete overlap in the list of potential experts (see *Appendix 3: Original candidate list of initiatives*). In the latter case, we selected the initiatives with more listed experts.

## Appendix 2: Definition of community conservation

Candidate initiatives must meet the definition of community-based natural resource management or co-management initiatives:

- Community-based natural resource management: “a process by which local groups or communities organise themselves with varying degrees of interaction with state agencies and outside support so as to apply their skills and knowledge to the care of natural resources while satisfying livelihood needs” (IPBES 2019)
- Co-management initiatives: “a participatory decision-making process in which the regulation of natural resource use is shared between the users and other stakeholders, such as the national or subnational government, [non-governmental organisations (NGOs)] and local cooperatives” (Freitas et al. 2020).

Additionally, the term “community” is not consistently defined between countries, and communities often lack clear social or geographical boundaries (Cleaver 1999; Blaikie 2006). Therefore, we instead refer to local groups as the units that adopt initiatives, which we define as “a collection of people living in the same place”, recognising these are fuzzy and poorly defined.

## Appendix 3: Original candidate list of initiatives

Table 1. The original list of candidate initiatives considered and why five were excluded.

| Country | Initiative name | The initiative meets the definition of a •Community-based natural resource management co-management initiative | The initiative exists in geographically definable areas (CBD 2018) and is terrestrial. | The initiative is legally formalised at a national level. | When was the initiative established? | Meets all eligibility criteria? | Exclusion? |
| --- | --- | --- | --- | --- | --- | --- | --- |
| Kenya | Community Conservancy | According to the Kenyan Wildlife Conservation and Management Act, 2013, “‘wildlife conservancy’ means land set aside by an individual landowner, body corporate, group of owners or a community for purposes of wildlife conservation in accordance with the provisions of this Act”.  The Kenyan Wildlife Conservation and Management Act, 2013 states, “Any person or community who own land on which wildlife inhabits may individually or collectively establish a wildlife conservancy or sanctuary in accordance with the provisions of this Act.” It goes on to say that “Communities, landowners, groups of landowners and existing representative organizations may establish a community wildlife association and register under the appropriate law or, in the case of an individual owner, may be registered as a recognized wildlife manager by the County Wildlife Conservation and Compensation Committee.” | Yes | Yes | 2013 | Yes | NA |
| Kenya | Group Conservancy | As above, according to the Kenyan Wildlife Conservation and Management Act, 2013, “‘wildlife conservancy’ means land set aside by an individual landowner, body corporate, group of owners or a community for purposes of wildlife conservation in accordance with the provisions of this Act”.  It is slightly uncertain if we should consider this intervention eligible. As mentioned, a Group Conservancy is “A single Conservancy created by the pooling of land by contiguous private land-owners for the purpose of wildlife conservation.” In this respect, these types of Conservancies can involves groups of individuals rather than communities per se. However, some of the other interventions (e.g., Tanzanian Community-based forest management) can also be established among groups of residents, rather than communities in their entirety. Therefore, we believe we should include this intervention. | Yes | Yes | 2013 | Yes | The majority of identified experts overlapped with Community Conservancies |
| Kenya | Participatory Forest Management | According to the Forests Act 2005, “An association registered under subsection (1) may apply to the Director for Permission to participate in the conservation and management of a state forest or local authority forest in accordance with the provisions of this Act.”  Furthermore, “An association approved by the Director under section 46 to participate in the management or conservation of a forest or part of a forest shall –   a) protect, conserve and manage such forest or part thereof pursuant to an approved management agreement entered into under this Act and the provisions of the management plan for the forest; b) formulate and implement forest programmes consistent with the traditional forest user rights of the community concerned in accordance with sustainable use criteria; c) protect sacred groves and protected trees; d) assist the Service in enforcing the provisions of this Act and any rules and regulations made pursuant thereto, in particular in relation to illegal harvesting of forest produce; e) with the approval of the Board enter into partnerships with other persons for the purposes of ensuring the efficient and sustainable conservation and management of forests; f) keep the Service informed of any developments, changes and occurrences within the forest which are critical for the conservation of biodiversity; g) help in fire fighting; and h) do any other that is necessary for the efficient conservation and management of the forest.”  According to the Forests Act 2005, “A member of a forest community may, together with other members or persons resident in the same area, register a community forest association under the Societies Act.” | Yes | Yes | 2005 | Yes | NA |
| Kenya | Co-Managed Conservancy | Unclear, as there is little formal documentation. | Yes | Unclear, as there is little formal documentation. | Unclear, as there is little formal documentation. | No (little formal documentation) | Inadequate formal documentation |
| Malawi | Village Forest Area | According to the Forestry Act (1997), a “‘village forest area’ means an area of customary land established such by an agreement under section 30;” These are managed by a “village natural resources management committee”, which means “a committee elected by stakeholders of the village forest areas.” The Forestry Act aims to “provide for promotion of participatory forestry on customary land through protection, control and management of trees and forests by the people on customary land, the demarcation and management of village forest areas, ownership of indigenous forest trees, establishment of tree nurseries and regulation of forest produce.” Furthermore, “Notwithstanding anything contained in this Act, any village headman may, with the advice of the Director of Forestry, demarcate on unallocated customary land a village forest area which shall be protected and managed in the prescribed manner for the benefit of that village community”.   However, the Act does not define “Communities”.  According to a 2001 Government of Malawi report (Government of Malawi 2001), community management of customary forests has two main objectives:  “- It aims firstly to stem the prevailing widespread destruction of forest resources on customary land and the material products and essential environmental services they provide. - The second and equally important objective is to encourage the development of customary forests and woodlands as important economic assets to the communities on whose land they occur.” | Yes | Yes | 1997 | Yes | NA |
| Malawi | Co-management of Forest Reserves | When describing the co-management of forest reserves, the Forestry Act 1997 states, “The Director of Forestry may enter into an agreement with local communities for implementation of the management plan that is mutually acceptable to both parties.”  According to a 2001 Government of Malawi report (Government of Malawi 2001), “Sections 44 and 46 [of the Act] specify other activities which are not expressly forbidden but may only be carried out within a Forest Reserve after obtaining a licence from the Director of Forestry. These include:  - depositing litter or noxious waste; - harvesting any forest produce; - cultivating crops and clearing or digging land; - grazing livestock; - prospecting for or extracting minerals, and - squatting, residing or erecting buildings, livestock enclosures or other structures.”  Furthermore, “Section 3(c) of the Act states that one of its purposes is to promote community involvement in the conservation of trees and forests in Forest Reserves. The management of a Forest Reserve must be conducted in accordance with a management plan drawn up by the Director of Forestry. But section 25 gives the Director the power to design and implement such a management plan in collaboration with the local communities...” | Yes | Yes | 1997 | Yes | NA |
| Malawi | Community Conservation Area | According to the National Parks and Wildlife Act 1994, a “‘community conservation area’ means a conservation area in respect of which authority and responsibility to manage and utilize certain wildlife resources therein is vested in the local community;” Additionally, “‘local community’ includes corporate and incorporate persons and non-governmental organizations;” and “‘wildlife management authority’ means any local community organization or other private organization established for the purposes of promoting local community participation in the conservation and management of wildlife.” The Act aims for “the promotion of local community participation and private sector involvement in conservation and management of wildlife; and”.  According to a USAID report (USAID 2010), “The consolidated Act of 1992 allows controlled harvesting of natural resources within the protected areas. The Act provided for community participation in the conservation and management of wildlife (section 22A) and it also allows the Director of National Parks and Wildlife to enter into wildlife management agreements with legally recognized wildlife management authorities (defined as any local community organization or other private organization established for promoting local community participation). The Act allows revenue sharing between the DNPW and communities through organized wildlife management authorities.” | Yes | Yes | 1994 | Yes | Panel members were unable to identify a sufficient number of experts |
| Tanzania | Community-based forest management | A 2019 report states that the “Forest Act of 2002, [...] introduced the concept of participatory forest management, consisting of managing reserves with local communities and granting concessions to communities living in the vicinity of forest reserves to manage forestland sustainably in agreement with the local forest administration” (FAO 2019).  A Government of Tanzania report states, “Participatory Forest Management (PFM) is a general term describing community involvement in the management of forests. Community-Based Forest Management (CBFM) is one kind of PFM approach that takes place on village land, on forests that are owned or managed by the Village Council on behalf of the Village Assembly and leads to the establishment of Village Land Forest Reserves (VLFR), Community Forest Reserves (CFR) or Private Forest Reserves (PFR)” (United Republic of Tanzania (URT) 2007).  “This arrangement is undertaken on village land that is surveyed and registered according to provisions in the Village Land Act of 1999” (FAO 2019). | Yes | Yes | 2002 | Yes | NA |
| Tanzania | Joint forest management | As above, an FAO 2019 report states that the “Forest Act of 2002, [...] introduced the concept of participatory forest management, consisting of managing reserves with local communities and granting concessions to communities living in the vicinity of forest reserves to manage forestland sustainably in agreement with the local forest administration” (FAO 2019).  A Government of Tanzania report (Joint Forest Management Guidelines) states, “Participatory Forest Management (PFM) is a general term describing community involvement in the management of forests. Joint Forest Management (JFM) is a form of PFM that takes place in forests on “reserved land” – land that has been set aside (or reserved) by government as part of either Local Authority or National Forest Reserves” (United Republic of Tanzania (URT) 2007).  “The forest is jointly managed by different stakeholders, such as local communities, private sector, local or central government or any body authorised by FBD” (United Republic of Tanzania (URT) 2007). | Yes | Yes | 2002 | Yes | The majority of identified experts overlapped with Community-based forest management |
| Tanzania | Wildlife Management Areas | The Wildlife Policy of Tanzania (1998, 2007) states, “Village communities living adjacent to protected areas, wetlands, or in wildlife corridors will be encouraged to establish Wildlife Management Areas (WMAs) in order to secure habitat for wildlife and halt wetlands degradation.”  The Wildlife Policy of Tanzania (1998, 2007) states, “Wildlife Management Area means an area set aside by Village Council for the purpose of conservation of wildlife and other biological natural resources, under the Wildlife Act”. | Yes | Yes | 1998 | Yes | NA |
| Uganda | Collaborative Forest Management | “Collaborative Forest Management aims to establish a mutually agreed upon and beneficial relationship between an eligible local community group and the governing authority of either a Central Forest Reserve (CFR) or a Local Forest Reserve (LFR) i.e., the “responsible body” (Kazoora et al. 2020).  The initiative involves an “eligible local community group” (Kazoora et al. 2020). | Yes | Yes | 2003 | Yes | NA |
| Uganda | Community Wildlife Management Areas | According to the Uganda Wildlife Act, 2019, a “community wildlife management area declared under subsection (3Xb) shall be an area in which individuals who have property rights in land may carry out activities for the sustainable management and utilisation of wildlife if the activities do not adversely affect wildlife and in which area the State may prescribe land use measures.” Furthermore, community wildlife management areas are one form of “wildlife management area”. Wildlife management areas can be used to:  “(a) to manage and control the use of land by people and communities living in the area so that it is possible for wildlife, the people and communities to coexist and for wildlife to be protected; (b) to enable wildlife to have full protection in wildlife sanctuaries not withstanding the continued use of the land in the area by people and communities ordinarily residing there; (c) to facilitate the sustainable exploitation of wildlife resources by and for the benefit of the people and communities living in the area; and (d) to permit the sustainable exploitation of the natural resources of the area, by mining and other like methods in a manner which is compatible with the continued presence in the area of wildlife.”  According to the act, “‘community’ means an assemblage of human beings living in a defined geographic area and identified by a common history, a common culture or a common residence in that area;” | Yes | Yes | 1996 | Yes | NA |
| Zambia | Game Management Areas | Lyons (2000) states, “semi-protected game management areas (GMAs) which are multiple-use zones which typically buffer the national parks. GMAs permit human settlement and low-impact land uses such as small scale agriculture and fishing, however large settlements and high-impact land uses such as mining or commercial forestry are prohibited. All wildlife in Zambia are owned by the state and administered by the Zambia Wildlife Authority”. ADMADE are established within GMAs (which exist outside protected areas which do not permit public use).   A joint report states, “In GMAs, the Wildlife Act provided for the establishment of Community Resource Boards (CRBs) as the institutions through which communities could work with ZAWA […] CRBs co-manage wildlife in GMAs with ZAWA and receive a share of revenues from trophy hunting. From that income, CRBs are required to recruit village scouts to assist ZAWA with law enforcement. These village scouts are appointed by ‘village action groups’ (3-10 of which are developed per chiefdom), which fall under CRBs” (Davis et al. 2020). | Yes | Yes | At least as early as 1998 | Yes | NA |
| Zambia | Joint Forest Management Area | The Forest Act, 2015, states, “‘joint forest management’ means the participation of stakeholders in the sustainable management of forest resources and the sharing of benefits derived from the management of the forest resources;”  According to the Forests Act, 2006, “‘Local community’ means residents within or adjacent to a declared Joint Forest Management Area, who by virtue of their rights over land, invest in and derived benefits from sustainable management and utilisation of forest resources in their area;”  The Forest Act, 2015, states, “The Director may, in consultation with a local community and with the approval of the Minister, constitute a joint forest management committee”. Furthermore, “the functions of a joint forest management committee shall be to manage and develop the joint forest management area and distribute the benefits amongst the local communities in the area”. | Yes | Yes | 2015 | Yes | The majority of identified experts overlapped with Community Forest Management Areas |
| Zambia | Community Forest Management Areas | According to The Forests (Community Forest Management) Regulations, 2018, “Subject to these Regulations, community forest management may be applied in— (a) open areas; (b)local Forests; and (c) Game Management Areas”. Furthermore, “A community forest management group shall consist of individuals who are citizens who reside in the community area and have resided in the area at least for 2 years”.  Furthermore, according to The Forests (Community Forest Management) Regulations, 2018, “The CFMG accepts and undertakes to protect, manage, control and utilize sustainably the forest resources in accordance with terms and conditions stipulated in this agreement and annexes attached thereof.” | Yes | Yes | 2015 | Yes | NA |

## Appendix 4: Panel member selection template

Table 2. The template that was used to evaluate candidate panel members against our selection criteria.

| Country  [Select from the drop-down list.] | Expert surname/last name/family name  [What is the expert’s family name?] | Expert first name/given name  [What is the expert’s given name?] | Expert title  [Select from the drop-down list.] | Expert email address  [What is the expert’s email address?] | Does the expert speak English?  [Select from the drop-down list.] | Demonstrable knowledge of conservation projects within a target country. This could be demonstrated through participation in high-level conservation initiatives and activities or having written (in grey and peer-reviewed literature).   Expected to have extensive professional networks in conservation within the target country. These networks should span field-based to high-level decision-making roles. This could be demonstrated by being in positions requiring extensive networks or having substantial experience working in conservation in the target country.   [Why are they chosen? Please provide a brief explanation of their relevant experience or expertise.] | Career stage (assumed or inferred)   [Based on publicly available information.] | Job role  [What is their job title or brief description.] | Organization  [Works in which organization.] | Found where? |
| --- | --- | --- | --- | --- | --- | --- | --- | --- | --- | --- |
| Uganda | Name | Fake | Dr | fn@expert.com | Yes | Dr Fake Name has extensive experience in research on rural livelihoods, particularly the dependence on local land resources. Her expertise includes agricultural development, the impacts of public policies on rural land and natural resource use, and natural resource management. | Mid-career | Associate Professor | Uganda University | Via Google Scholar |

## Appendix 5: Expert selection template

Table 3. The template that panel members were used to identify and evaluate candidate experts against our selection criteria.

| Country  [Select from the drop-down list.] | Expert surname/last name/family name  [What is the expert’s family name?] | Expert first name/given name  [What is the expert’s given name?] | Expert title  [Select from the drop-down list.] | Expert email address  [What is the expert’s email address?] | Does the expert speak English?  [Select from the drop-down list.] | Is the expert knowledgeable about the initiative? For example, have they been involved in implementation, policy, research and evaluation, or other activities related to the initiative? | Which best describes the expert? Are they an:  - Academic or researcher  - Representative of a community umbrella organisation  - Government representative  - Local or national NGO representative  - International NGO representative  - Business or private company representative  - Other (please describe) | Job role  [What is their job title or brief description.] | Organization  [Works in which organization.] | Other notes |
| --- | --- | --- | --- | --- | --- | --- | --- | --- | --- | --- |
| Uganda | Name | Also Fake | Mr | afn@expert.com | Yes | Yes | Local or national NGO representative | Senior Manager Research and Monitoring | Community Conservation NGO | Extensive experience in Community Conservation. |

## Appendix 6: Survey one template

Start of Block: 1. Survey information

Q1.1 **Patterns of “going to scale” among community-based natural resource management initiatives in Sub-Saharan Africa** **Survey One**  This is the first of two surveys. We are asking for your personal views and perspectives. You are encouraged to take time and answer these questions carefully and to look for other sources of evidence when deciding your responses.

Q1.2 **Consent** I confirm that I have read and understand the participant information sheet version 1.1 dated 02/09/22 for ***Patterns of ‘going to scale’ among area-based conservation interventions in Sub-Saharan Africa*** and have had the opportunity to ask questions which have been answered fully.  I understand that my participation is voluntary, and I am free to withdraw at any time, without giving any reason and without my legal rights being affected. I understand that data collected from me are a gift donated to [redacted] and that I will not personally benefit financially if this research leads to an invention and/or the successful development of a new test, medication treatment, product or service. I consent to take part in ***Patterns of ‘going to scale’ among area-based conservation interventions in Sub-Saharan Africa***. I give consent to being contacted about the possibility of taking part in other research studies. I understand that I may be invited to help co-author academic research outputs. In this case, I understand that the information I provide will remain anonymous but I will have the opportunity to be listed as a co-author on peer-review published articles.

- **Yes** (1)

Q1.2b I give consent for anonymous information collected during this survey to be used to support other research by an academic institution in the future, including those outside of the United Kingdom.

- **Yes** (4)
- **No** (5)

Q1.3 Study Management Group Principal Investigator: [redacted].

End of Block: 1. Survey information

Start of Block: 2. Survey questions

Q2.1 Instructions: In this survey, you will be answering questions about [Target initiative] initiatives in [focus country]. We would like you to think about the initiative across [focus country] as a whole and not focus on a particular example or place.

End of Block: 2. Survey questions

Start of Block: 3. Matching experts to interventions.

Q3.1 What is your family name?

________________________________________________________________

Q3.2 What is your given name?

________________________________________________________________

Q3.3 How many years have you been doing work related to the initiative?

▼ Less than five years (1) ... Over ten years (3)

Q3.4 What is your focus country?

▼ Botswana (30) ... Zimbabwe (23)

End of Block: 3. Matching experts to interventions.

Start of Block: 4. Instance-based relative adoption (part 1)

Q4.1 Instructions: This next set of questions asks about the local groups who have adopted [Target initiative] initiatives in [focus country]. [Adoption text A.]

Q4.2 Which statements best describes the local groups that adopt [Target initiative] initiatives?

- Households who adopts individually and not in a wider group (9)
- Some of the households in a single settlement who adopt as a group (12)
- All of the households in a single settlement who adopt as a group (13)
- Some or all households in multiple settlements who adopt as a group (14)
- Nomadic or semi-nomadic households who adopt as a group (15)
- Other (please describe) (7) __________________________________________________

End of Block: 4. Instance-based relative adoption (part 1)

Start of Block: 5. Instance-based relative adoption (part 2)

Q5.1 Instructions: This next set of questions asks you to estimate the total number of local groups across the whole country that have adopted the initiative since it was first established in law. If necessary, please look for sources of evidence such as websites, reports, and academic articles. [Adoption text B.]

| 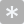 |
| --- |

Q5.2 What is the **lowest** realistic number of local groups who have adopted [Target initiative] initiatives? Please answer as a whole number.

________________________________________________________________

| 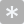 |
| --- |

Q5.3 What is the **highest** realistic number of local groups who have adopted [Target initiative] initiatives? Please answer as a whole number.

________________________________________________________________

| 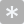 |
| --- |

Q5.4 What is your **best guess** for the number of local groups who have adopted [Target initiative] initiatives? Please answer as a whole number.

________________________________________________________________

End of Block: 5. Instance-based relative adoption (part 2)

Start of Block: 6. Abandonment

Q6.1 Out of the number of local groups who have ever adopted, please estimate the percentage who have stopped implementing the initiative. Please answer as a percentage (%). Stopping implementation means when a local group no longer implements some or all management activities for whatever reason. This abandonment could be official (e.g., formally terminating agreements) or unofficial (e.g., no active management).

|  | 0 | 10 | 20 | 30 | 40 | 50 | 60 | 70 | 80 | 90 | 100 |
| --- | --- | --- | --- | --- | --- | --- | --- | --- | --- | --- | --- |

| Percentage (%) who have stopped implementing () | 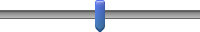 |
| --- | --- |

End of Block: 6. Abandonment

Start of Block: 7. Perceived relative adoption

Q7.1 Instructions: This next set of questions asks about your perceptions of how widely [Target initiative] initiatives have been adopted in [focus country]. [Adoption text A.]

Q7.2 To what extent do you agree with the statement: “[Target initiative] initiatives are rare in [focus country]”?

- Strongly disagree (6)
- Somewhat disagree (7)
- Neither agree nor disagree (8)
- Somewhat agree (9)
- Strongly agree (10)

Q7.3 How many local groups have adopted [Target initiative] initiatives in [focus country]?

- Very few local groups (1)
- Few local groups (4)
- Some local groups (5)
- Many local groups (6)
- Very many local groups (7)

Q7.4 How common are [Target initiative] initiatives in [focus country]?

- Very uncommon (1)
- Somewhat uncommon (4)
- Neither common nor uncommon (5)
- Somewhat common (6)
- Very common (7)

Q7.5 How many places have [Target initiative] initiatives in [focus country]?

- Very few places (1)
- Few places (4)
- Some places (5)
- Many places (6)
- Very many places (7)

Q7.6 To what extent do you agree with the statement: “[Target initiative] initiatives have been very widely adopted in [focus country]”?

- Strongly disagree (1)
- Somewhat disagree (4)
- Neither agree nor disagree (5)
- Somewhat agree (6)
- Strongly agree (7)

End of Block: 7. Perceived relative adoption

Start of Block: 8. Initiative characteristics (part 1)

Q8.1 Instructions: The next set of questions asks you about the [Target initiative] initiatives in [focus country].

Q8.2 In general, how do [Target initiative] initiatives affect participating local groups’ **income**? Please think about the following sources of income: Tourism Sale of harvested or farmed/ranched wild animals and plants Agricultural land availability Other sources

- Large reduction in local incomes (14)
- Small reduction in local incomes (15)
- No effect on local incomes (16)
- Small increase in local incomes (17)
- Large increase in local incomes (18)

Q8.3 In general, how do [Target initiative] initiatives affect participating local groups’ access to **natural resources** for **consumption**? Please think about the following natural resources: Wild foods such as animals, plants, or fungi. Building materials Firewood and charcoal Medicinal products  Other resources

- Large reduction in access to natural resources (11)
- Small reduction in access to natural resources (12)
- No effect on access to natural resources (13)
- Small increase in access to natural resources (14)
- Large increase in access to natural resources (15)

Q8.4 In general, how do [Target initiative] initiatives affect participating local groups’ access to **crop farmland** for **consumption**?

- Large reduction in crop farmland (11)
- Small reduction in crop farmland (12)
- No effect on crop farmland (13)
- Small increase in crop farmland (14)
- Large increase in crop farmland (15)

Q8.5 In general, how do [Target initiative] initiatives affect participating local groups’ access to **livestock farmland** for **consumption**?

- Large reduction in livestock farmland (15)
- Small reduction in livestock farmland (16)
- No effect on livestock farmland (17)
- Small increase in livestock farmland (18)
- Large increase in livestock farmland (19)

Q8.6 In general, how do [Target initiative] initiatives affect participating local groups’ access to **infrastructure**?
Please think about the following infrastructure: Schools Healthcare facilities Water sources Roads Other developments

- Large reduction in access to infrastructure (5)
- Small reduction in access to infrastructure (7)
- No effect on access to infrastructure (8)
- Small increase in access to infrastructure (9)
- Large increase in access to infrastructure (11)

Q8.7 In general, how do [Target initiative] initiatives affect **conflicts** between local people? This conflict might be within or between local groups or with other local people outside those groups.

- Large increase in conflict (5)
- Small increase in conflict (12)
- No effect on conflict (13)
- Small reduction in conflict (14)
- Large reduction in conflict (15)

Q8.8 In general, how do [Target initiative] initiatives affect local **traditions and cultures**?

- Greatly weakens traditions and cultures (15)
- Somewhat weakens traditions and cultures (16)
- No effect on traditions and cultures (17)
- Somewhat strengthens traditions and cultures (18)
- Greatly strengthens traditions and cultures (19)

Q8.9 In general, how do [Target initiative] initiatives affect participating local groups’ **control of natural resources**?

- Large reduction in control of resources (9)
- Small reduction in control of resources (10)
- No effect on control of resources (11)
- Small increase in control of resources (12)
- Large increase in control of resources (13)

Q8.10 **Overall**, how do [Target initiative] initiatives affect the lives of participating local groups? On average, across all cases of the initiative.

- Greatly worsens the lives of local groups (13)
- Moderately worsens the lives of local groups (15)
- Slightly worsens the lives of local groups (16)
- No effect on the lives of local groups (17)
- Slightly improves for the lives of local groups (18)
- Moderately improves the lives of local groups (19)
- Greatly improves the lives of local people (20)

Q8.11 In general, how fairly are the benefits and costs of [Target initiative] initiatives shared *within* participating local groups?
Fairness can include, for example, benefiting those: most in need, who invest the most resources into the initiative, who face the greatest costs from the initiative.

- The benefits and costs are very unfairly shared (1)
- The benefits and costs are mostly unfairly shared (4)
- The benefits and costs are mostly fairly shared (5)
- The benefits and costs are very fairly shared (6)
- Cannot answer: it varies a lot between local groups (7)

Q8.12 In general, how fairly are the benefits and costs of [target initiative] initiatives shared *between* participating local groups and non-participating residents?

- The benefits and costs are very unfairly shared (1)
- The benefits and costs are mostly unfairly shared (4)
- The benefits and costs are mostly fairly shared (5)
- The benefits and costs are very fairly shared (6)
- Cannot answer: it varies a lot between local groups (7)

End of Block: 8. Initiative characteristics (part 1)

Start of Block: 9. Initiative characteristics (part 2)

Q9.1 To what extent do you agree with the statement: “[Target initiative] initiatives focus on the most important needs of local groups”?

- Strongly disagree (7)
- Somewhat disagree (8)
- Neither agree nor disagree (9)
- Somewhat agree (10)
- Strongly agree (11)

Q9.2 In general, how easy is it for local groups to understand the processes for adopting and implementing [Target initiative] initiatives?

- Very difficult to understand (5)
- Slightly difficult to understand (7)
- Neither easy nor difficult (8)
- Slightly easy to understand (9)
- Very easy to understand (11)

Q9.3 How easy is it for local groups (who have not yet adopted) to see or learn about the benefits and costs of [Target initiative] initiatives? Local groups might learn about the benefits and costs from speaking to other local groups, from NGOs, from reading reports, etc.

- Very difficult to see or learn (15)
- Somewhat difficult to see or learn (16)
- Neither easy nor difficult (17)
- Somewhat easy to see or learn (18)
- Very easy to see or learn (19)

Q9.4 How flexible are the rules and management activities around how local groups implement [Target initiative] initiatives? Flexible means that the rules and management activities can be changed by local groups depending on local priorities and contexts.

- Very inflexible: The rules and management activities cannot be changed by local groups (4)
- Somewhat inflexible: Most of the rules and management activities cannot be changed by local groups (5)
- In the middle: Some rules and management activities can be changed by local groups (6)
- Somewhat flexible: Most of the rules and management activities can be changed by local groups (7)
- Very flexible: All of the rules and management activities can be changed by local groups (8)

End of Block: 9. Initiative characteristics (part 2)

Start of Block: 10. Adopter characteristics

Q10.1 Instructions: This next set of questions asks you about the characteristics of local groups.

Q10.2 How equal is the decision-making between members of local groups (regardless of their gender, social status, or the amount of money they have)?

- Decision-making is very unequal: some people have almost all control (4)
- Decision-making is mostly unequal: some people have most of the control (5)
- Decision-making is mostly equal: control is generally equal, but some people still have more control than others (6)
- Decision-making is very equal: all people have the same control (7)

Q10.3 Please select the statement that best describes communal land rights in [focus country]?

- Local groups have no legal rights to own communal land (7)
- Local groups have the legal right, but in practice it is only recognised in a some cases (8)
- Communities have the legal right, and it is recognised in most cases (9)
- Local groups have the legal right, and it is recognised in all cases (10)

Q10.4 Please select the statement that best describes communal natural resource rights in [focus country]?

- Local groups have **no legal rights** to use any natural resources on communal land (1)
- Local groups have **some legal rights** to use natural resources on communal land (4)
- Local groups have **legal rights to all** natural resources on communal land (5)

Q10.5 In general, what percentage of local people in rural [focus country] know about [Target initiative] initiatives? Please answer as a percentage (%). “Knowing” means if you asked them about the initiative, they could give a basic description of what it is.

|  | 0 | 10 | 20 | 30 | 40 | 50 | 60 | 70 | 80 | 90 | 100 |
| --- | --- | --- | --- | --- | --- | --- | --- | --- | --- | --- | --- |

| What percentage (%) know about the initiative? () | 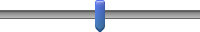 |
| --- | --- |

End of Block: 10. Adopter characteristics

Start of Block: 11. Context characteristics

Q11.1 Instructions: This next set of questions asks you about the wider environmental, social, and political situation.

Q11.2 Which best describes where [Target initiative] initiatives are allowed to be adopted? You can pick more than one option.

- Inside government protected areas or reserves (1)
- Next to government protected areas or reserves (2)
- In specific natural habitats, like forests or savannahs (3)
- On communal land (4)
- Any rural area in a country that contains mostly natural habitat (5)
- Any rural area, even with mostly agricultural land (7)
- Other (please describe) (6) __________________________________________________

Q11.3 What proportion of [focus country]’s geographical area is suitable for establishing [Target initiative] initiatives? Suitable means that the initiative could be established in a given location.

- Less than 20% of the country (1)
- Between 20% to 40% of the country (4)
- Between 40% to 60% of the country (5)
- Between 60% to 80% of the country (6)
- More than 80% of the country (7)

Q11.4 To what extent do national policies support local groups to adopt [Target initiative] initiatives?

- National policies make it very hard for local groups to adopt (4)
- National policies make it slightly hard for local groups to adopt (6)
- National policies make it neither hard nor easy for local groups to adopt (7)
- National policies make it slightly easy for local groups to adopt (8)
- National policies make it very easy for local groups to adopt (10)

Q11.5 How much technical assistance is available from external organisations to support local groups in adopting [Target initiative] initiatives? External organisations include government agencies, local and international NGOs, businesses, and funding bodies.

- No technical assistance (8)
- A small amount of technical assistance (9)
- A moderate amount of technical assistance (10)
- A large amount of technical assistance (11)
- A very large amount of technical assistance (12)

Q11.6 How much financial assistance is available from external organisations to support local groups in adopting [Target initiative] initiatives? External organisations include government agencies, local and international NGOs, businesses, and funding bodies.

- No financial assistance (8)
- A small amount of financial assistance (10)
- A moderate amount of financial assistance (11)
- A large amount of financial assistance (12)
- A very large amount of financial assistance (13)

End of Block: 11. Context characteristics

Start of Block: 12. Decision-making power

Q12.1 Instructions: This final set of questions asks you about who makes decisions.

Q12.2 In practice, which of the following best describes who decides if local groups adopt [Target initiative] initiatives or not? External organisations include government agencies, local and international NGOs, businesses, and funding bodies.

- Local groups have almost complete control over whether to adopt. External organisations have almost no control. (9)
- Local groups have most of the control over whether to adopt. External organisations have some control. (10)
- Local groups and external organisations have roughly equal control over whether local groups adopt. (11)
- External organisations have most of the control over whether local groups adopt. Local groups have some control. (12)
- External organisations have almost complete control over whether local groups adopt. Local groups have almost no control. (13)

Q12.3 Which of the following best describes who leads the adoption of [Target initiative] initiatives? External organisations include government agencies, local and international NGOs, businesses, and funding bodies.

- External organisations approach local groups. The adoption of the initiative is driven by external organisations. (1)
- There are existing relationships between local groups and external organisations. The adoption of the initiative is driven by local groups and external organisations together. (2)
- Local groups approach external organisations. The adoption of the initiative is driven by local groups. (3)

End of Block: 12. Decision-making power

Start of Block: 13. Snowball sampling

Q13.1 Optional: Before you finish, do you have any suggestions for other experts on [target initiative] we should contact to complete this survey? If you have more than five, please email [redacted].

- Suggestions 1: Name and email (6) __________________________________________________
- Suggestions 2: Name and email (7) __________________________________________________
- Suggestions 3: Name and email (8) __________________________________________________
- Suggestions 4: Name and email (9) __________________________________________________
- Suggestions 5: Name and email (10) __________________________________________________

End of Block: 13. Snowball sampling

## Appendix 7: Survey two template

Start of Block: Survey information 1

Q1.1 Thank you for completing the first round of the survey a few months ago!   Your previous answers have helped us identify factors that might be barriers to communities engaging with [target initiative].  We call these “scaling bottlenecks” because they might reduce the uptake and spread of [target initiative] in [focus country]. The results shown in this next survey do not say that [target initiative] are bad or should not be promoted. Instead, they show factors that could be addressed to increase the uptake and spread of [target initiative].    Please click the ‘***Next page***’ button to continue.

End of Block: Survey information 1

Start of Block: Survey information 2

Q2.1 This second survey has two goals: To ask for your thoughts and comments on these “scaling bottlenecks”. To ask if you want to be named as a co-author on an academic paper. This is on the last page of the survey. Please click the ‘***Next page’*** button to continue.

End of Block: Survey information 2

Start of Block: Survey information 3

Q4.1 In this survey, you will be answering questions about [target initiative] initiatives [focus country].  We would like you to think about the initiative across [focus country] as a whole and not focus on a particular example or place.

|  |  |
| --- | --- |

Q260 What is your title?

- Mr. (1)
- Mrs. (7)
- Ms. (8)
- Dr. (Doctor) (10)
- Prof. (Professor) (11)

Q13.4 Please select your name from the list.

- Click to write Choice 1 (1)
- Click to write Choice 2 (2)
- Click to write Choice 3 (3)

Q13.5 Is your name spelt correctly in the list above? It should be in the order of [*given name*] [*middle names*] [*family name*]*.* For example, Brian Kiggundu or Liberty Kiggundu*.* This is how your name will appear on the paper, so it must be provided correctly here.

- Yes (1)
- No (2)

Display This Question:

If Is your name spelt correctly in the list above? It should be in the order of [given name] [middle... = No

Q13.6 What is the correct spelling of your name, in the order [*given name*] [*middle names*] [*family name*]?

________________________________________________________________

End of Block: Survey information 3

Start of Block: Survey information 4

Q270 Each question has a diagram. Please watch the short video below that explains how to understand the diagram, then click the **“Next page”**. **VIDEO:**

End of Block: Survey information 4

Start of Block: Relative advantage

Q5.1 **Benefits and costs for local communities** The next questions show what experts said about the benefits and costs of engaging with [target initiative] for local groups.

| Page Break |  |
| --- | --- |

Q5.2 Many experts said that engaging with [target initiative] reduced incomes for participating local groups.  [FIGURE] Does this make it less likely that other local groups will engage with [target initiative] in the future? Incomes might be from tourism, the sale of harvested or farmed/ranched wild animals and plants, and other sources.

- Yes (4)
- No (5)

Display This Question:

If Many experts said that engaging with [target initiative] reduced incomes for participating local... = Yes

Q5.3 [If yes]: Why and what steps could be taken to improve incomes to increase future engagement with [target initiative]?

________________________________________________________________

________________________________________________________________

________________________________________________________________

________________________________________________________________

________________________________________________________________

Display This Question:

If Many experts said that engaging with [target initiative] reduced incomes for participating local... = No

Q5.4 [If no]: Why is this not a barrier to other local groups engaging with [target initiative]?

________________________________________________________________

________________________________________________________________

________________________________________________________________

________________________________________________________________

________________________________________________________________

| Page Break |  |
| --- | --- |

Q5.5 Many experts said that engaging with [target initiative] reduced access to natural resources for participating local groups.  [FIGURE] Does this make it less likely that other local groups will engage with [target initiative] in the future? Natural resources might include wild foods such as animals, plants, or fungi, building materials, firewood and charcoal, medicinal products, and other resources.

- Yes (4)
- No (5)

Display This Question:

If Many experts said that engaging with [target initiative] reduced access to natural resources for... = Yes

Q5.6 [If yes]: Why and what steps could be taken to improve access to natural resources to increase future engagement with [target initiative]?

________________________________________________________________

________________________________________________________________

________________________________________________________________

________________________________________________________________

________________________________________________________________

Display This Question:

If Many experts said that engaging with [target initiative] reduced access to natural resources for... = No

Q5.7 [If no]: Why is this not a barrier to other local groups engaging with [target initiative]?

________________________________________________________________

________________________________________________________________

________________________________________________________________

________________________________________________________________

________________________________________________________________

| Page Break |  |
| --- | --- |

Q5.8 Many experts said that engaging with [target initiative] reduced access to crop farmland for participating local groups. [FIGURE] Does this make it less likely that other local groups will engage with [target initiative] in the future?

- Yes (4)
- No (5)

Display This Question:

If Many experts said that engaging with [target initiative] reduced access to crop farmland for part... = Yes

Q5.9 [If yes]: Why and what steps could be taken to improve access to crop farmland to increase future engagement with [target initiative]?

________________________________________________________________

________________________________________________________________

________________________________________________________________

________________________________________________________________

________________________________________________________________

Display This Question:

If Many experts said that engaging with [target initiative] reduced access to crop farmland for part... = No

Q5.10 [If no]: Why is this not a barrier to other local groups engaging with [target initiative]?

________________________________________________________________

________________________________________________________________

________________________________________________________________

________________________________________________________________

________________________________________________________________

| Page Break |  |
| --- | --- |

Q5.11 Many experts said that engaging with [target initiative] reduced access to livestock farmland for participating local groups. [FIGURE] Does this make it less likely that other local groups will engage with [target initiative] in the future?

- Yes (4)
- No (5)

Display This Question:

If Many experts said that engaging with [target initiative] reduced access to livestock farmland for... = Yes

Q5.12 [If yes]: Why and what steps could be taken to improve access to livestock farmland to increase future engagement with [target initiative]?

________________________________________________________________

________________________________________________________________

________________________________________________________________

________________________________________________________________

________________________________________________________________

Display This Question:

If Many experts said that engaging with [target initiative] reduced access to livestock farmland for... = No

Q5.13 [If no]: Why is this not a barrier to other local groups engaging with [target initiative]?

________________________________________________________________

________________________________________________________________

________________________________________________________________

________________________________________________________________

________________________________________________________________

| Page Break |  |
| --- | --- |

Q5.14 Many experts said that engaging with [target initiative] reduced access to infrastructure for participating local groups. [FIGURE] Does this make it less likely that other local groups will engage with [target initiative] in the future? Infrastructure includes things like schools, healthcare facilities, water sources, roads, and other developments.

- Yes (4)
- No (5)

Display This Question:

If Many experts said that engaging with [target initiative] reduced access to infrastructure for par... = Yes

Q5.15 [If yes]: Why and what steps could be taken to improve access to infrastructure to increase future engagement with [target initiative]?

________________________________________________________________

________________________________________________________________

________________________________________________________________

________________________________________________________________

________________________________________________________________

Display This Question:

If Many experts said that engaging with [target initiative] reduced access to infrastructure for par... = No

Q5.16 [If no]: Why is this not a barrier to other local groups engaging with [target initiative]?

________________________________________________________________

________________________________________________________________

________________________________________________________________

________________________________________________________________

________________________________________________________________

| Page Break |  |
| --- | --- |

Q5.17 Many experts said that engaging with [target initiative] increased conflict between local people. [FIGURE] Does this make it less likely that other local groups will engage with [target initiative] in the future? This conflict might be within or between local groups or with other local people outside those groups.

- Yes (4)
- No (5)

Display This Question:

If Many experts said that engaging with [target initiative] increased conflict between local people.... = Yes

Q5.18 [If yes]: Why and what steps could be taken to reduce conflict to increase future engagement with [target initiative]?

________________________________________________________________

________________________________________________________________

________________________________________________________________

________________________________________________________________

________________________________________________________________

Display This Question:

If Many experts said that engaging with [target initiative] increased conflict between local people.... = No

Q5.19 [If no]: Why is this not a barrier to other local groups engaging with [target initiative]?

________________________________________________________________

________________________________________________________________

________________________________________________________________

________________________________________________________________

________________________________________________________________

| Page Break |  |
| --- | --- |

Q5.20 Many experts said that engaging with [target initiative] weakened local traditions and cultures. [FIGURE] Does this make it less likely that other local groups will engage with [target initiative] in the future?

- Yes (4)
- No (5)

Display This Question:

If Many experts said that engaging with [target initiative] weakened local traditions and cultures.... = Yes

Q5.21 [If yes]: Why and what steps could be taken so that local traditions and cultures are protected to increase future engagement with [target initiative]?

________________________________________________________________

________________________________________________________________

________________________________________________________________

________________________________________________________________

________________________________________________________________

Display This Question:

If Many experts said that engaging with [target initiative] weakened local traditions and cultures.... = No

Q5.22 [If no]: Why is this not a barrier to other local groups engaging with [target initiative]?

________________________________________________________________

________________________________________________________________

________________________________________________________________

________________________________________________________________

________________________________________________________________

| Page Break |  |
| --- | --- |

Q5.23 Many experts said that engaging with [target initiative] reduced local groups’ control of natural resources. [FIGURE] Does this make it less likely that other local groups will engage with [target initiative] in the future?

- Yes (4)
- No (5)

Display This Question:

If Many experts said that engaging with [target initiative] reduced local groups’ control of natural... = Yes

Q5.24 [If yes]: Why and what steps could be taken to improve local control of natural resources to increase future engagement with [target initiative]?

________________________________________________________________

________________________________________________________________

________________________________________________________________

________________________________________________________________

________________________________________________________________

Display This Question:

If Many experts said that engaging with [target initiative] reduced local groups’ control of natural... = No

Q5.25 [If no]: Why is this not a barrier to other local groups engaging with [target initiative]?

________________________________________________________________

________________________________________________________________

________________________________________________________________

________________________________________________________________

________________________________________________________________

| Page Break |  |
| --- | --- |

Q5.26 Many experts said that engaging with [target initiative] worsened the lives of participating local groups overall. [FIGURE] Does this make it less likely that other local groups will engage with [target initiative] in the future?

- Yes (4)
- No (5)

Display This Question:

If Many experts said that engaging with [target initiative] worsened the lives of participating loca... = Yes

Q5.27 [If yes]: Why and what steps could be taken to improve the impact of [target initiative] on the lives of local people to increase future engagement?

________________________________________________________________

________________________________________________________________

________________________________________________________________

________________________________________________________________

________________________________________________________________

Display This Question:

If Many experts said that engaging with [target initiative] worsened the lives of participating loca... = No

Q5.28 [If no]: Why is this not a barrier to other local groups engaging with [target initiative]?

________________________________________________________________

________________________________________________________________

________________________________________________________________

________________________________________________________________

________________________________________________________________

End of Block: Relative advantage

Start of Block: Fair sharing of benefits and costs

Q6.1 **Fair sharing of benefits and costs**  The next questions show what experts said about how fairly the benefits and costs of [target initiative] are shared locally.    Each question has a diagram. The coloured bars represent the percentage of experts who gave each response. Orange and red show the number of ‘negative’ responses.

| Page Break |  |
| --- | --- |

Q6.2 Many experts said that the benefits and costs of [target initiative] were unfairly shared **within participating local groups** (those directly engaged in initiatives).   [FIGURE]   Does this make it less likely that other local groups will engage with [target initiative] in the future?

- Yes (4)
- No (5)

Display This Question:

If Many experts said that the benefits and costs of [target initiative] were unfairly shared within... = Yes

Q6.3 [If yes]: Why and what steps could be taken to improve fairness within groups to increase future engagement?

________________________________________________________________

________________________________________________________________

________________________________________________________________

________________________________________________________________

________________________________________________________________

Display This Question:

If Many experts said that the benefits and costs of [target initiative] were unfairly shared within... = No

Q6.4 [If no]: Why is this not a barrier to other local groups engaging with [target initiative]?

________________________________________________________________

________________________________________________________________

________________________________________________________________

________________________________________________________________

________________________________________________________________

| Page Break |  |
| --- | --- |

Q6.5 Many experts said that the benefits and costs of [target initiative] were unfairly shared ***between*** participating local groups (who directly participate) and ***other local people***(who do not participate).   [FIGURE]   Does this make it less likely that other local groups will engage with [target initiative] in the future?

- Yes (4)
- No (5)

Display This Question:

If Many experts said that the benefits and costs of [target initiative] were unfairly shared between... = Yes

Q6.6 [If yes]: Why and what steps could be taken to improve fairness between local groups and other local people to increase future engagement?

________________________________________________________________

________________________________________________________________

________________________________________________________________

________________________________________________________________

________________________________________________________________

Display This Question:

If Many experts said that the benefits and costs of [target initiative] were unfairly shared between... = No

Q6.7 [If no]: Why is this not a barrier to other local groups engaging with [target initiative]?

________________________________________________________________

________________________________________________________________

________________________________________________________________

________________________________________________________________

________________________________________________________________

End of Block: Fair sharing of benefits and costs

Start of Block: Other factors to do with the initiative

Q7.1 Other factors to do with the initiative   The next questions show what experts said about other factors to do with [target initiative].

| Page Break |  |
| --- | --- |

Q7.2 Many experts thought that [target initiative] ***did not*** focus on the most important needs of local groups. [FIGURE] Is this a barrier for other local groups engaging with [target initiative] in the future?

- Yes (4)
- No (5)

Display This Question:

If Many experts thought that [target initiative] did not focus on the most important needs of local... = Yes

Q7.3 [If yes]: Why and what steps could be taken so that [target initiative] was more focused on the needs of local groups to increase future engagement?

________________________________________________________________

________________________________________________________________

________________________________________________________________

________________________________________________________________

________________________________________________________________

Display This Question:

If Many experts thought that [target initiative] did not focus on the most important needs of local... = No

Q7.4 [If no]: Why is this not a barrier to other local groups engaging with [target initiative]?

________________________________________________________________

________________________________________________________________

________________________________________________________________

________________________________________________________________

________________________________________________________________

| Page Break |  |
| --- | --- |

Q7.5 Many experts said it was difficult for local groups to understand the process for engaging with and implementing [target initiative].   [FIGURE]   Is this a barrier for other local groups engaging with [target initiative] in the future?   “Engaging with and implementing” means the steps required to establish and implement management activities related to [target initiative].

- Yes (4)
- No (5)

Display This Question:

If Many experts said it was difficult for local groups to understand the process for engaging with a... = Yes

Q7.6 [If yes]: Why and what steps could be taken to make it easier for local groups to understand [target initiative] to increase future engagement?

________________________________________________________________

________________________________________________________________

________________________________________________________________

________________________________________________________________

________________________________________________________________

Display This Question:

If Many experts said it was difficult for local groups to understand the process for engaging with a... = No

Q7.7 [If no]: Why is this not a barrier to other local groups engaging with [target initiative]?

________________________________________________________________

________________________________________________________________

________________________________________________________________

________________________________________________________________

________________________________________________________________

| Page Break |  |
| --- | --- |

Q7.8 Many experts said it was difficult for local groups to see or learn about the benefits and costs of engaging with [target initiative]. [FIGURE] Is this a barrier for other local groups engaging with [target initiative] in the future?  Local groups might learn about the benefits and costs from speaking to other local groups, from NGOs, from reading reports, etc.

- Yes (4)
- No (5)

Display This Question:

If Many experts said it was difficult for local groups to see or learn about the benefits and costs... = Yes

Q7.9 [If yes]: Why and what steps could be taken to make it easier for local groups to learn about the benefits and costs of [target initiative] to increase future engagement?

________________________________________________________________

________________________________________________________________

________________________________________________________________

________________________________________________________________

________________________________________________________________

Display This Question:

If Many experts said it was difficult for local groups to see or learn about the benefits and costs... = No

Q7.10 [If no]: Why is this not a barrier to other local groups engaging with [target initiative]?

________________________________________________________________

________________________________________________________________

________________________________________________________________

________________________________________________________________

________________________________________________________________

| Page Break |  |
| --- | --- |

Q7.11 Many experts said the rules and management activities around [target initiative] were inflexible. [FIGURE] Is this a barrier for other local groups engaging with [target initiative] in the future?   Flexible means that the rules and management activities can be changed by local groups depending on local priorities and contexts.

- Yes (4)
- No (5)

Display This Question:

If Many experts said the rules and management activities around [target initiative] were inflexible.... = Yes

Q7.12 [If yes]: Why, and what steps could be taken to improve the flexibility around the rules and management activities of [target initiative] to increase future engagement?

________________________________________________________________

________________________________________________________________

________________________________________________________________

________________________________________________________________

________________________________________________________________

Display This Question:

If Many experts said the rules and management activities around [target initiative] were inflexible.... = No

Q7.13 [If no]: Why is this not a barrier to other local groups engaging with [target initiative]?

________________________________________________________________

________________________________________________________________

________________________________________________________________

________________________________________________________________

________________________________________________________________

End of Block: Other factors to do with the initiative

Start of Block: Decision-making and rights

Q8.1 Decision-making and rights   The next questions show what experts said about decision-making within local groups and rights recognition within [focus country].

| Page Break |  |
| --- | --- |

Q8.2 Many experts thought decision-making between members of local groups was unequal. [FIGURE] Is this a barrier for other local groups engaging with [target initiative] in the future?   Equal-decision making means people have an equal say in decisions regardless of their gender, social status, or amount of money.

- Yes (4)
- No (5)

Display This Question:

If Many experts thought decision-making between members of local groups was unequal. [FIGURE] Is thi... = Yes

Q8.3 [If yes]: Why and what steps could be taken to make decision-making more equal to increase future engagement?

________________________________________________________________

________________________________________________________________

________________________________________________________________

________________________________________________________________

________________________________________________________________

Display This Question:

If Many experts thought decision-making between members of local groups was unequal. [FIGURE] Is thi... = No

Q8.4 [If no]: Why is this not a barrier to other local groups engaging with [target initiative]?

________________________________________________________________

________________________________________________________________

________________________________________________________________

________________________________________________________________

________________________________________________________________

| Page Break |  |
| --- | --- |

Q8.5 Many experts said local groups either lack rights to own communal land or that these rights are only sometimes recognised in practice. [FIGURE] Is this a barrier for other local groups engaging with [target initiative] in the future?

- Yes (4)
- No (5)

Display This Question:

If Many experts said local groups either lack rights to own communal land or that these rights are o... = Yes

Q8.6 [If yes]: Why and what steps could be taken to strengthen communal land rights to increase future engagement?

________________________________________________________________

________________________________________________________________

________________________________________________________________

________________________________________________________________

________________________________________________________________

Display This Question:

If Many experts said local groups either lack rights to own communal land or that these rights are o... = No

Q8.7 [If no]: Why is this not a barrier to other local groups engaging with [target initiative]?

________________________________________________________________

________________________________________________________________

________________________________________________________________

________________________________________________________________

________________________________________________________________

| Page Break |  |
| --- | --- |

Q8.8 Many experts said local groups have no legal rights to use natural resources on communal land. [FIGURE] Is this a barrier for other local groups engaging with [target initiative] in the future?

- Yes (4)
- No (5)

Display This Question:

If Many experts said local groups have no legal rights to use natural resources on communal land. [F... = Yes

Q8.9 [If yes]: Why and what steps could be taken to strengthen communal natural resource rights to increase future engagement?

________________________________________________________________

________________________________________________________________

________________________________________________________________

________________________________________________________________

________________________________________________________________

Display This Question:

If Many experts said local groups have no legal rights to use natural resources on communal land. [F... = No

Q8.10 [If no]: Why is this not a barrier to other local groups engaging with [target initiative]?

________________________________________________________________

________________________________________________________________

________________________________________________________________

________________________________________________________________

________________________________________________________________

End of Block: Decision-making and rights

Start of Block: Awareness of the initiative

Q9.1 Awareness of the initiative   The next questions show what experts said about the levels of awareness of [target initiative] among rural people in [focus country].

| Page Break |  |
| --- | --- |

Q9.2 On average, experts thought that less than [awareness %] of local people in rural [focus country] knew about [target initiative]. [FIGURE] Is this a barrier for other local groups engaging with [target initiative] in the future?   “Knowing” means if you asked them about the initiative, they could give a basic description of what it is.

- Yes (4)
- No (5)

Display This Question:

If On average, experts thought that less than [awareness %] of local people in rural [focus country]... = Yes

Q9.3 [If yes]: Why, and what steps could be taken to increase awareness of [target initiative] to increase future engagement?

________________________________________________________________

________________________________________________________________

________________________________________________________________

________________________________________________________________

________________________________________________________________

Display This Question:

If On average, experts thought that less than [awareness %] of local people in rural [focus country]... = No

Q9.4 [If no]: Why is this not a barrier to other local groups engaging with [target initiative]?

________________________________________________________________

________________________________________________________________

________________________________________________________________

________________________________________________________________

________________________________________________________________

End of Block: Awareness of the initiative

Start of Block: Geographic suitability

Q10.1 Geographic suitability The next questions show what experts said about the proportion of [focus country] suitable for [target initiative].

| Page Break |  |
| --- | --- |

Q10.2 On average, experts thought that only [suitable %] of [focus country]’s geographical area was suitable for establishing [target initiative]. [FIGURE] Is this a barrier for other local groups engaging with [target initiative] in the future?   Suitable means that the initiative could be established in a given location.

- Yes (4)
- No (5)

Display This Question:

If On average, experts thought that only [suitable %] of [focus country]’s geographical area was sui... = Yes

Q10.3 [If yes]: Why, and what steps could be taken to make [target initiative] suitable for more areas in [focus country]?

________________________________________________________________

________________________________________________________________

________________________________________________________________

________________________________________________________________

________________________________________________________________

Display This Question:

If On average, experts thought that only [suitable %] of [focus country]’s geographical area was sui... = No

Q10.4 [If no]: Why is this not a barrier to other local groups engaging with [target initiative]?

________________________________________________________________

________________________________________________________________

________________________________________________________________

________________________________________________________________

________________________________________________________________

End of Block: Geographic suitability

Start of Block: Support

Q11.1 External support  The next questions show what experts said about national policies and external support for [target initiative] in [focus country].

| Page Break |  |
| --- | --- |

Q11.2 Many experts thought that national policies made it hard for local groups to engage with [target initiative]. [FIGURE] Is this a barrier for other local groups engaging with [target initiative] in the future?

- Yes (4)
- No (5)

Display This Question:

If Many experts thought that national policies made it hard for local groups to engage with [target... = Yes

Q11.3 [If yes]: Why, and what steps could be taken to revise and improve national policies to increase future engagement with [target initiative]?

________________________________________________________________

________________________________________________________________

________________________________________________________________

________________________________________________________________

________________________________________________________________

Display This Question:

If Many experts thought that national policies made it hard for local groups to engage with [target... = No

Q11.4 [If no]: Why is this not a barrier to other local groups engaging with [target initiative]?

________________________________________________________________

________________________________________________________________

________________________________________________________________

________________________________________________________________

________________________________________________________________

| Page Break |  |
| --- | --- |

Q11.5 Many experts said that only a small amount of ***technical*** assistance was available from external organisations to help local groups engage with [target initiative].  [FIGURE] Is this a barrier for other local groups engaging with [target initiative] in the future?   External organisations include government agencies, local and international NGOs, businesses, and funding bodies.

- Yes (4)
- No (5)

Display This Question:

If Many experts said that only a small amount of technical assistance was available from external or... = Yes

Q11.6 [If yes]: Why, and what steps could be taken to increase the amount of ***technical*** assistance from external organisations to increase future engagement?

________________________________________________________________

________________________________________________________________

________________________________________________________________

________________________________________________________________

________________________________________________________________

Display This Question:

If Many experts said that only a small amount of technical assistance was available from external or... = No

Q11.7 [If no]: Why is this not a barrier to other local groups engaging with [target initiative]?

________________________________________________________________

________________________________________________________________

________________________________________________________________

________________________________________________________________

________________________________________________________________

| Page Break |  |
| --- | --- |

Q11.8 Many experts said that only a small amount of ***financial*** assistance was available from external organisations to help local groups engage with [target initiative].  [FIGURE] Is this a barrier for other local groups engaging with [target initiative] in the future?   External organisations include government agencies, local and international NGOs, businesses, and funding bodies.

- Yes (4)
- No (5)

Display This Question:

If Many experts said that only a small amount of financial assistance was available from external or... = Yes

Q11.9 [If yes]: Why, and what steps could be taken to increase the amount of ***financial*** assistance from external organisations to increase future engagement?

________________________________________________________________

________________________________________________________________

________________________________________________________________

________________________________________________________________

________________________________________________________________

Display This Question:

If Many experts said that only a small amount of financial assistance was available from external or... = No

Q11.10 [If no]: Why is this not a barrier to other local groups engaging with [target initiative]?

________________________________________________________________

________________________________________________________________

________________________________________________________________

________________________________________________________________

________________________________________________________________

End of Block: Support

Start of Block: Leader

Q12.1 Who drives engagement  The next questions show what experts said about who decides and who leads the process of local groups engaging with [target initiative].

| Page Break |  |
| --- | --- |

Q12.2 Many experts said that external organisations have most of the control over whether local groups engage with [target initiative] or not.  [FIGURE] Is this a barrier for other local groups engaging with [target initiative] in the future?   External organisations include government agencies, local and international NGOs, businesses, and funding bodies.

- Yes (4)
- No (5)

Display This Question:

If Many experts said that external organisations have most of the control over whether local groups... = Yes

Q12.3 [If yes]: What steps could be taken so local groups have more control over whether to engage or not?

________________________________________________________________

________________________________________________________________

________________________________________________________________

________________________________________________________________

________________________________________________________________

Display This Question:

If Many experts said that external organisations have most of the control over whether local groups... = No

Q12.6 [If no]: Why should local groups not have greater control over whether they engage with [target initiative] or not?

________________________________________________________________

________________________________________________________________

________________________________________________________________

________________________________________________________________

________________________________________________________________

| Page Break |  |
| --- | --- |

Q12.4 Many experts said that external organisations lead the process of local groups engaging with [target initiative]. [FIGURE] Is this a barrier for other local groups engaging with [target initiative] in the future?   Leading means that external organisations approach local groups first and that they drive the process of local groups engaging with [target initiative].

- Yes (4)
- No (5)

Display This Question:

If Many experts said that external organisations lead the process of local groups engaging with [tar... = Yes

Q12.5 [If yes]: Why, and what steps could be taken so local groups have greater leadership to increase future engagement?

________________________________________________________________

________________________________________________________________

________________________________________________________________

________________________________________________________________

________________________________________________________________

Display This Question:

If Many experts said that external organisations lead the process of local groups engaging with [tar... = No

Q12.7 [If no]: Why should local groups not have greater leadership in the process of engaging with [target initiative]?

________________________________________________________________

________________________________________________________________

________________________________________________________________

________________________________________________________________

________________________________________________________________

End of Block: Leader

Start of Block: Block 11

Q13.1 Do you want to be a co-author?  We plan to submit a paper on this study to the journal *Conservation Biology*. We invite all experts to be co-authors on the manuscript if they want. This would involve reviewing the manuscript and providing their approval for us to submit (within the next two months).

Q13.2 Would you like to be a named co-author on the paper?

- Yes (1)
- No (2)
- I don’t know - I want further information before I decide (3)

Display This Question:

If Would you like to be a named co-author on the paper? = I don’t know - I want further information before I decide

Q13.3 If you would like more information before you decide, please email [redacted].

Display This Question:

If Would you like to be a named co-author on the paper? = Yes

Q13.7 What is the name of the organisation you work for? We need to provide this information when we submit the paper. If you do not want your organisation named or do not work for an organisation, please put “NA” in the box below.

________________________________________________________________

Display This Question:

If Would you like to be a named co-author on the paper? = Yes

Q13.8 What town or city is your organisation in? If you do not want your organisation named or do not work for an organisation, please put “NA” in the box below.

________________________________________________________________

Display This Question:

If Would you like to be a named co-author on the paper? = Yes

Q13.9 What email address would you like us to use for messages about the paper?

________________________________________________________________

End of Block: Block 11

## Appendix 8: Statistical analyses

We modelled 15 ordinally distributed outcome variables ($Outcome$) corresponding to 15 Likert-scaled survey questions ($q$) with ordered categorical response levels ($j)$. The explanatory variable of interest was a categorical variable describing the expert group type ($Group$), with an estimated slope coefficient of $\beta1$. We allowed for varying intercepts for each intervention ($i$, to control for differences in responses between interventions) by estimating the parameter ${\beta0}_{i}$ as well as the threshold for each categorical response level ($\alpha_{j})$.

$${logit[P(Outcome}_{q}\leq j)]\sim\alpha_{j}+{\beta0}_{i}+\beta1\cdot Group$$

We also modelled a composite variable derived from the eight sub-questions clustered within the theme of relative advantage, where observations were stacked row-wise, allowing relative advantage to be treated as a single response variable ($Advantage)$. In addition to the notation described above, we also allowed for varying intercepts for each expert ($e$, to account for dependencies in responses from the same individual) by estimating the parameter ${\beta2}_{e}$.

$$logit[P (Advantage\leq j)]\sim\alpha_{j}+{\beta0}_{i}+\beta1\cdot Group+{\beta2}_{e}$$

We implemented these analyses using the R package ‘brms’ (Bürkner 2017), using the software’s default weakly informative priors, the cumulative logit link function for ordinal response data, and 2,000 burn-in and 4,000 post-burn-in iterations (apart from where stated otherwise in *Appendix 10: Variation between experts*). We performed model diagnostics following the WAMBS (When to worry and how to Avoid the Misuse of Bayesian Statistics)-Checklist, with all models considered stable (Depaoli & Van de Schoot 2017). We report credible differences in responses between expert groups. Specifically, we calculated the predicted probability of a ‘negative’ response (e.g., answering “small reduction in income” or “large reduction in income”, following the above example) along with 95% credibility intervals (CI). If the credibility interval for one group did not overlap with the predicted probability for another group, we considered this a credible difference. We compared all groups and reported those that differed from other groups.

## Appendix 9: Likert-scaled plots of results


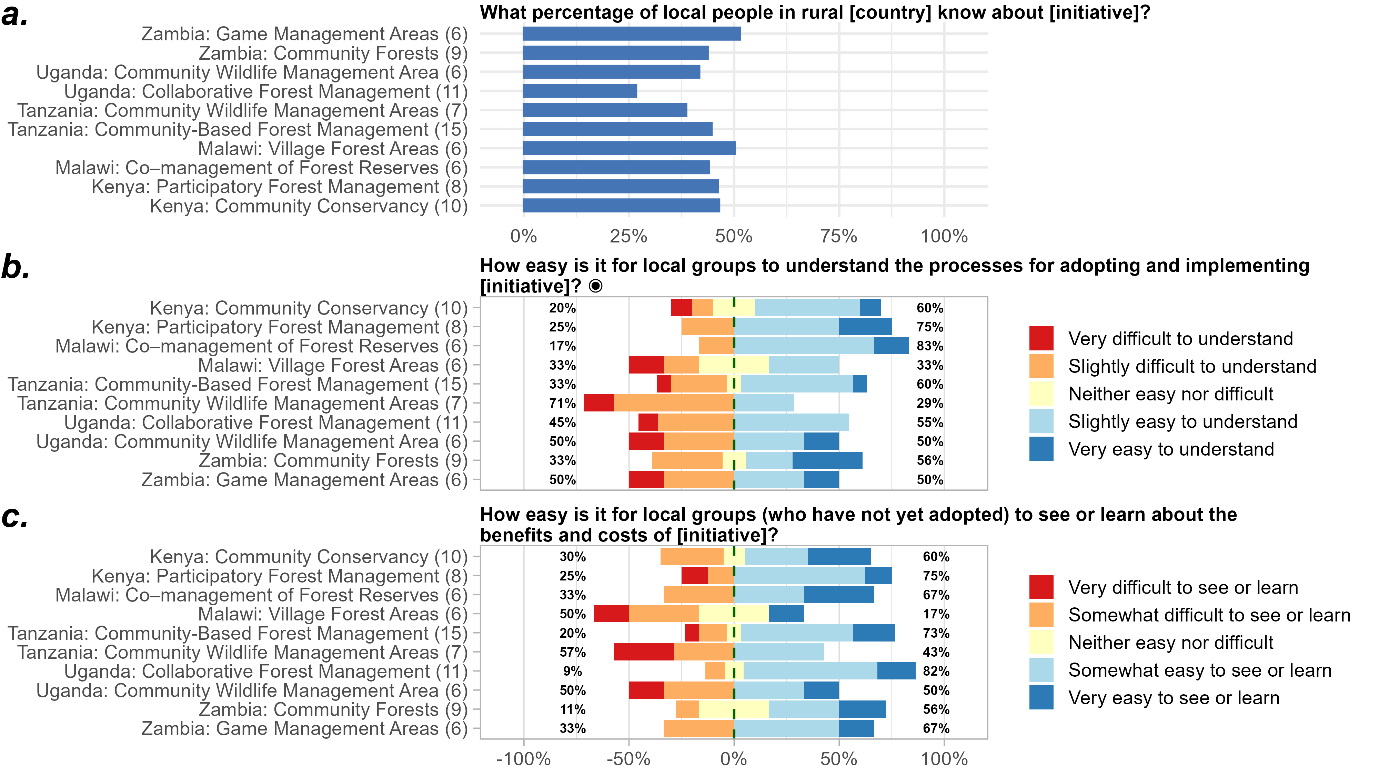


Figure 1. Judgments from 84 experts on potential scaling bottlenecks related to awareness (a.) and ease of understanding (b.) and observation of benefits (c.). Key: ◉ = indicates where there was credible variability between groups of experts.


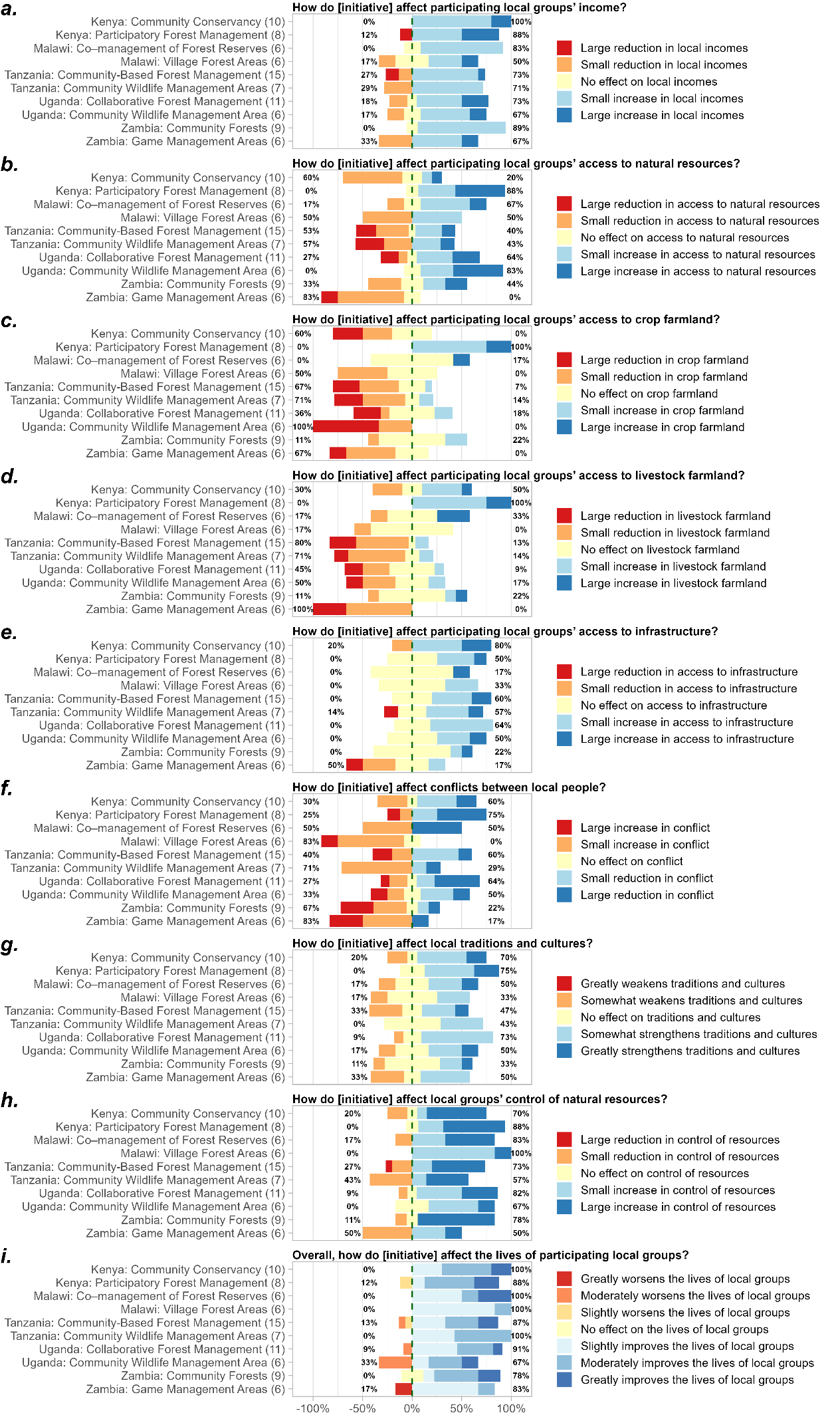


Figure 2. Judgments from 84 experts on potential scaling bottlenecks related to specific benefits and costs of adoption for local groups (a.-h.) and the overall impact of adoption on local people’s lives (i.).


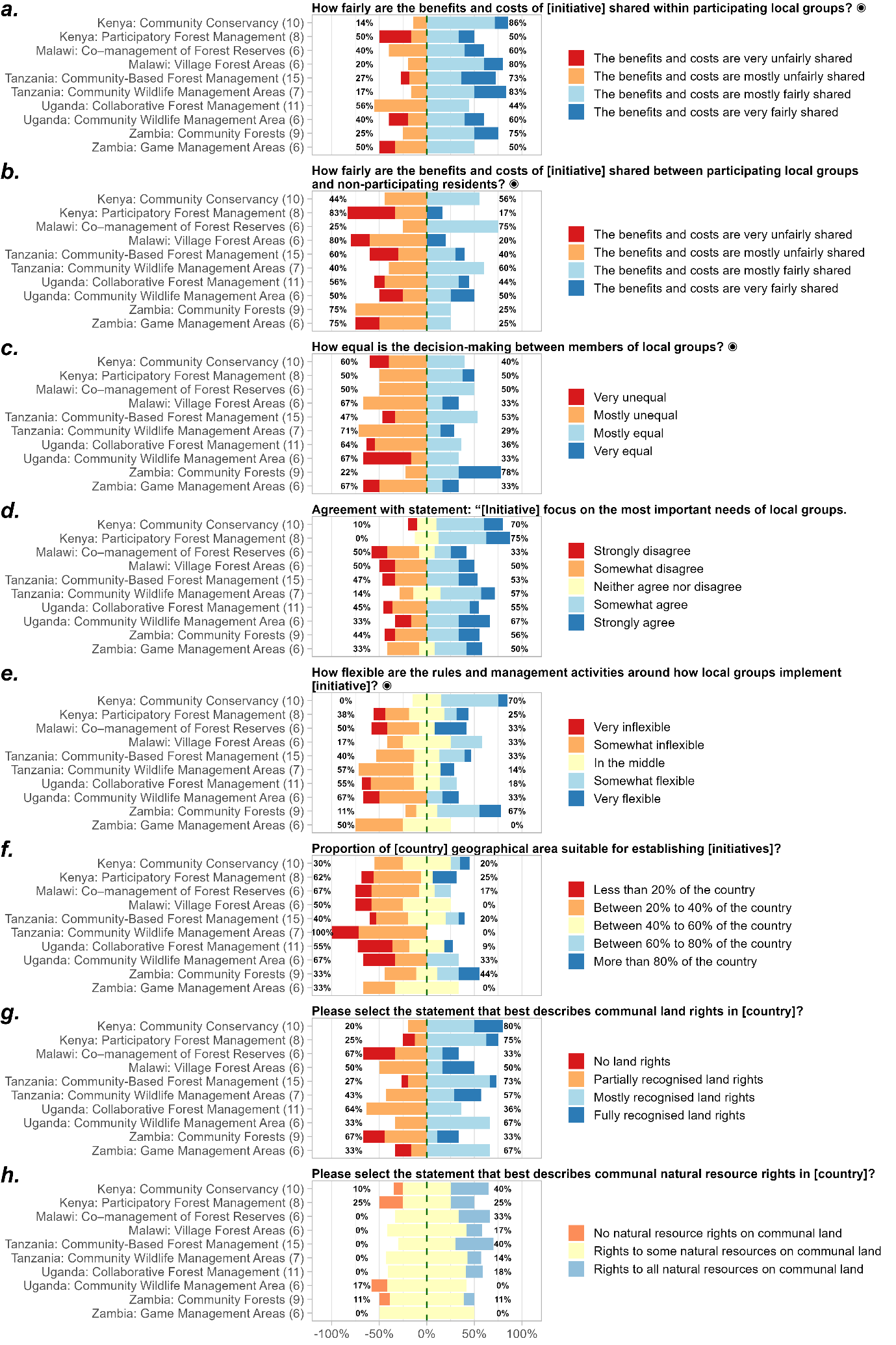


Figure 3. Judgments from 84 experts on potential scaling bottlenecks related to other factors related to motivation that may influence local groups’ adoption of initiatives (a.-h.). Key: ◉ = indicates where there was significant variability between groups of experts.


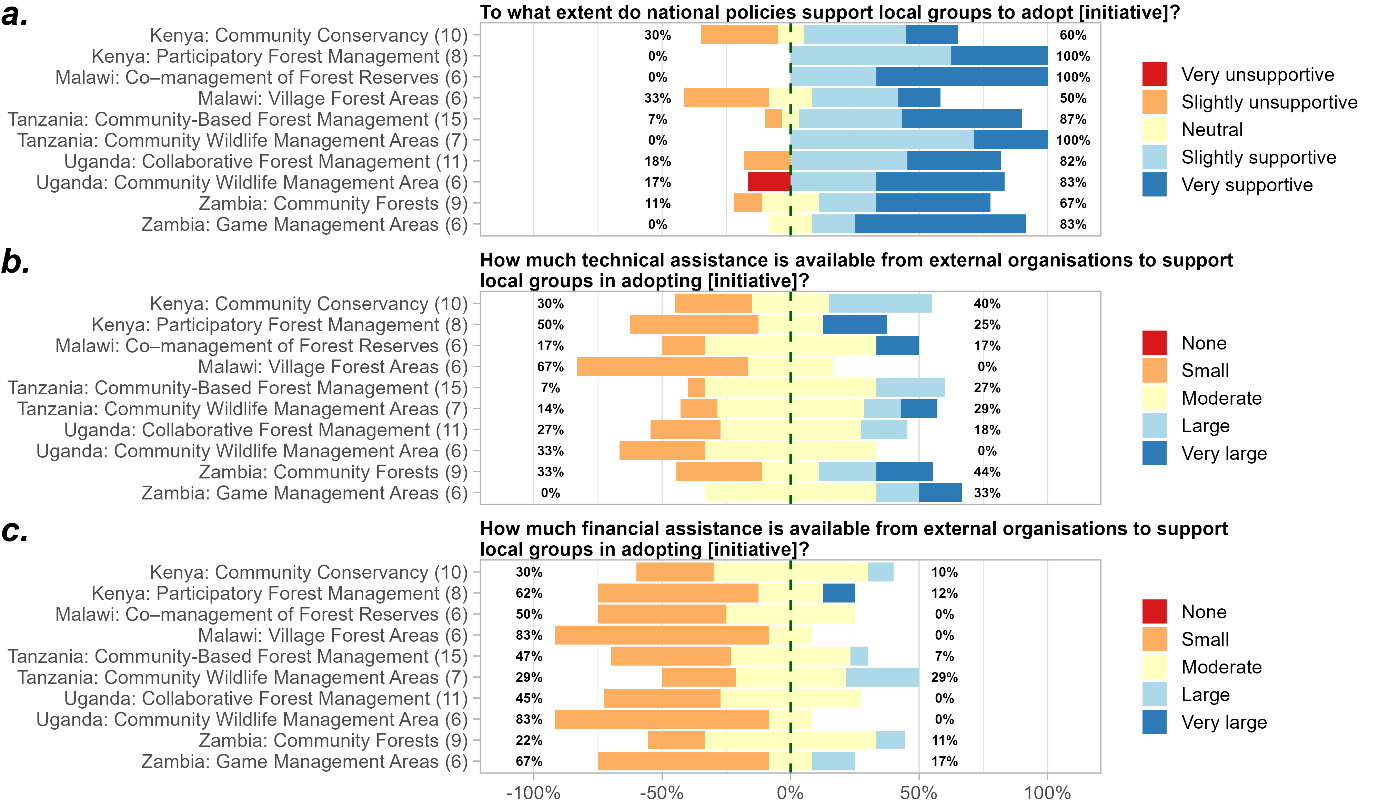


Figure 4. Judgments from 84 experts on potential scaling bottlenecks related to support available to local groups to adopt (a.-c.).


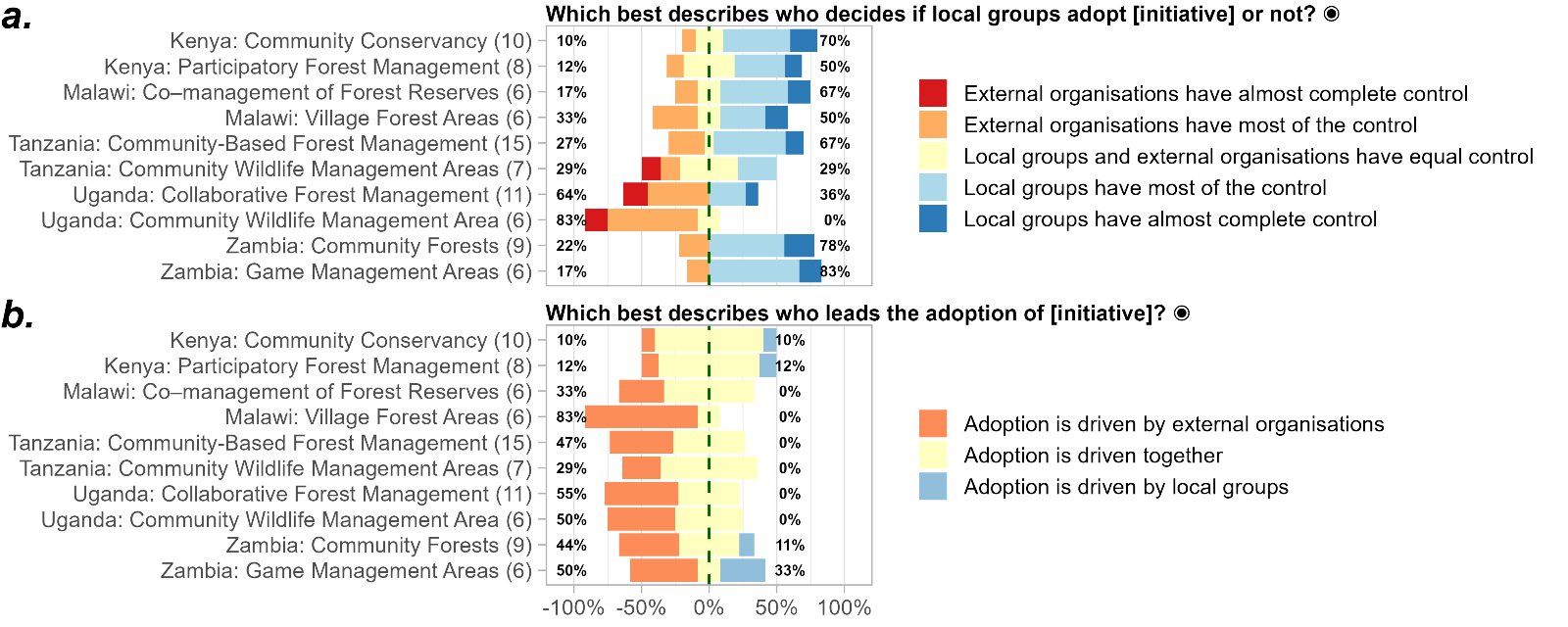


Figure 5. Judgments from 84 experts on which actors drive (a.) and control (b.) the adoption process. Key: ◉ = indicates where there was significant variability between groups of experts.

## Appendix 10: Variation between experts


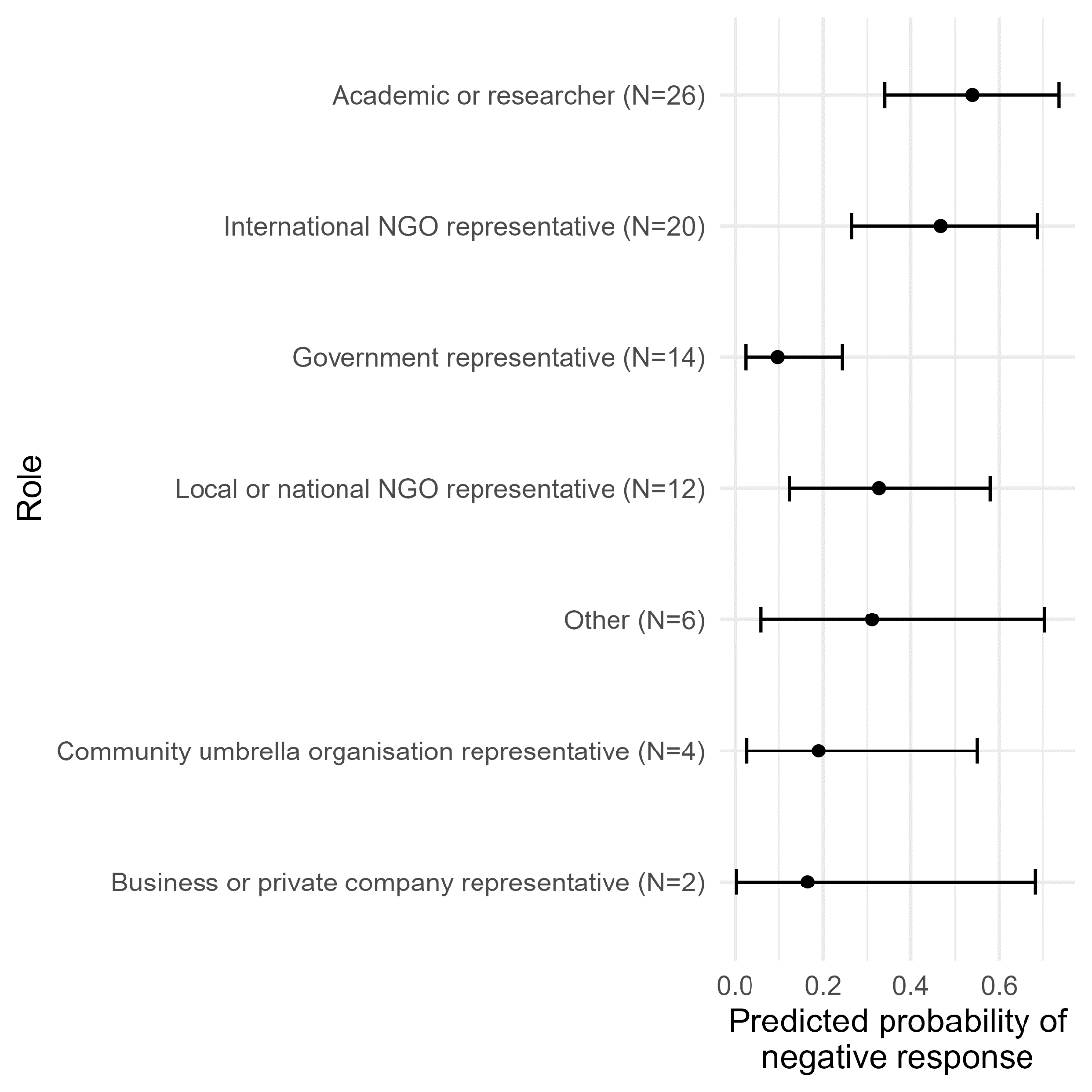


Figure 6. Variation in the predicted probability of a “negative” response regarding initiative complexity between expert groups. Points represent the mean prediction and whiskers the 95% credibility interval. If the credibility interval for one group did not overlap with the predicted probability for another group, we considered this a credible difference.


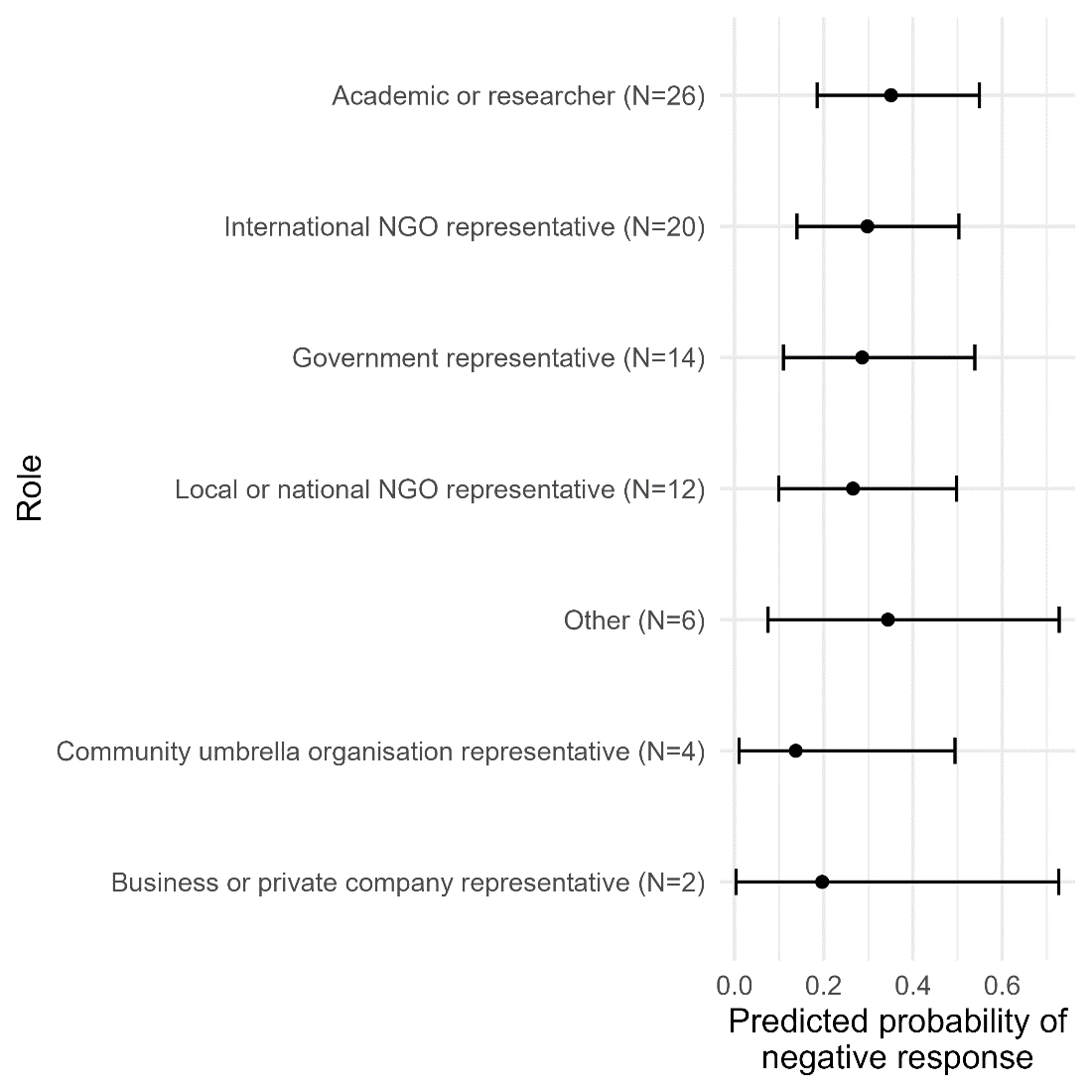


Figure 7. Variation in the predicted probability of a “negative” response regarding initiative observability between expert groups. Points represent the mean prediction and whiskers the 95% credibility interval. If the credibility interval for one group did not overlap with the predicted probability for another group, we considered this a credible difference.


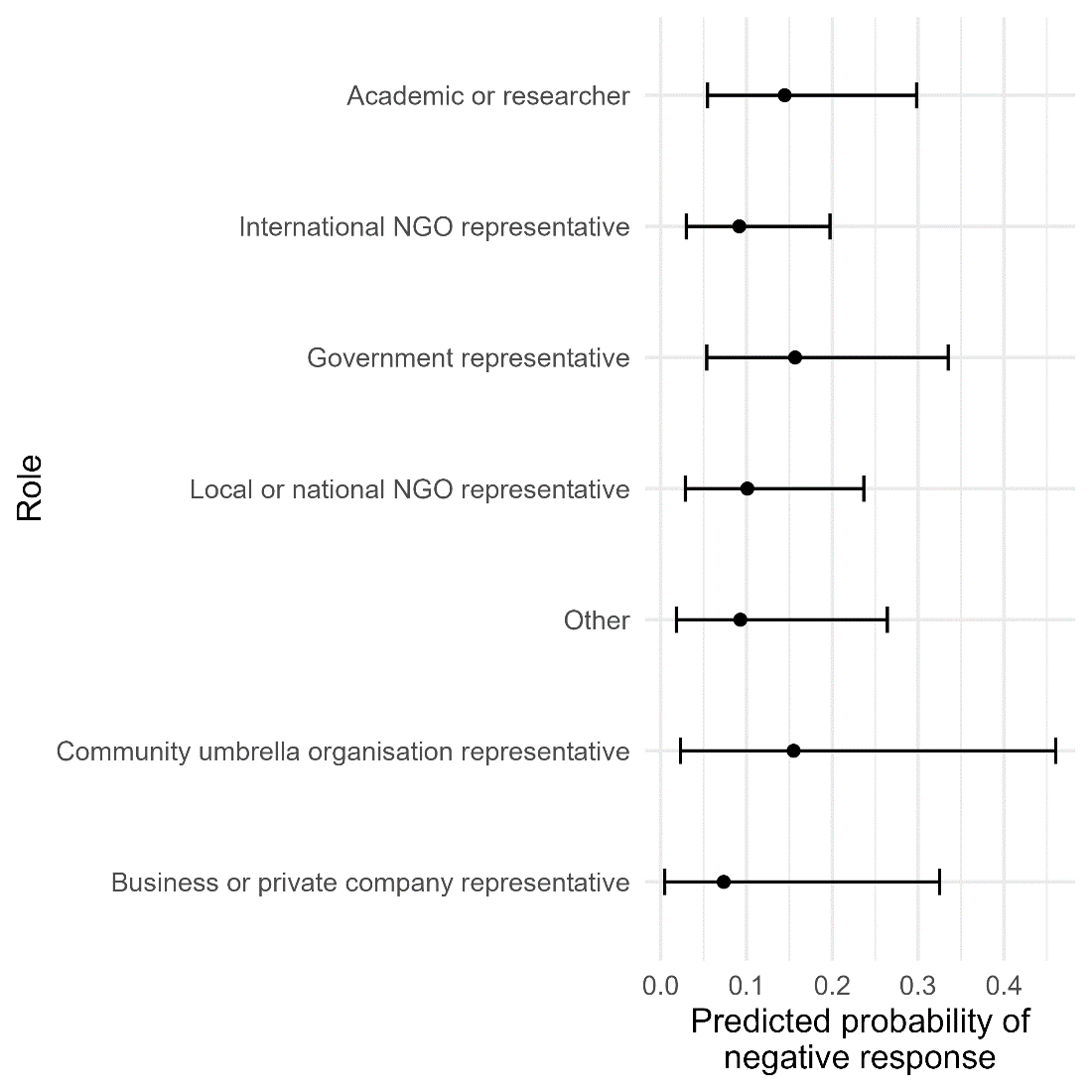


Figure 8. Variation in the predicted probability of a “negative” response regarding initiative relative advantage between expert groups. Points represent the mean prediction and whiskers the 95% credibility interval. If the credibility interval for one group did not overlap with the predicted probability for another group, we considered this a credible difference.


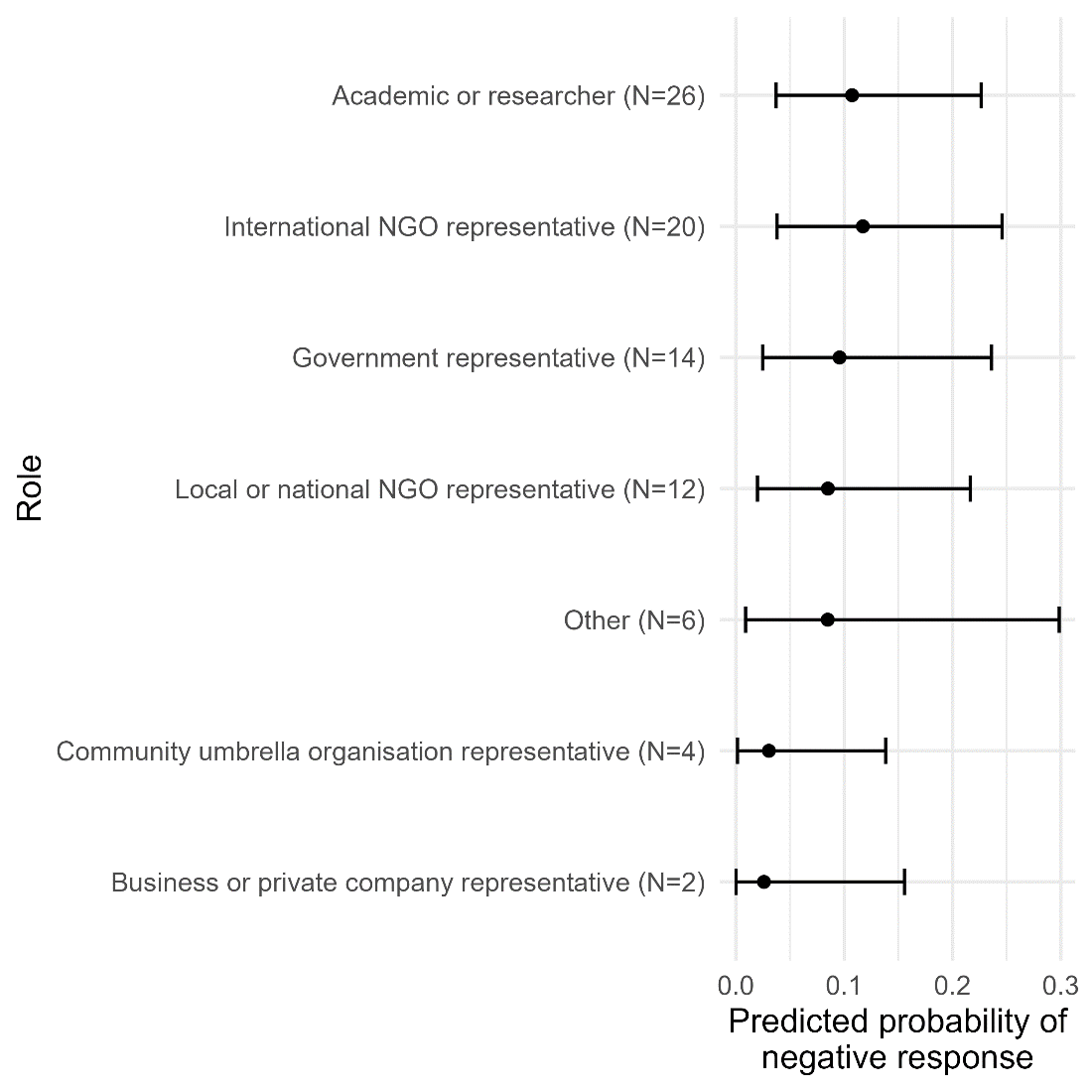


Figure 9. Variation in the predicted probability of a “negative” response regarding initiative overall relative advantage between expert groups. Points represent the mean prediction and whiskers the 95% credibility interval. If the credibility interval for one group did not overlap with the predicted probability for another group, we considered this a credible difference.


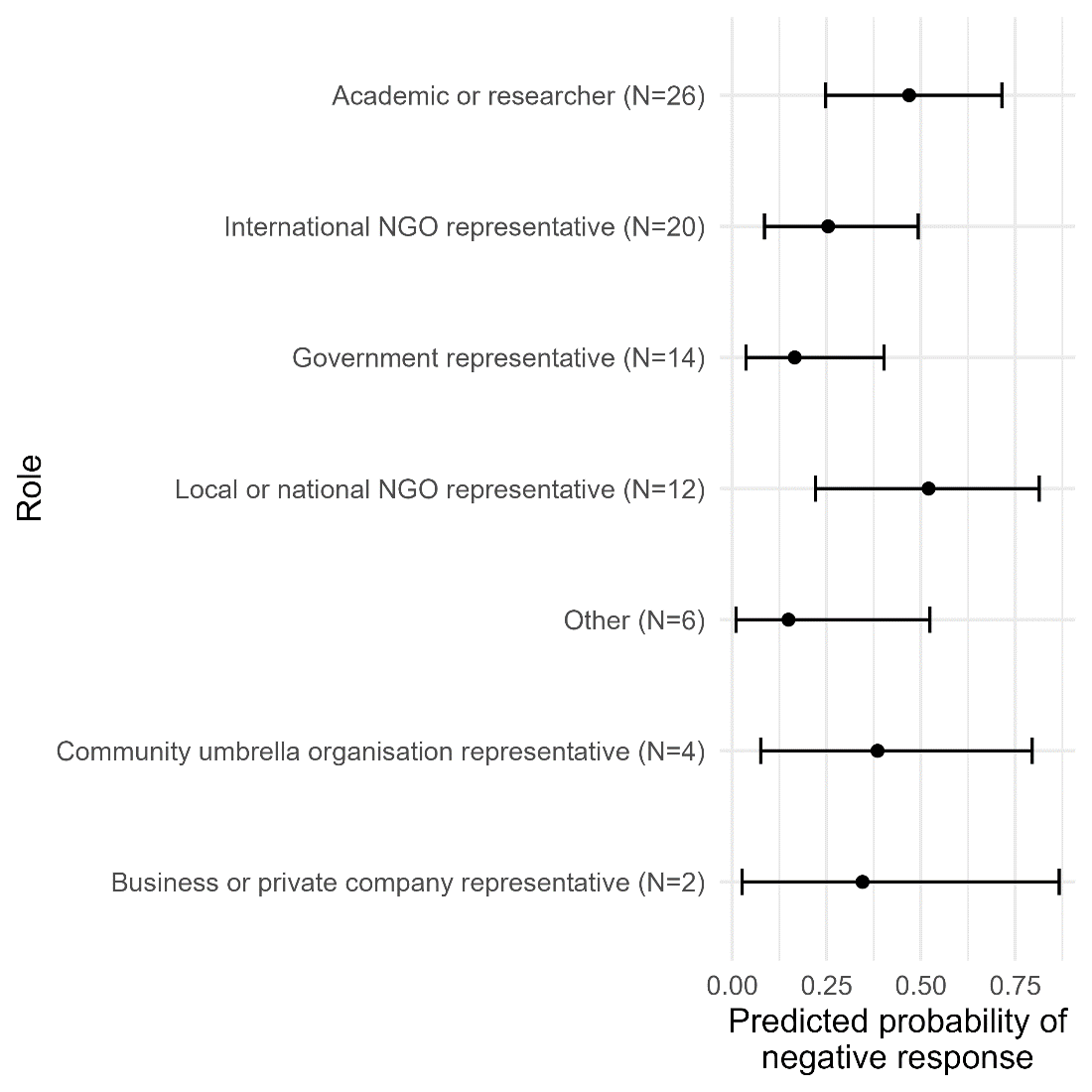


Figure 10. Variation in the predicted probability of a “negative” response regarding the fair distribution of costs and benefits within participating local groups between expert groups. Points represent the mean prediction and whiskers the 95% credibility interval. If the credibility interval for one group did not overlap with the predicted probability for another group, we considered this a credible difference.


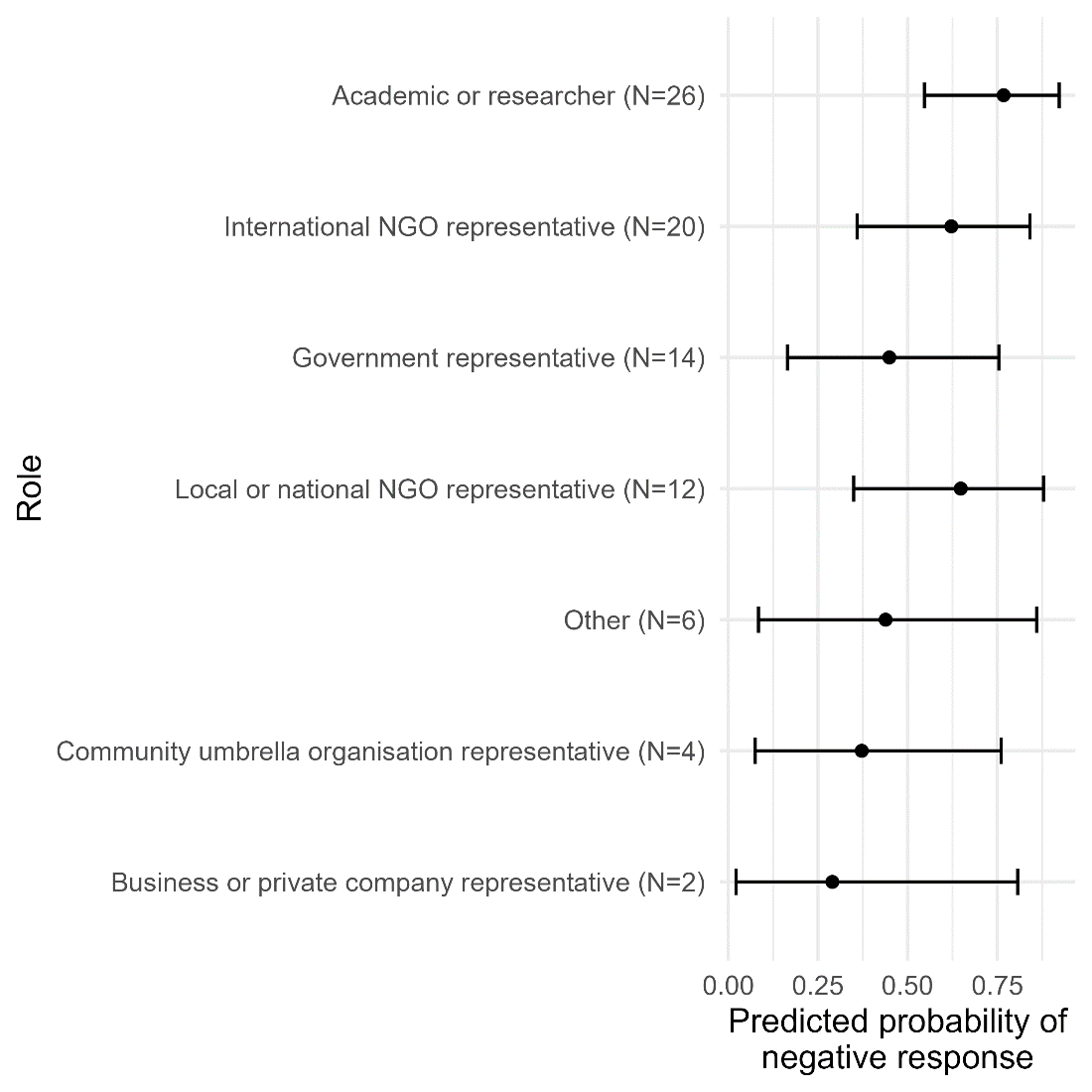


Figure 11. Variation in the predicted probability of a “negative” response regarding the fair distribution of costs and benefits between participating local groups and non-participating residents between expert groups. Points represent the mean prediction and whiskers the 95% credibility interval. If the credibility interval for one group did not overlap with the predicted probability for another group, we considered this a credible difference.


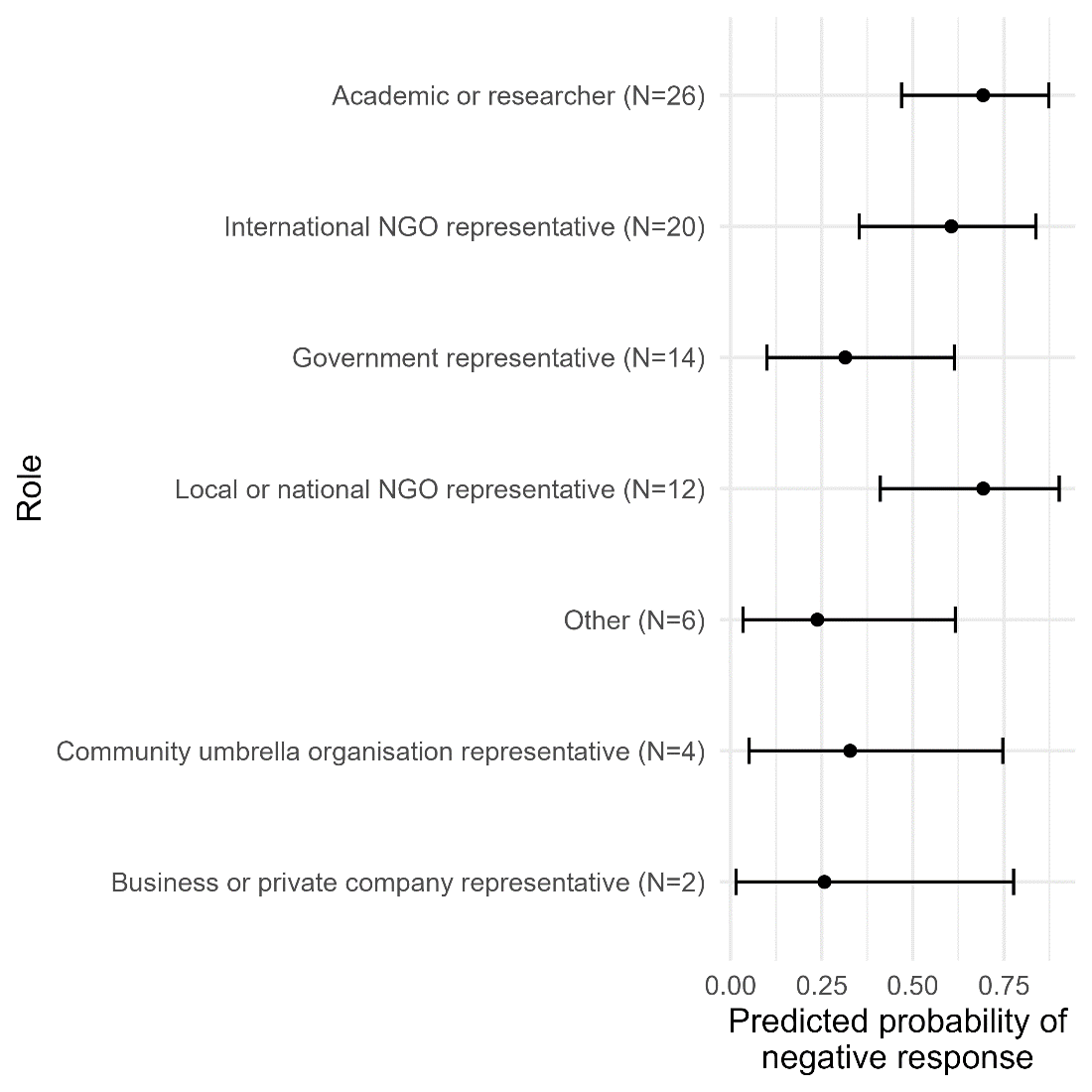


Figure 12. Variation in the predicted probability of a “negative” response regarding equal decision-making within local groups between expert groups. Points represent the mean prediction and whiskers the 95% credibility interval. If the credibility interval for one group did not overlap with the predicted probability for another group, we considered this a credible difference.


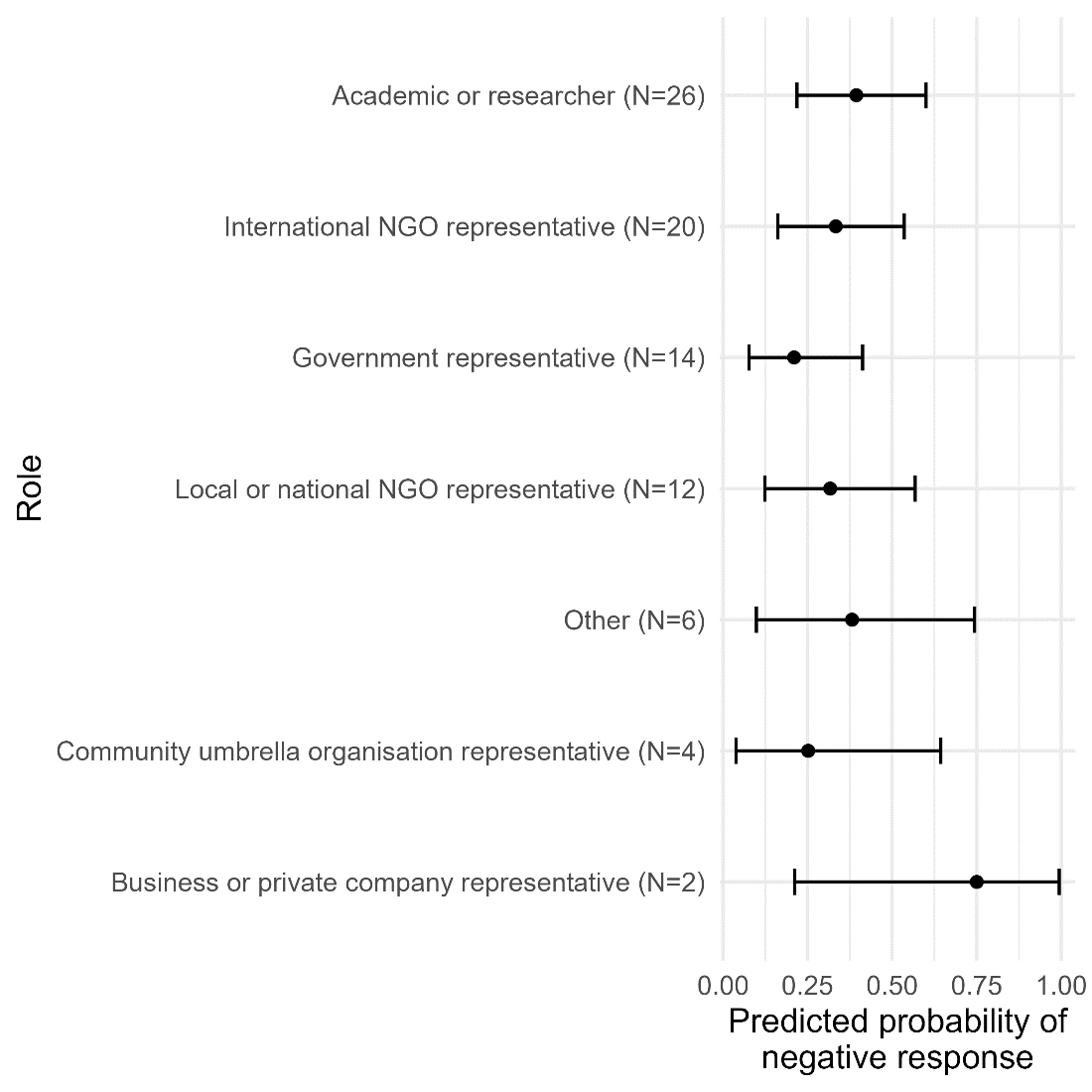


Figure 13. Variation in the predicted probability of a “negative” response regarding initiative compatibility with local needs between expert groups. Points represent the mean prediction and whiskers the 95% credibility interval. If the credibility interval for one group did not overlap with the predicted probability for another group, we considered this a credible difference.


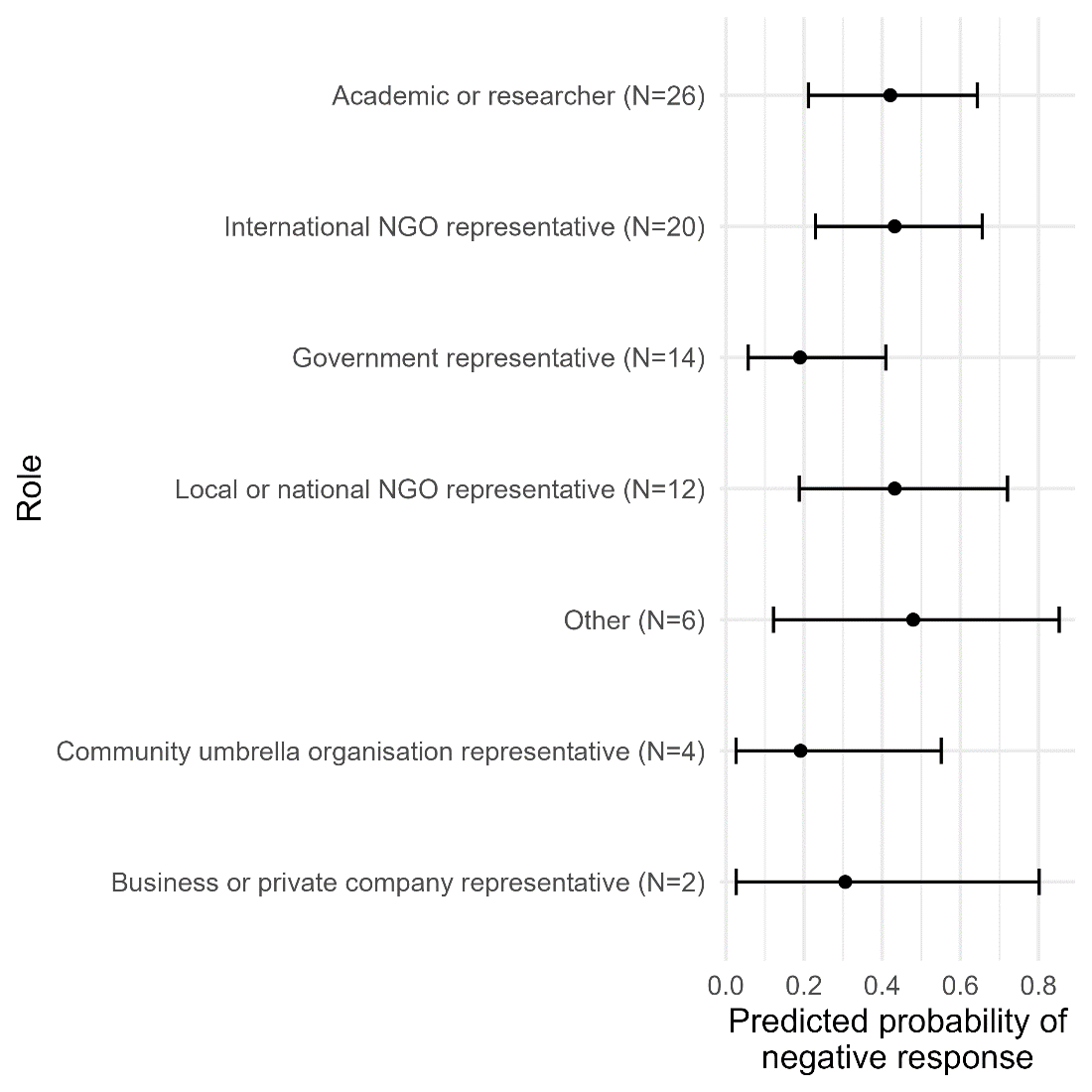


Figure 14. Variation in the predicted probability of a “negative” response regarding initiative flexibility between expert groups. Points represent the mean prediction and whiskers the 95% credibility interval. If the credibility interval for one group did not overlap with the predicted probability for another group, we considered this a credible difference.


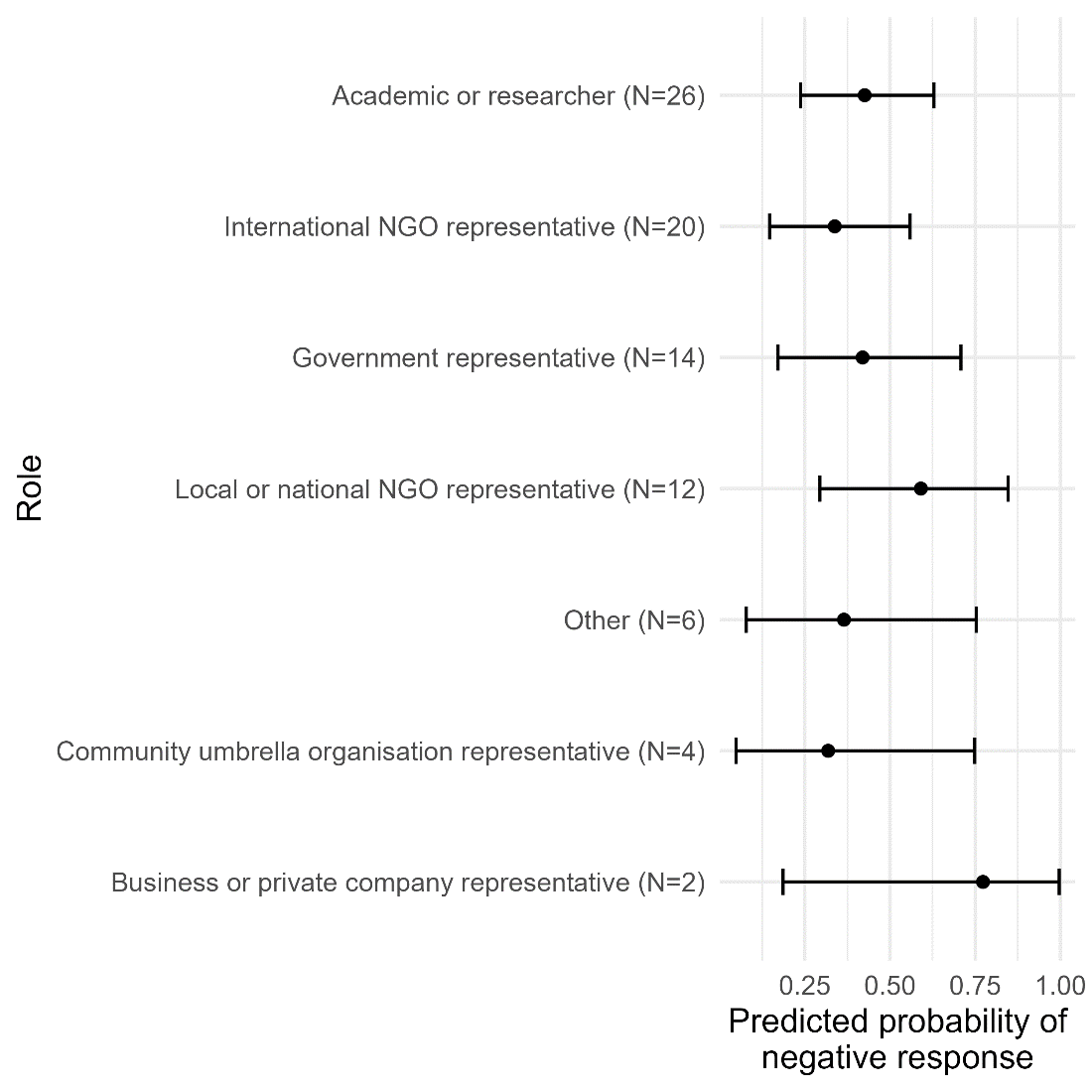


Figure 15. Variation in the predicted probability of a “negative” response regarding recognition of land rights between expert groups. Points represent the mean prediction and whiskers the 95% credibility interval. If the credibility interval for one group did not overlap with the predicted probability for another group, we considered this a credible difference.


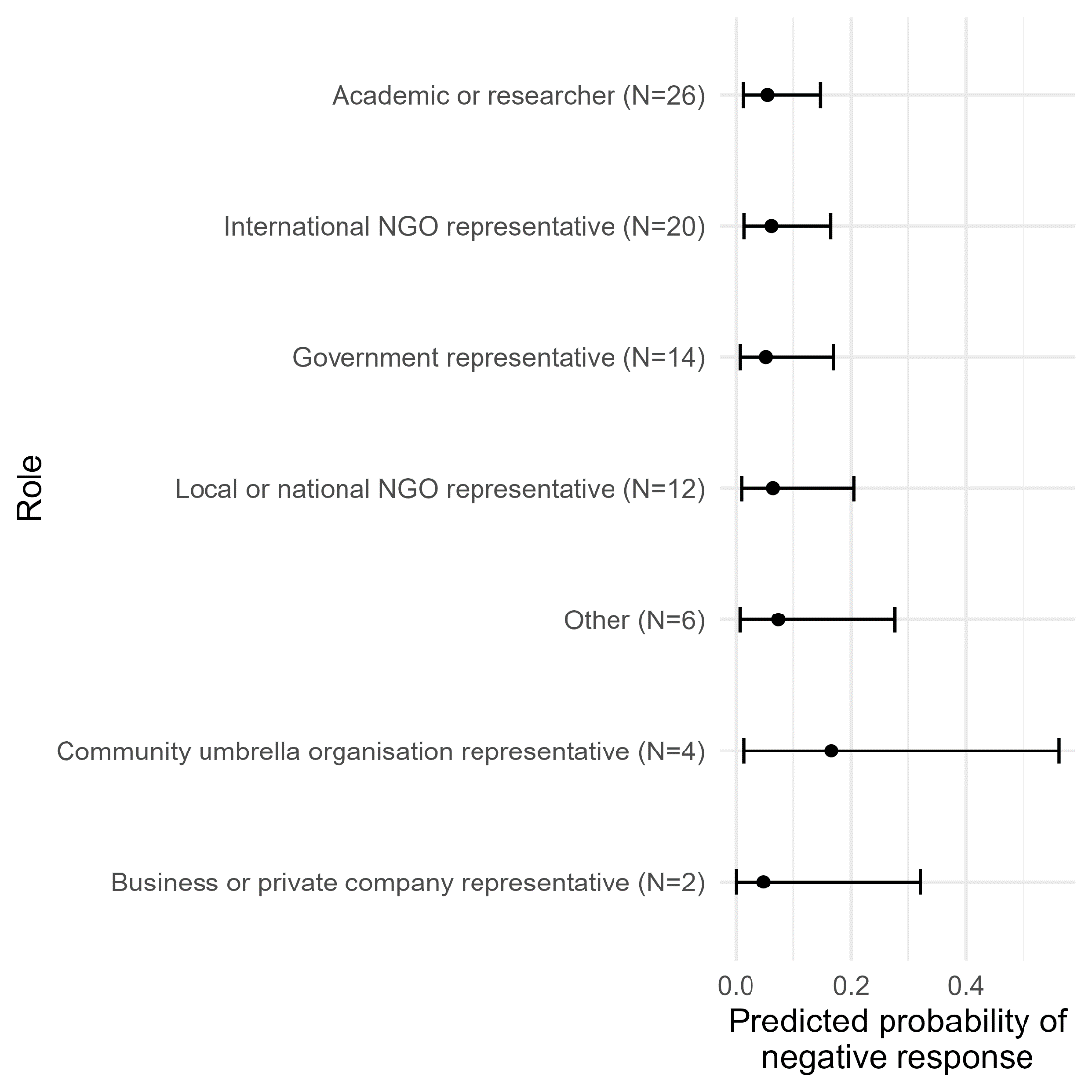


Figure 16. Variation in the predicted probability of a “negative” response regarding recognition of natural resource rights between expert groups. Points represent the mean prediction and whiskers the 95% credibility interval. If the credibility interval for one group did not overlap with the predicted probability for another group, we considered this a credible difference.


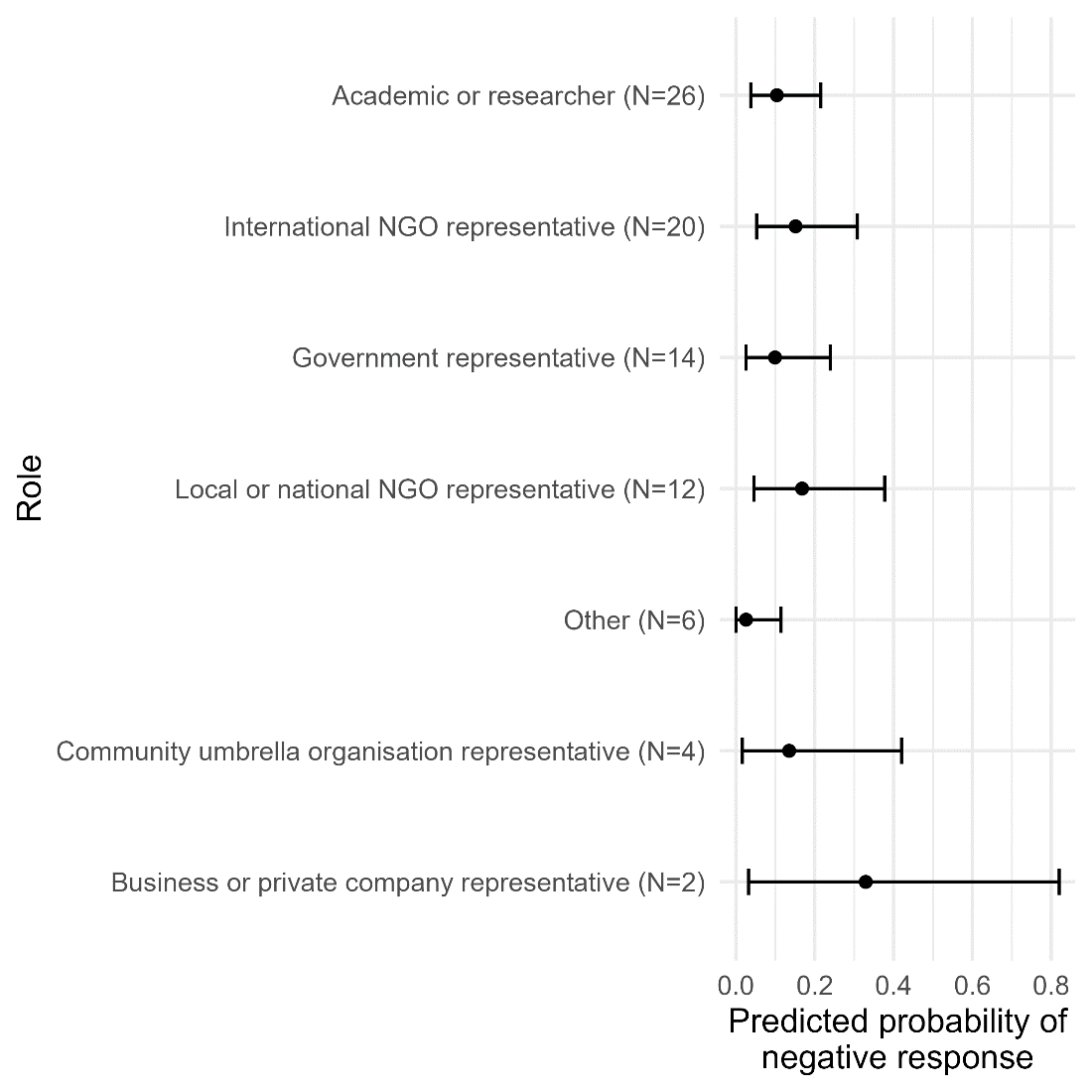


Figure 17. Variation in the predicted probability of a “negative” response regarding supportive national policies between expert groups. Points represent the mean prediction and whiskers the 95% credibility interval. If the credibility interval for one group did not overlap with the predicted probability for another group, we considered this a credible difference.


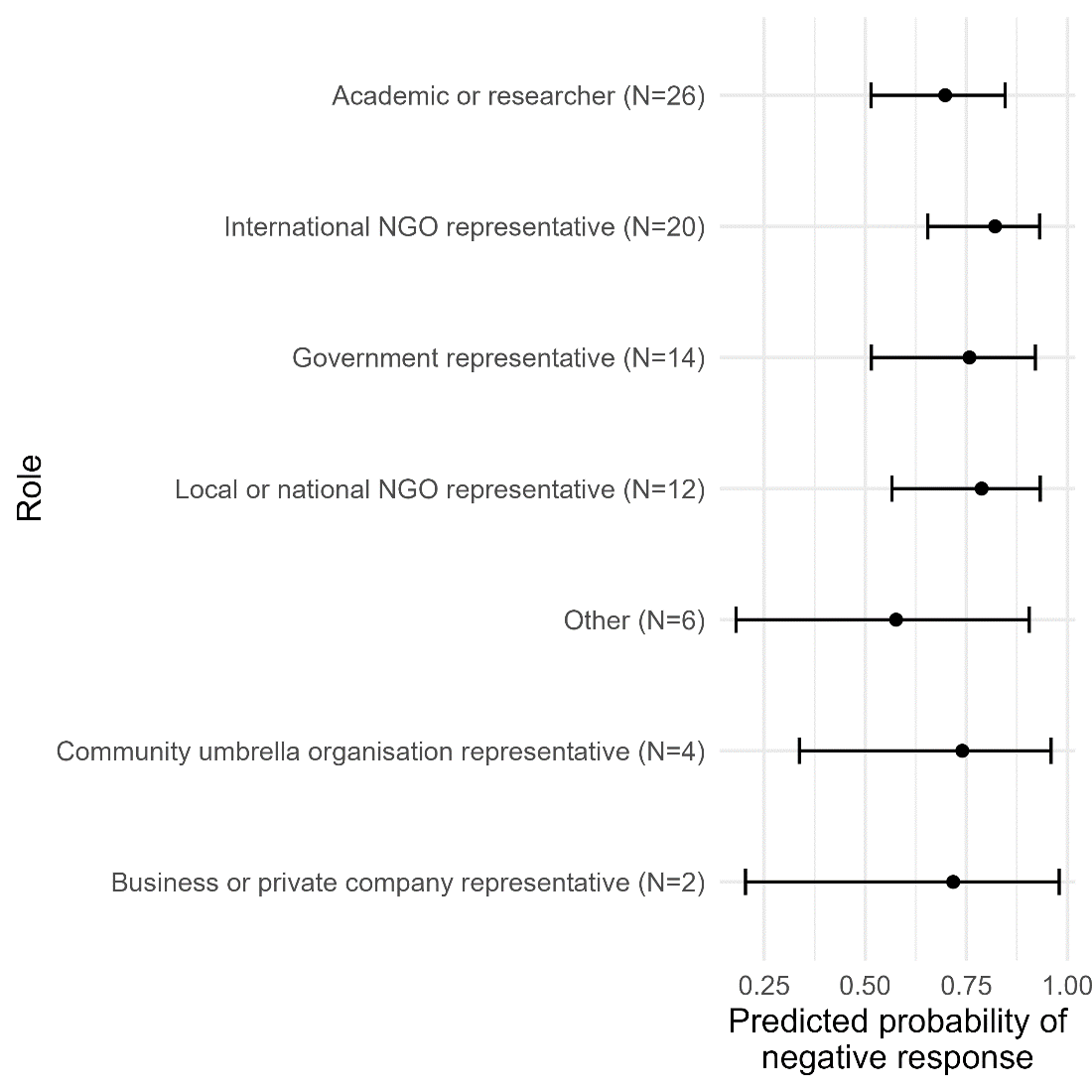


Figure 18. Variation in the predicted probability of a “negative” response regarding technical support between expert groups. Points represent the mean prediction and whiskers the 95% credibility interval. If the credibility interval for one group did not overlap with the predicted probability for another group, we considered this a credible difference.


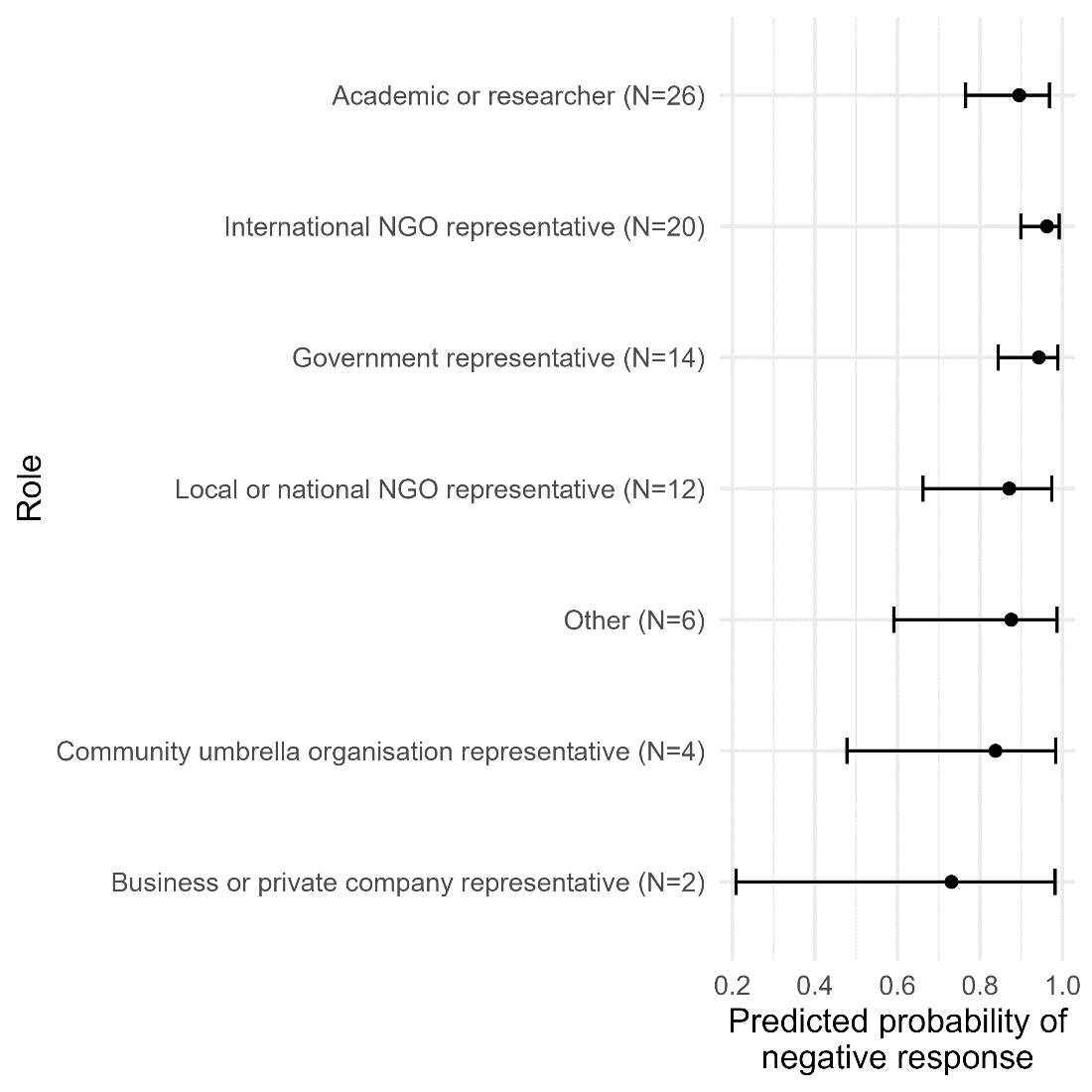


Figure 19. Variation in the predicted probability of a “negative” response regarding financial support between expert groups. Points represent the mean prediction and whiskers the 95% credibility interval. If the credibility interval for one group did not overlap with the predicted probability for another group, we considered this a credible difference.


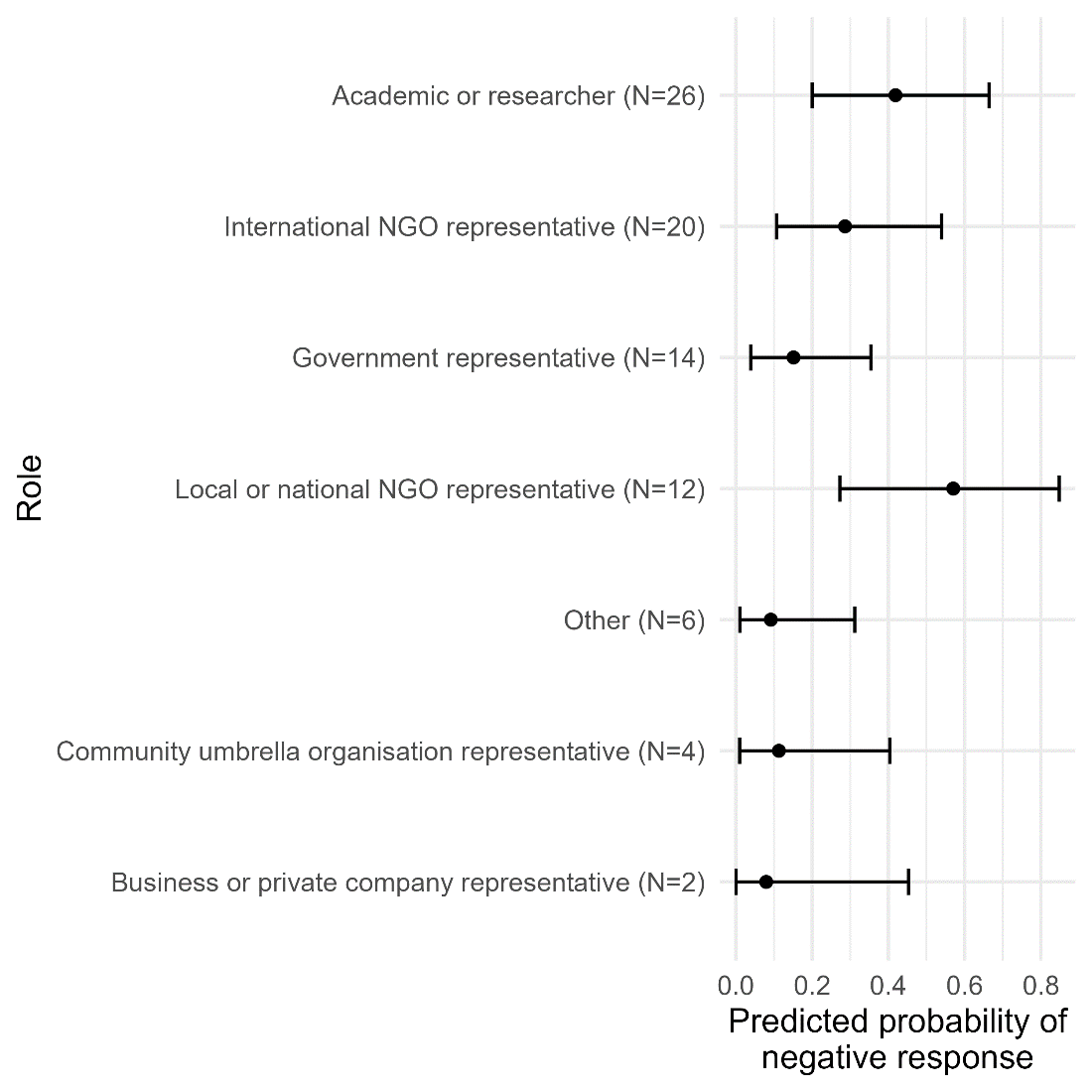


Figure 20. Variation in the predicted probability of a “negative” response regarding control of adoption decisions between expert groups. Points represent the mean prediction and whiskers the 95% credibility interval. If the credibility interval for one group did not overlap with the predicted probability for another group, we considered this a credible difference.


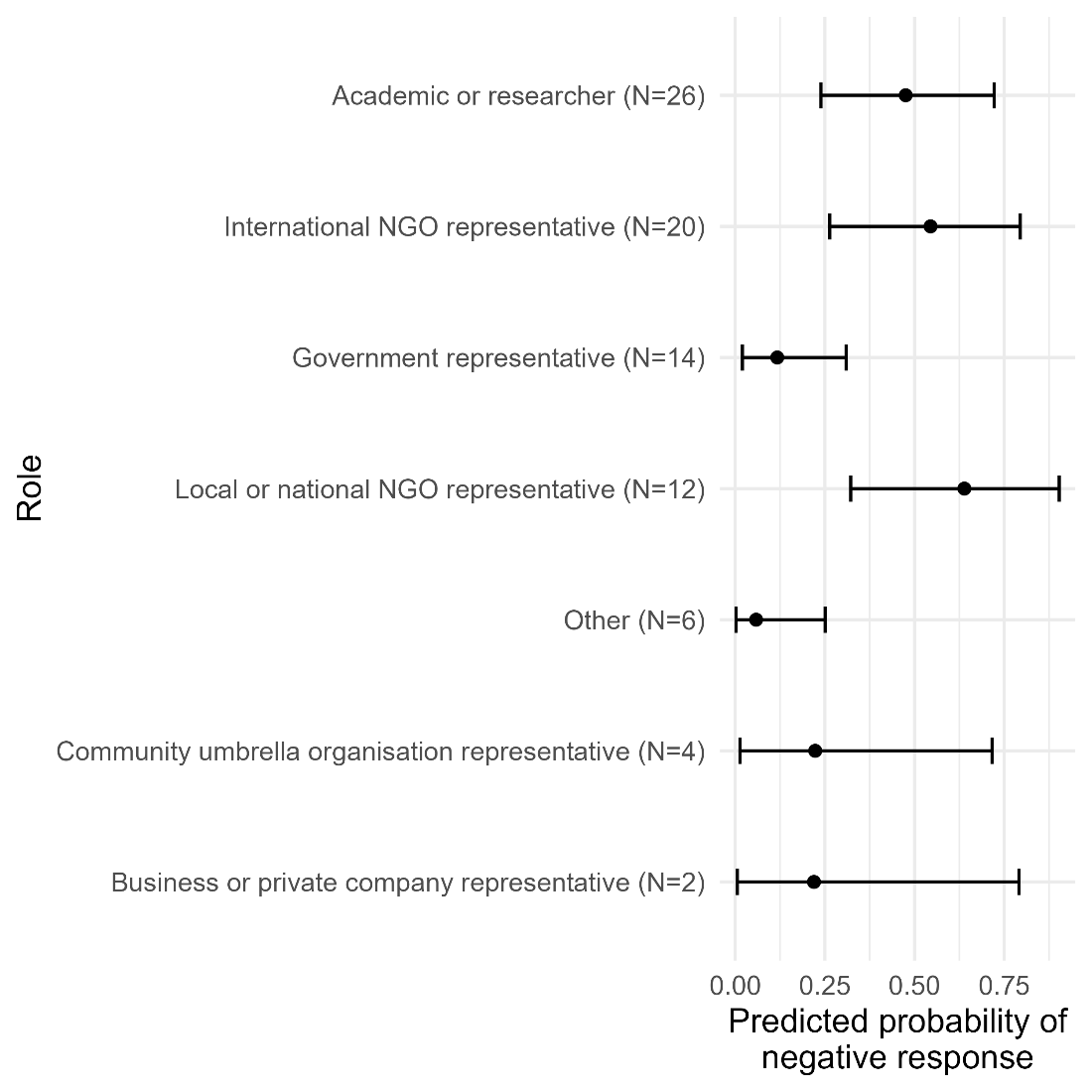


Figure 21. Variation in the predicted probability of a “negative” response regarding leadership of the adoption process between expert groups. Points represent the mean prediction and whiskers the 95% credibility interval. If the credibility interval for one group did not overlap with the predicted probability for another group, we considered this a credible difference.

## Appendix 11: Techniques for more accurate expert elicitation

In general, a substantial body of research has examined the utility and limitations of expert elicitation methods. For example, typical markers of expertise – such as years of experience and self or peer recommendation – can be poor predictors of the accuracy of judgements under high uncertainty (e.g., Burgman et al. 2011; Hemming et al. 2018b). A range of techniques has been developed to improve expert judgement accuracy. These include prompting experts to consider counterarguments, ensuring neutral problem framing, eliciting repeated judgments, restricting judgments to experts’ domains, and openly discussing values and motivational biases (Wintle et al. 2022). Similarly, group-based expert elicitation should encourage critical thinking and counterfactual argumentation rather than consensus, include diverse participants, and foster accountability (Wintle et al. 2022). These techniques, as well as more structured protocols (e.g., the IDEA protocol (Hemming et al. 2018a)), could be integrated into future attempts to identify scaling bottlenecks.

## Appendix 12: Multi-actor scaling bottlenecks

Multiple actors beyond local communities – including funders, governments, and NGOs – are involved in the adoption process. Each actor group will likely face different barriers to adopting or supporting initiatives, all of which might need to be overcome to effectively scale initiatives. For example, government agencies might face resource constraints that limit their ability to support communities in adopting initiatives. Therefore, identifying and overcoming barriers faced by different actors, including via more participatory research, could help develop more holistic scaling strategies.

## References

Blaikie P. 2006. Is small really beautiful? Community-based natural resource management in Malawi and Botswana. World Development **34**:1942-1957.

Burgman MA, McBride M, Ashton R, Speirs-Bridge A, Flander L, Wintle B, Fidler F, Rumpff L, Twardy C. 2011. Expert status and performance. PLOS ONE **6**:e22998.

Bürkner P. 2017. brms: An R package for Bayesian multilevel models using Stan. Journal of Statistical Software **80**:1-28.

Cleaver F. 1999. Paradoxes of participation: Questioning participatory approaches to development. Journal of International Development **11**:597-612.

Convention on Biological Diversity (CBD). 2018. Decision adopted by the conference of the parties to the Convention on Biological Diversity. 14/8 Protected areas and other effective area-based conservation measures.

Davis A-L, Blomley T, Homer G, Sommerville M, Nelson F. 2020. Community-based natural resource management in Zambia: A review of institutional reforms and lessons from the field. Washington, DC.

Depaoli S, Van de Schoot R. 2017. Improving transparency and replication in Bayesian statistics: The WAMBS-Checklist. Psychological Methods **22**:240-261.

Food and Agriculture Organization (FAO). 2019. Status of community-based forestry and forest tenure in the United Republic of Tanzania.

Freitas CT, Lopes PFM, Campos-Silva JV, Noble MM, Dyball R, Peres CA. 2020. Co-management of culturally important species: A tool to promote biodiversity conservation and human well-being. People and Nature **2**:61-81.

Government of Malawi. 2001. A field guide for Community Based Forest Management.

Hemming V, Burgman MA, Hanea AM, McBride MF, Wintle BC. 2018a. A practical guide to structured expert elicitation using the IDEA protocol. Methods in Ecology and Evolution **9**:169-180.

Hemming V, Walshe TV, Hanea AM, Fidler F, Burgman MA. 2018b. Eliciting improved quantitative judgements using the IDEA protocol: A case study in natural resource management. PLOS ONE **13**:e0198468.

Intergovernmental Science-Policy Platform on Biodiversity and Ecosystem Services (IPBES). 2019. Annex I: Glossary of the Global assessment report on biodiversity and ecosystem services of the Intergovernmental Science-Policy Platform on Biodiversity and Ecosystem Services.

Kazoora C, Irumba D, Smith N, Campese J. 2020. A review of collaborative forest management in Uganda.

Lyons A. 2000. An effective monitoring framework for community based natural resource management: A case study of the ADMADE program in Zambia. Pages 1-148. University of Florida.

Mauambeta DDC, Kafakoma RPG. 2010. Community based natural resource management stocktaking assessment: Malawian profile.

United Republic of Tanzania (URT). 2007. Community-based Forest Management Guidelines. Dar es Salaam, Tanzania.

Wintle BC, Mukherjee N, Hemming V, Canessa S, McBride M. 2022. Improving the reliability of judgements. Pages 133-176. Transforming Conservation: A Practical Guide to Evidence and Decision Making. Open Book Publishers.
